# Recurrent neural chemical reaction networks trained to switch dynamical behaviours through learned bifurcations

**DOI:** 10.1098/rsif.2026.0169

**Published:** 2026-10-14

**Authors:** Alexander Dack, Tomislav Plesa, Thomas E. Ouldridge

**Affiliations:** ^1^ Department of Bioengineering and Imperial College Centre for Engineering Biology, Imperial College London, London, UK; ^2^ Department of Applied Mathematics and Theoretical Physics, University of Cambridge, Cambridge, UK

**Keywords:** chemical reaction networks, dynamical systems, artificial neural networks

## Abstract

Both natural and synthetic chemical systems not only exhibit a range of non-trivial dynamics, but also transition between qualitatively different dynamical behaviours as environmental parameters change. Such transitions are called bifurcations. Here, we show that recurrent neural chemical reaction networks (RNCRNs), a class of chemical reaction networks based on recurrent artificial neural networks that can be trained to reproduce a given dynamical behaviour, can also be trained to exhibit bifurcations. First, we show that RNCRNs can inherit some bifurcations defined by smooth ordinary differential equations (ODEs). Second, we demonstrate that the RNCRN can be trained to infer bifurcations that allow it to approximate different target behaviours within different regions of parameter space, without explicitly providing the bifurcation itself in the training. These behaviours can be specified using target ODEs that are discontinuous with respect to the parameters, or even simply by specifying certain desired dynamical features in certain regions of the parameter space. To achieve the latter, we introduce an ODE-free algorithm for training the RNCRN to display designer oscillations, such as a heart-shaped limit cycle or two coexisting limit cycles.

## 1. Introduction

It is common to observe biological entities transition between dynamical behaviours as they process information from their environment [1] or as they proceed through their developmental cycle [2]. For example, it is well known in cellular biology that cells differentiate, i.e. change type, to specialize into particular functions [3]. Expressed in the nomenclature of dynamical systems, cells are moving between different dynamical regimes capable of different behaviours that govern their function. In some dynamical systems, there are external factors, called *parameters*, that determine the dynamical regime. In biochemistry, such parameters may be interpreted as the reaction rate coefficients or the concentrations set by exogenous chemostat.

Switching of dynamical behaviours as parameters are changed is studied in a branch of mathematics called *bifurcation theory* [4]. This general theory has categorized the types of transitions into canonical forms [4] and been applied broadly to understand a range of biological phenomena, including snapping mechanism in the Venus flytrap [5], predator–prey relationships in ecosystems [6], and to model neural networks in the brain [7–11]. Of particular relevance to this work is the investigation of cellular regulation at the biochemical level through the lens of bifurcation theory [12–16].

Aside from being used to model natural biological phenomena, dynamical systems are also used in the fields of synthetic biology and nucleic acid nanotechnology, which have been successful at engineering biological systems with desired dynamical behaviours. Examples include neural network classification algorithms [17–24] and attractor orbits [25–27], which have been built in a range of molecular media. Given that individual dynamical behaviours have been realized successfully, a natural question arises: can one combine these components to engineer biological systems with the complicated switching behaviour, or bifurcations, seen in the native systems?

In this context, on the one hand, some research has focused on eliminating undesirable bifurcations in engineered systems [28,29], and studying how sudden and unexpected changes in dynamical behaviours can lead to catastrophic system failures [30]. For example, such bifurcations are known to lead to hazardous events in synthetic biology control systems [31]. On the other hand, there has also been a growing interest in bifurcation-inspired design paradigms [32,33], given that bifurcations are potentially a highly efficient solution to achieving large changes in output for only a small change in input. Indeed, there has been both experimental [34,35] and theoretical work [36–42] investigating the construction of bifurcations in biochemical systems. In particular, mathematical methods for designing *chemical reaction networks* (CRNs), mathematical models of chemistry, that implement a given bifurcation are developed in [43] and [44]. All of these methods take as input a sufficiently smooth dynamical system with desired bifurcation structure, which is then converted into a CRN [31,45–50].

In this paper, we present methods to construct CRNs that use bifurcations to interpolate between regions of desired dynamics, for which only the desired dynamics need to be specified ahead of training. In particular, we base our methods on the *recurrent neural chemical reaction network* (RNCRN)—a class of CRNs that operates as a recurrent neural network, whose fast-acting chemical perceptrons can be trained to approximate arbitrary dynamics [51].

We first demonstrate that the RNCRN with sufficiently many species can be adapted for training to reproduce classical bifurcations from a given smooth parameter-dependent dynamical system, in line with the capabilities of more minimal methods [43,44]. We then demonstrate that the RNCRN can be further adapted and trained to approximate and merge different behaviours from a given dynamical system which depends on its parameters discontinuously. Finally, we introduce an algorithm for training the RNCRN to approximate limit cycles specified only by the shape of the limit cycle itself, rather than via ordinary differential equations (ODEs) that generate a limit cycle, which enables the construction of a variety of behaviours from sparse data. Motivated by the success of chemical classifiers [52–60], we use this algorithm to develop an extended RNCRN that can sense its local environment, perform a nonlinear classification and then toggle between dynamical regimes accordingly. We note that these discontinuous and sparsely defined target systems showcase the RNCRN’s ability to extrapolate outside, and interpolate between, training data to create a self-consistent vector field that matches the intended dynamical behaviour.

This paper is structured in five parts. In §2, some background theory is presented. In §3 and appendix A, we demonstrate that RNCRNs can approximate bifurcations of a given smooth dynamical system. In §4, we design RNCRNs that approximate piecewise systems, i.e. non-smooth systems that have different dynamical behaviours according to some externally imposed condition. Finally, in §5 and appendix C, we demonstrate that RNCRNs can be used to construct dynamical behaviours entirely from relatively sparse data and demonstrate switching between dynamical regimes in this context.

## 2. Background

**CRNs.** A CRN under mass-action kinetics is a set of chemical species which interact via a set of reactions whose speed is parametrized by rate coefficients [61]. This mathematical abstraction of chemistry is commonly used to model (bio)chemical systems in synthetic biology and DNA nanotechnology, including toggle switches in gene regulatory networks [62], chemotaxis in *Escherichia coli* [63] and biological pattern formation [64].

For example, CRN

(2.1) $$\begin{array}{r} X_{1}\overset{\alpha_{1}\;}{\to }\emptyset ,\;\;\;X_{1}\overset{\alpha_{2}\;}{\to }X_{1}+X_{1},\;\;\;X_{1}+X_{1}\overset{\alpha_{3}/2\;}{\to }\emptyset \end{array}$$

describes a single chemical species $X_{1}$ that is involved in degradation, autocatalytic production and self-annihilation with the (dimensionless) rate coefficients, respectively, given by $\alpha_{1},\alpha_{2},\alpha_{3}/2>0$, and where ∅ represents some chemical species that are of no interest. Under the assumption that chemical reactions occur in a well-mixed container, and that the chemical species are in high abundance, CRNs can be modelled deterministically with mass-action *reaction-rate equations* (RREs)—a system of ODEs with polynomials on the right-hand side [61]. For example, CRN equation (2.1) has the corresponding RRE

(2.2) $$\begin{array}{rl} \frac{dx_{1}}{dt} & =(\alpha_{2}-\alpha_{1})x_{1}-\alpha_{3}x_{1}^{2}, \end{array}$$

where $x_{1}=x_{1}(t)\geq 0$ is the time-varying molecular concentration of the chemical species $X_{1}$.

**RNCRNs.** In prior work, the authors have introduced a CRN based on artificial neural networks (ANNs), called the RNCRN [51], and used it to approximate the dynamics of well-behaved ODE systems. In particular, given a target system of ODEs with suitably smooth right-hand side (vector field), given by

(2.3) $$\begin{array}{rl} \frac{d{\bar{x}}_{i}}{dt} & =f_{i}({\bar{x}}_{1},\ldots ,{\bar{x}}_{n}),\;\;{\bar{x}}_{i}(0)\geq 0,\;\;\;i=1,2,\ldots ,N, \end{array}$$

there exists an RNCRN with the RREs

(2.4) $$\begin{array}{rlrl} \frac{dx_{i}}{dt} & =\beta_{i}+x_{i}\sum_{j=1}^{M}\alpha_{i,j}y_{j}, & & \;\;x_{i}(0)\geq 0,\;\;\;i=1,2,\ldots ,N\\ \text{and}\;\frac{dy_{j}}{dt} & =\frac{\gamma }{\mu }+y_{j}\left(\sum_{i=1}^{N}\frac{\omega_{j,i}}{\mu }x_{i}+\frac{\theta_{j}}{\mu }\right)-\frac{1}{\mu }y_{j}^{2}, & & \;\;y_{j}(0)\geq 0,\;\;\;j=1,2,\ldots ,M, \end{array}$$

such that the concentration $x_{i}(t)$ approximates the target variable ${\bar{x}}_{i}(t)$ from equation (2.3) arbitrarily well. In particular, parameters $\beta_{i},\gamma >0$ from equation (2.4) are positive, while $\alpha_{i,j},\omega_{j,i},\theta_{j}\in R$ can take any real value; furthermore, parameter μ>0 controls how fast the variables $y_{j}(t)$ are with respect to $x_{i}(t)$. For suitable values of these parameters, with μ>0 sufficiently small, i.e. with $y_{j}(t)$ sufficiently fast compared to $x_{i}(t)$, equation (2.4) approximates a given target system equation (2.3) arbitrarily well. In order to attempt to find such parameter values, algorithm 1 is presented in [51].

**Algorithm 1. Algorithm for generating data to train RNCRNs to display two-dimensional stable limit cycles defined via the set of points $\left\{P_{1},P_{2},\ldots ,P_{D},P_{D+1}\right\}$.**

**Figure:**
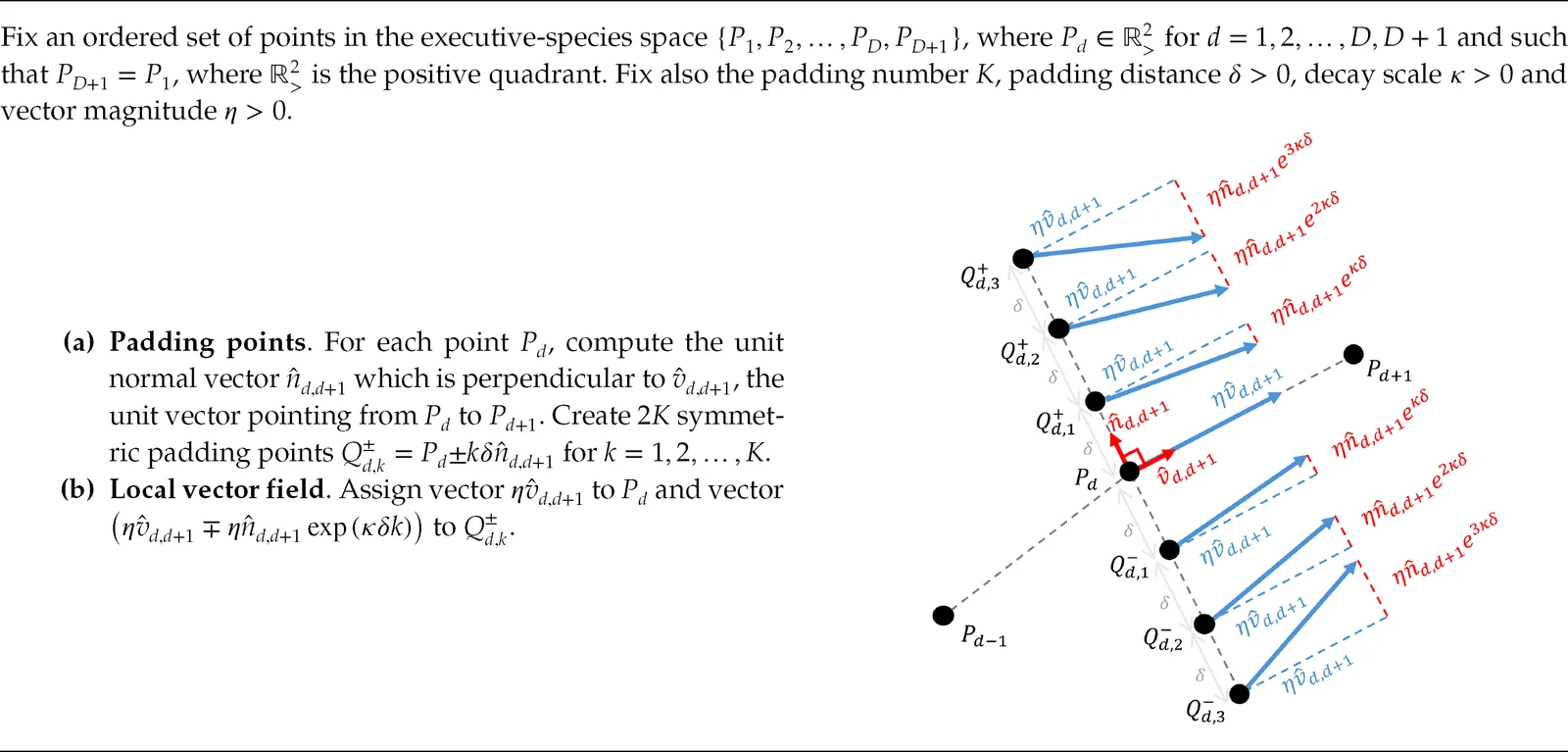

The concentrations $x_{i}(t)$ and $y_{j}(t)$, respectively, correspond to two sets of chemical species: the *executive* species $X_{i}$ which manifest the target dynamical behaviour and the *chemical-perceptron* species $Y_{j}$ which control the dynamics of the executive species through auxiliary reactions, which we illustate in figure 1. Chemical perceptrons $Y_{j}$ are initially introduced in [56] and proposed as a chemical realization of a feed-forward chemical neural network used for classification tasks, processing static input concentrations $x_{i}$ to reach an equilibrium concentration $y_{j}$ that corresponds to a classification decision. In the RNCRN, however, we introduce feedback reactions to the executive species $X_{i}$ (see caption of figure 1) that allow the output of a single layer of chemical perceptrons to dictate the dynamics of the executive species as they all evolve together.

**Figure 1.**
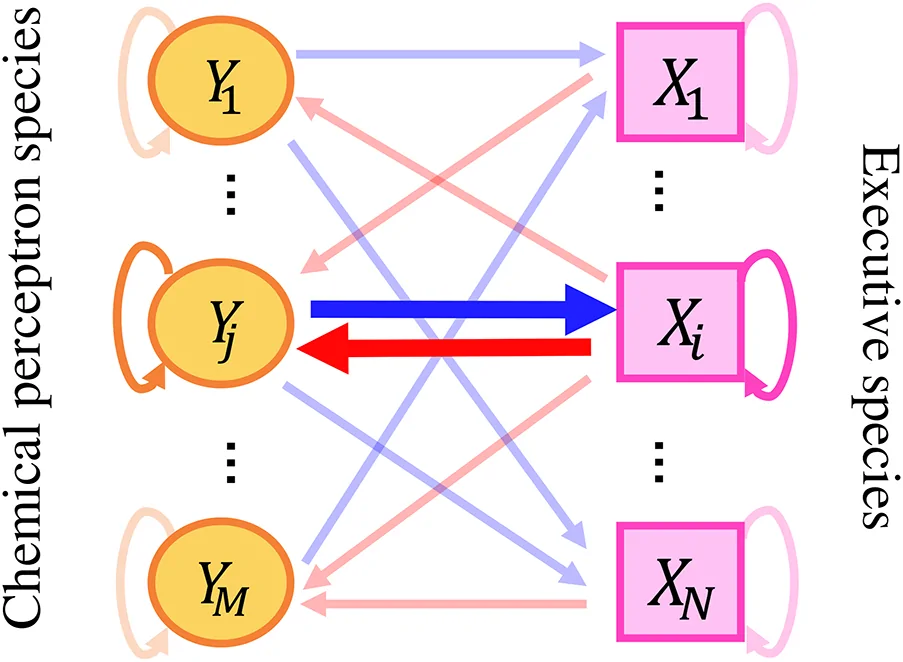
A graphical visualization of the RNCRN underlying the RREs equation (2.4), as introduced in [51]. Orange circles represent chemical-perceptron species $Y_{1},\ldots ,Y_{M}$, whereas magenta squares represent the executive species $X_{1},\ldots ,X_{N}$. Coloured arrows represent chemical reactions consistent with the RREs: blue arrows represent $X_{i}+Y_{j}\overset{|\alpha_{i,j}|\;}{\to }2X_{i}+Y_{j}$ or $X_{i}+Y_{j}\overset{|\alpha_{i,j}|\;}{\to }Y_{j}$ (according to the sign of $\alpha_{i,j}$), red arrows represent $X_{i}+Y_{j}\overset{|\omega_{j,i}|/\mu \;}{\to }X_{i}+2Y_{j}$ or $X_{i}+Y_{j}\overset{|\omega_{j,i}|/\mu \;}{\to }X_{i}$ (according to the sign of $\omega_{j,i}$), magenta arrows represent $\emptyset \overset{\beta_{i}\;}{\to }X_{i}$ and orange arrows represent three reactions $\emptyset \overset{\gamma /\mu \;}{\to }Y_{j}$, $2Y_{j}\overset{1/\mu \;}{\to }Y_{j}$ and one of $Y_{j}\overset{|\theta_{j}|/\mu \;}{\to }2Y_{j}$ or $Y_{j}\overset{|\theta_{j}|/\mu \;}{\to }\emptyset$ (according to the sign of $\theta_{j}$).

The parameter μ>0 in equation (2.4) controls the time-scale separation between the fast perceptrons and the slow executive species. Owing to the stability of the underlying chemical perceptrons [56], in the formal limit μ→0, the dynamics are governed by the *reduced* RNCRN with RREs

(2.5) $$\begin{array}{rl} \frac{d{\tilde{x}}_{i}}{dt} & =g_{i}({\tilde{x}}_{1},\ldots ,{\tilde{x}}_{N})=\beta_{i}+{\tilde{x}}_{i}\sum_{j=1}^{M}\alpha_{i,j}\sigma_{\gamma }\left(\sum_{k=1}^{N}\omega_{j,k}{\tilde{x}}_{k}+\theta_{j}\right),\;\;{\tilde{x}}_{i}(0)\geq 0,\;\;\;i=1,2,\ldots ,N, \end{array}$$

where $\sigma_{\gamma }$ is a chemical activation function defined as $\sigma_{\gamma }(x)=(x+\sqrt{x^{2}+4\gamma })/2$. In this reduced limit, the chemical perceptrons operate as a conventional ANN with an activation function for each perceptron that approximates the commonly used Rectified Linear Unit (ReLU) activation function, allowing training of the RNCRN’s parameters by standard methods. Loosely, algorithm 1 in [51] uses standard machine learning methods to find an ANN such that $g_{i}({\tilde{x}}_{1},\ldots ,{\tilde{x}}_{N})\approx f_{i}({\tilde{x}}_{1},\ldots ,{\tilde{x}}_{n})$, and then relaxes the assumption that μ=0 to find a non-zero μ>0 such that the full RNCRN equation (2.4) approximates the target system equation (2.3) to a sufficient accuracy. Let us note that in both algorithm 1, introduced in [51], and the new training algorithms introduced in this paper (see appendix D), we evaluate the vector fields at a discrete set of points and minimize the loss function over those points. Typically, the loss function is the mean squared error averaged between the reduced RNCRN approximation $g_{i}({\tilde{x}}_{1},\ldots ,{\tilde{x}}_{N})$ and the target vector field $f_{i}({\tilde{x}}_{1},\ldots ,{\tilde{x}}_{N})$ (or some simple transformation thereof).

**Bifurcations.** In equation (2.3), we have assumed that the target vector field $f=(f_{1},f_{2},\ldots ,f_{N})$ does not depend on any parameters. More broadly, we can relax this assumption by generalizing equation (2.3) to include a real parameter $\bar{\lambda }$, and consider the parameter-dependent target system

(2.6) $$\begin{array}{rl} \frac{d{\bar{x}}_{i}}{dt} & =f_{i}({\bar{x}}_{1},\ldots ,{\bar{x}}_{n};\bar{\lambda }),\;\;\;i=1,2,\ldots ,N, \end{array}$$

where $f_{i}$ depends on $\bar{\lambda }$ sufficiently smoothly. The value of $\bar{\lambda }$ can have a profound impact on the dynamical behaviour of equation (2.6). In particular, a *bifurcation* [4] is said to occur in equation (2.6) if, as $\bar{\lambda }$ is changed smoothly through a critical value $\bar{\lambda }={\bar{\lambda }}^{*}$, the resulting long-time behaviour is qualitatively different below and above that critical value.

The example CRN equation (2.1) demonstrates that bifurcations are present even in relatively simple molecular systems. In particular, by writing the difference in rate coefficient as a single parameter $\bar{\lambda }=(\alpha_{2}-\alpha_{1})$, and setting $\alpha_{3}=1$ such that the other coefficients are defined relative to $\alpha_{3}$, the associated RRE equation (2.2) is of the form of equation (2.6). We observe that if $\bar{\lambda }>0$ (i.e. $\alpha_{2}>\alpha_{1}$), then molecular concentrations starting near zero, i.e. with $0<x_{1}(0)\ll 1$, will equilibrate to $\bar{\lambda }$. However, if $\bar{\lambda }<0$ (i.e. $\alpha_{2}<\alpha_{1}$), then molecular concentrations starting near zero will equilibrate to zero. In the terminology of dynamical systems, the equilibrium $x_{1}^{*}=0$ goes from stable to unstable as the parameter $\bar{\lambda }$ changes from negative to positive, while the other equilibrium $x_{1}^{*}=\lambda$ then goes from unstable to stable. This change of stability is an example of a bifurcation; in fact, ODE equation (2.2) is the normal form for *transcritical bifurcation* [4].

## 3. Bifurcations with parametrized RNCRNs

The RNCRN is introduced in [51] to approximate target systems of the form of equation (2.3), with vector fields independent of parameters. In this section, we generalize the RNCRN to approximate parameter-dependent target systems. In particular, let us now consider as input data the target system

(3.1) $$\begin{array}{rlrl} \frac{d{\bar{x}}_{i}}{dt} & =f_{i}({\bar{x}}_{1},\ldots ,{\bar{x}}_{n},{\bar{\lambda }}_{1},\ldots ,{\bar{\lambda }}_{L}),\;\; & & \;\;\;\text{ for }i=1,2,\ldots ,N\\ \text{and}\;\frac{d{\bar{\lambda }}_{l}}{dt} & =0,\;\; & & \;\;\;\text{ for }l=1,2,\ldots ,L, \end{array}$$

whose vector field $f=(f_{1},f_{2},\ldots ,f_{N})$ depends sufficiently smoothly on L positive parameters ${\bar{\lambda }}_{1},{\bar{\lambda }}_{2},\ldots ,{\bar{\lambda }}_{L}>0$, which can be seen as a generalization of equation (2.6); note that, if the parameters are not positive, then one can define new positive parameters by suitably shifting the old ones. In equation (3.1), we append the trivial ODEs for the parameters, allowing one to interpret them as auxiliary dependent variables. Provided that the initial conditions for these auxiliary variables match the target parameter values, the ODEs with parametrized vector fields, and those with vector fields depending on the auxiliary variables subject to trivial ODEs, are equivalent.

**Parametrized RNCRNs.** Let us now present a generalized RNCRN which can approximate the dynamics of the parameter-dependent target systems, represented in the form of equation (3.1), over a range of parameter values. In particular, consider the *parametrized* RNCRN defined via the RREs

(3.2) $$\begin{array}{rlrl} \frac{dx_{i}}{dt} & =\beta_{i}+x_{i}\sum_{j=1}^{M}\alpha_{i,j}y_{j}, & & \;\;\;\text{ for }i=1,\ldots ,N,\\ \frac{d\lambda_{k}}{dt} & =0, & & \;\;\;\text{ for }l=1,\ldots ,L\\ \text{and}\;\frac{dy_{j}}{dt} & =\frac{\gamma_{j}}{\mu }+\left(\sum_{i=1}^{N}\frac{\omega_{j,i}}{\mu }x_{i}+\sum_{l=1}^{L}\frac{\psi_{j,l}}{\mu }\lambda_{l}+\frac{\theta_{j}}{\mu }\right)y_{j}-\frac{1}{\mu }y_{j}^{2}, & & \;\;\;\text{ for }j=1,\ldots ,M, \end{array}$$

where $x_{i}=x_{i}(t)\geq 0$, $y_{j}=y_{j}(t)\geq 0$, $\lambda_{l}=\lambda_{l}(t)=\lambda_{l}(0)\geq 0$, $\beta_{i},\gamma_{j}\geq 0$ and $\alpha_{i,j},\omega_{ji},\theta_{j},\psi_{j,l}\in R$. In particular, compared to the original RNCRN equation (2.4), the parametrized RNCRN equation (3.2) contains additional executive species $\Lambda_{l}$, called *parameter species*, which are static in the sense that they are governed by trivial ODEs $d\lambda_{l}/dt=0$. Let us stress that, by representing parameters as species $\Lambda_{l}$ in equation (3.2), their values are controlled via initial concentrations, as opposed to rate coefficients. Species $\Lambda_{l}$ influence chemical perceptrons $Y_{j}$, but not vice versa, and do so via the following catalytic reactions:

(3.3) $$\begin{array}{rl} & \Lambda_{l}+Y_{j}\overset{|\psi_{j,l}|/\mu \;}{\to }\Lambda_{l}+Y_{j}+\text{sign}(\psi_{j,l})Y_{j},\;\;\;j=1,2,\ldots ,M,\;\;l=1,2,\ldots ,L, \end{array}$$

where, for brevity, $\text{sign}(\psi_{j,l})$ is used to indicate that the products contain two $Y_{j}$ molecules when $\psi_{j,l}$ is positive, and none when it is negative.

The parametrized RNCRN can be trained with a slight modification to algorithm 1 from [51]; for completeness, we present this algorithm for equation (3.2) in appendix D.1.

Since parameters can be represented as dependent variables with trivial dynamics, as in equation (3.2), one may expect that the RNCRN, which can approximate dependent variables undergoing dynamics resulting from arbitrary smooth vector fields [51], can also reproduce some bifurcations. In what follows, we numerically demonstrate via examples that the RNCRN can approximate two classical bifurcations, namely a Hopf bifurcation [4] and a homoclinic bifurcation [44,65]. We note that both of these types of bifurcations have been observed in biochemical contexts [13,14].

### 3.1. Hopf bifurcation

Let us apply RNCRNs to approximate the target system

(3.4) $$\begin{array}{rl} \frac{d{\bar{x}}_{1}}{dt} & =\left({\bar{\lambda }}_{1}-2-({\bar{x}}_{1}-5{)}^{2}-({\bar{x}}_{2}-5{)}^{2}\right)({\bar{x}}_{1}-5)-({\bar{x}}_{2}-5),\\ \frac{d{\bar{x}}_{2}}{dt} & =\left({\bar{\lambda }}_{1}-2-({\bar{x}}_{1}-5{)}^{2}-({\bar{x}}_{2}-5{)}^{2}\right)({\bar{x}}_{2}-5)+({\bar{x}}_{1}-5)\\ \text{and}\;\frac{d{\bar{\lambda }}_{1}}{dt} & =0, \end{array}$$

which undergoes a Hopf bifurcation [4]. In particular, equation (3.4) is obtained from the normal form of a Hopf bifurcation by shifting the target equilibrium to $\left({\bar{x}}_{1},{\bar{x}}_{2})=(5,5\right)$, and the critical parameter value, at which the Hopf bifurcation occurs, to ${\bar{\lambda }}_{1}^{*}=2$, so that the dynamics of interest are within the positive orthant. The $\left({\bar{x}}_{1},{\bar{x}}_{2}\right)$ state space of equation (3.4) is displayed in figure 2a, at and above the critical parameter value. In particular, when ${\bar{\lambda }}_{1}=1<2$, one can notice that the trajectories are attracted to the stable equilibrium $\left({\bar{x}}_{1},{\bar{x}}_{2})=(5,5\right)$, while for ${\bar{\lambda }}_{1}=3>2$, they are attracted to an isolated periodic orbit, called a limit cycle, enclosing the now unstable equilibrium $\left({\bar{x}}_{1},{\bar{x}}_{2})=(5,5\right)$.

**Figure 2.**
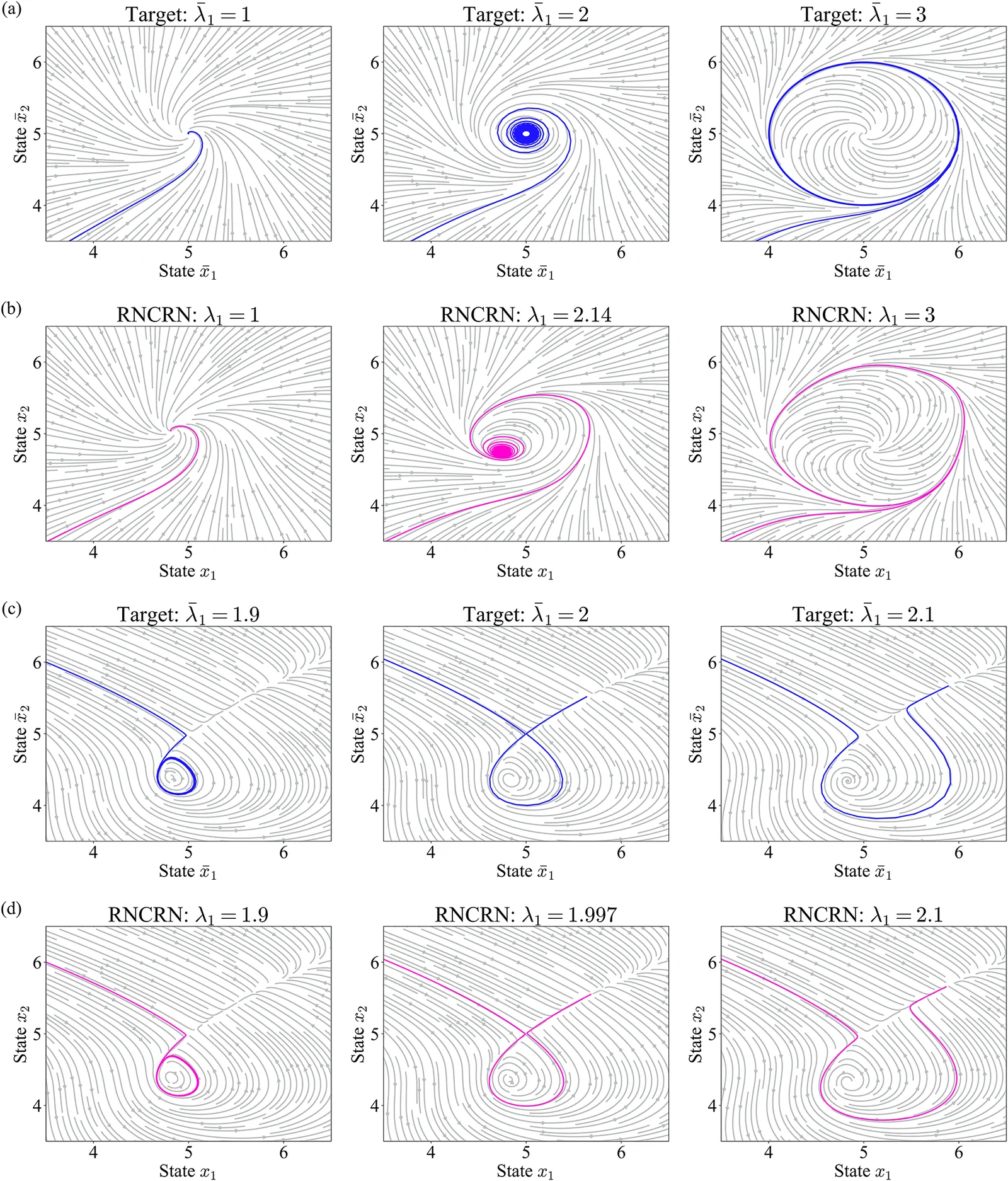
Approximation of two bifurcation-exhibiting ODEs using RNCRNs. Row (a) shows the target ODE equation (3.4) undergoing a Hopf bifurcation as the parameter ${\bar{\lambda }}_{1}$ is varied through its critical value. Row (b) shows the M=5-chemical-perceptron RNCRN approximation from appendix A.1 with μ=0.01 undergoing the Hopf bifurcation as its equivalent control species $\lambda_{1}$ is varied. Row (c) shows the target ODE equation (3.5) undergoing a homoclinic bifurcation as the parameter ${\bar{\lambda }}_{1}$ is varied. Row (d) shows the M=10-chemical-perceptron RNCRN approximation from appendix A.2 with μ=0.0075 undergoing a homoclinic bifurcation. The grey arrows show the vector field of the target ODEs for rows (a,c), whereas for rows (b,d), the arrows show the vector field of the reduced RNCRN (i.e. μ=0).

One way to replicate this behaviour locally in the parameter space is to train the original RNCRN equation (2.4) to approximate equation (3.4) specifically at the bifurcation point $\bar{\lambda }=2$. The results of such a training are presented in appendix A.3, showing that the RNCRN numerically replicates the dynamics of equation (3.4) for parameter values in a small neighbourhood of ${\bar{\lambda }}_{1}^{*}=2$. In particular, even though it is trained only at a single value ${\bar{\lambda }}_{1}^{*}=2$, the RNCRN automatically reproduces the transition from stable equilibrium to stable limit cycle locally in the parameter space.

To replicate the dynamics of equation (3.4) over a larger set in the parameter space, we train the parametrized RNCRN of the form equation (3.2) to approximate equation (3.4) for all $1\leq {\bar{\lambda }}_{1}\leq 3$ with M=5 chemical perceptrons; see appendix A.1 for details. We display the state space of the resulting RNCRN in figure 2b, demonstrating that qualitatively similar dynamics and bifurcation are inherited. Let us note that the RNCRN undergoes the Hopf bifurcation, not necessarily exactly at $\lambda_{1}=2$, but at a slightly different critical value, numerically found to be $\lambda_{1}\approx 2.14$. This difference in the critical parameter values is a consequence of the approximating nature of the RNCRN, and can be reduced if the number and speed of chemical perceptrons are increased. However, in this paper, we focus on the RNCRNs with a smaller number of chemical perceptrons that still produce adequate behaviours, as CRNs with more species are, in general, more challenging to build experimentally.

### 3.2. Homoclinic bifurcation

Let us now consider the target system

(3.5) $$\begin{array}{rl} \frac{d{\bar{x}}_{1}}{dt} & =\left({\bar{\lambda }}_{1}-2-\frac{4}{5}\right)({\bar{x}}_{1}-5)+({\bar{x}}_{2}-5)-\frac{6}{5}({\bar{x}}_{1}-5)({\bar{x}}_{2}-5)+\frac{3}{2}({\bar{x}}_{2}-5{)}^{2},\\ \frac{d{\bar{x}}_{2}}{dt} & =({\bar{x}}_{1}-5)-\frac{4}{5}({\bar{x}}_{2}-5)-\frac{4}{5}({\bar{x}}_{2}-5{)}^{2}\\ \text{and}\;\frac{d{\bar{\lambda }}_{1}}{dt} & =0, \end{array}$$

which undergoes a homoclinic bifurcation [44,65] in the vicinity of the equilibrium $\left({\bar{x}}_{1},{\bar{x}}_{2})=(5,5\right)$ at the critical parameter value ${\bar{\lambda }}_{1}^{*}=2$. The state space of equation (3.5) is displayed in figure 2c, at and above the critical parameter value. In particular, when ${\bar{\lambda }}_{1}=1.9<2$, there is a stable limit cycle in the state space, which is absent when ${\bar{\lambda }}_{1}=2.1>2$. This transition happens at ${\bar{\lambda }}_{1}=2$ via a homoclinic orbit—a solution in the state space that connects the equilibrium $\left({\bar{x}}_{1},{\bar{x}}_{2})=(5,5\right)$ to itself. To replicate these dynamical behaviours, we train the parametrized RNCRN equation (3.2) with M=10 chemical perceptrons to approximate equation (3.4) for all $1.9\leq {\bar{\lambda }}_{1}\leq 2.1$; see appendix A.2 for details. We show in figure 2d that the RNCRN displays the desired dynamics.

Before closing this section, let us note that there are other systematic methods for designing CRNs with bifurcations [43,44], such as Hopf or homoclinic bifurcations, which in general contain a smaller number of species and reactions compared to the RNCRNs. In particular, being based on ANNs, the strength of the RNCRNs is not necessarily in producing relatively small systems, but instead in being more versatile with the types of input data needed to achieve desired objectives. In the next two sections, we further explore this ability.

## 4. Piecewise systems with classification-controlled RNCRNs

In §3, we have trained RNCRNs to exhibit predefined bifurcations by using the parametrized target ODEs of the form of equation (3.1) as input data, which already display the desired bifurcations. Importantly, for classical bifurcations to exist in such ODEs, the vector field from equation (3.1) is required to be sufficiently smooth with respect to the parameters [66]. In this section, we relax this smoothness assumption by allowing the vector field from equation (3.1) to be discontinuous with respect to the parameters. The resulting ODEs are then known as *piecewise systems* [67], and provide more flexible training data for the RNCRN. In particular, on the one hand, this approach may allow one to simply specify suitable dynamics piecewise in the parameter space, thereby implicitly enforcing desired types of bifurcations at the associated boundaries, which the RNCRN is allowed to automatically interpolate. On the other hand, in some applications, more important may be that a system displays certain dynamical features for certain parameters values, rather than how it transitions between the different regimes, and the piecewise approach allows for this without imposing unnecessary regularity conditions on the dynamics. In what follows, we adapt the parametrized RNCRN from §3 to be trainable on such piecewise systems, replicating the desired dynamics for desired parameter values, while automatically connecting the different dynamical behaviours via suitable bifurcations.

### 4.1. Simple unistable–bistable piecewise system

As a motivation, let us consider the target system

(4.1) $$\begin{array}{r} \frac{d{\bar{x}}_{1}}{dt}=f_{1}({\bar{x}}_{1},{\bar{\lambda }}_{1})=\left\{ \begin{array}{clclc} & 30-6{\bar{x}}_{1}, & \; & \text{ if }{\bar{\lambda }}_{1}=1, & \\ & -\left({\bar{x}}_{1}-2\right)({\bar{x}}_{1}-5)({\bar{x}}_{1}-8), & \; & \text{ if }{\bar{\lambda }}_{1}=0. & \end{array}\right. \end{array}$$

In particular, the vector field has a unique stable equilibrium (unistability) when ${\bar{\lambda }}_{1}=1$, while it has two stable equilibria (bistability) when ${\bar{\lambda }}_{1}=0$. Being defined at only two isolated points in the parameter space, equation (4.1) is a piecewise system.

We train the parametrized RNCRN equation (3.2) with three chemical perceptrons to replicate the dynamics of equation (4.1), with details shown in appendix B.1 and the training algorithm in appendix D.3, and with the results presented in figure 3. In particular, in figure 3a–d, we demonstrate that the RNCRN is unistable at $\lambda_{1}=1$ and bistable at $\lambda_{1}=0$, respectively, with the equilibria relatively close to those of equation (4.1).

**Figure 3.**
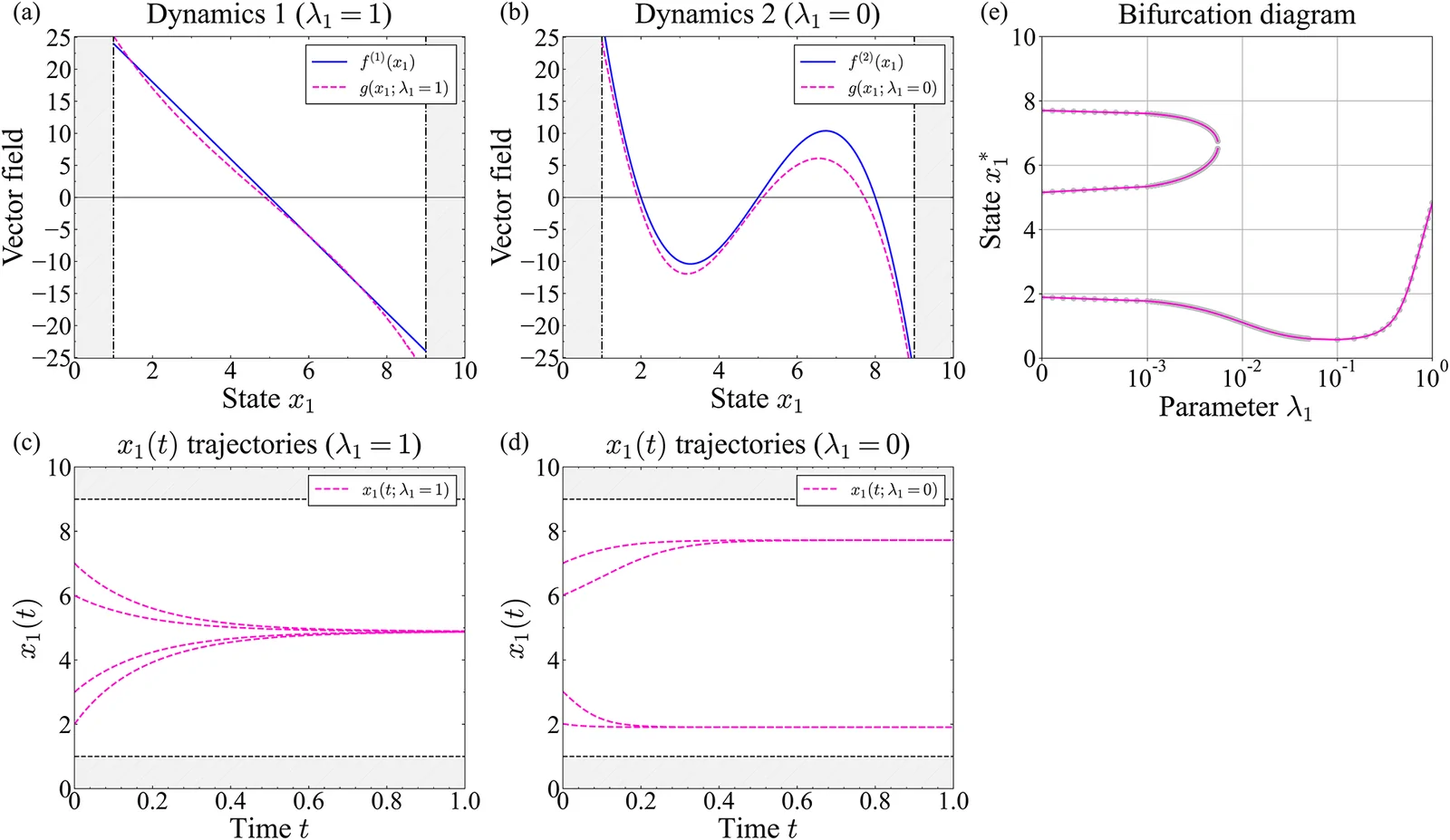
Emergence of a bifurcation via interpolation when an RNCRN is trained at discrete concentrations of a parameter species. Approximation of equation (4.1) by the parametrized RNCRN equation (B 1) specified in appendix B.1. Panel (a) shows the dynamics in the first dynamical regime ($\lambda_{1}=1$). The target dynamics $\text{d}{\bar{x}}_{1}/\text{d}t=30-6{\bar{x}}_{1}$ are shown in blue, while the reduced vector field of the RNCRN approximation, i.e. when μ=0, are shown in magenta. Panel (b) shows the dynamics in the second dynamical regime ($\lambda_{1}=0$). In blue, the target dynamics $\text{d}{\bar{x}}_{1}/\text{d}t=-\left({\bar{x}}_{1}-2\right)({\bar{x}}_{1}-5)({\bar{x}}_{1}-8)$, while in magenta, the reduced vector field of the RNCRN approximation. Panels (c,d) show $x_{1}(t)$ time trajectories over a variety of executive species initial conditions, $x_{1}(0)=2,3,6,7$, for the full RNCRN in each dynamical regime with μ=0.1 and all chemical perceptron initial conditions at zero $y_{1}(0)=y_{2}(0)=y_{3}(0)=0$. Panel (e) shows the bifurcation diagram for the RNCRN approximation as the parameter $\lambda_{1}$ is varied through intermediate values in magenta. A bifurcation is observed at around $\lambda_{1}=10^{-2}$.

As opposed to the target system equation (4.1), which is defined only at ${\bar{\lambda }}_{1}=1$ and ${\bar{\lambda }}_{1}=0$, the RNCRN is defined for all $\lambda_{1}\geq 0$, and depends smoothly on this parameter. To analyse this dependence, we display a bifurcation diagram in figure 3e—the equilibria of the RNCRN as a function of the parameter $\lambda_{1}$. One can notice that the RNCRN automatically interpolates unistability at $\lambda_{1}=1$ and bistability at $\lambda_{1}=0$ via a classical bifurcation. In particular, one of the equilibria exists for all the parameter values shown in figure 3e, while the other two exist for all $\lambda_{1}\in [0,\lambda_{1}^{*})$ for some $\lambda_{1}^{*}<10^{-2}$, after which they disappear via saddle-node bifurcation [4].

### 4.2. Classification-controlled RNCRNs

Let us now consider a general target piecewise system taking the form

(4.2) $$\begin{array}{r} \frac{d{\bar{x}}_{i}}{dt}=f_{i}^{\left(p\right)}({\bar{x}}_{1},\ldots ,{\bar{x}}_{N})\;\text{for all}\;\bar{\lambda }\in L_{p},\;i=1,\ldots ,N,\;p=1,\ldots ,P, \end{array}$$

where $\bar{\lambda }=({\bar{\lambda }}_{1},{\bar{\lambda }}_{2},\ldots ,{\bar{\lambda }}_{L})\in R^{L}$ is the vector of parameters, $L_{1},L_{2},\ldots ,L_{P}\subset R^{L}$ are suitable disjoint sets in the parameter space and $f_{i}^{\left(p\right)}$ is a suitably smooth function. In particular, when the parameters $\bar{\lambda }$ are chosen to lie in the set $L_{p}$, the ODE is governed by the vector field $f^{\left(p\right)}$. Let us note that set $L_{p}$ can be a single point. For example, system equation (4.1) is a special case of equation (4.2) with a single parameter ${\bar{\lambda }}_{1}$, and two single-point parameter regions $L_{1}=\{1\}$ and $L_{2}=\{0\}$.

The parametrized RNCRN equation (3.2) was sufficient to approximate piecewise system equation (4.1), which is defined at isolated parameter values. In order to approximate more general piecewise systems equation (4.2), which have more complicated parameter-space structure, we now modify the RNCRN. In particular, consider the parametrized RNCRN with RREs

(4.3) $$\begin{array}{rlrl} \frac{dx_{i}}{dt} & =\beta_{i}+x_{i}\sum_{j=1}^{M}\alpha_{i,j}y_{j}, & & \;\;\;i=1,2,\ldots ,N\\ \text{and}\;\frac{dy_{j}}{dt} & =\frac{\gamma_{j}}{\mu }+y_{j}\left(\sum_{i=1}^{N}\frac{\omega_{j,i}^{\left(y\right)}}{\mu }x_{i}+\frac{\theta_{j}^{\left(y\right)}}{\mu }+\frac{\psi_{j}}{\mu }r\right)-\frac{\tau_{j}}{\mu }y_{j}^{2}, & & \;\;\;j=1,2,\ldots ,M, \end{array}$$

which are of the form of equation (3.2) with a single parameter r, which itself is governed by the RREs

(4.4) $$\begin{array}{rlrl} \frac{d\lambda_{l}}{dt} & =0, & & \;\;\;l=1,2,\ldots ,L,\\ \frac{dz_{k}}{dt} & =\frac{\gamma }{\mu }+z_{k}\left(\sum_{l=1}^{L}\frac{\omega_{k,l}^{\left(z\right)}}{\mu }\lambda_{l}+\frac{\theta_{k}^{\left(z\right)}}{\mu }\right)-\frac{1}{\mu }z_{k}^{2}, & & \;\;\;k=1,2,\ldots ,K\\ \text{and}\;\frac{dr}{dt} & =\frac{\gamma }{\mu }+r\left(\sum_{k=1}^{K}\frac{\omega_{k}^{\left(r\right)}}{\mu }z_{k}+\frac{\theta_{0}^{\left(r\right)}}{\mu }\right)-\frac{1}{\mu }r^{2}, \end{array}$$

which involve auxiliary chemical perceptrons with concentrations $z_{1},z_{2},\ldots ,z_{K}$. We call the RNCRN corresponding to the RREs equation (4.3) and equation (4.4) the *classification-controlled* RNCRN, and illustrate it in figure 4. Let us note that this RNCRN, like the original equation (2.4), contains only two timescales: the slow species $X_{i}$ and $\Lambda_{l}$, and the fast species $Y_{j}$, $Z_{k}$ and R.

**Figure 4.**
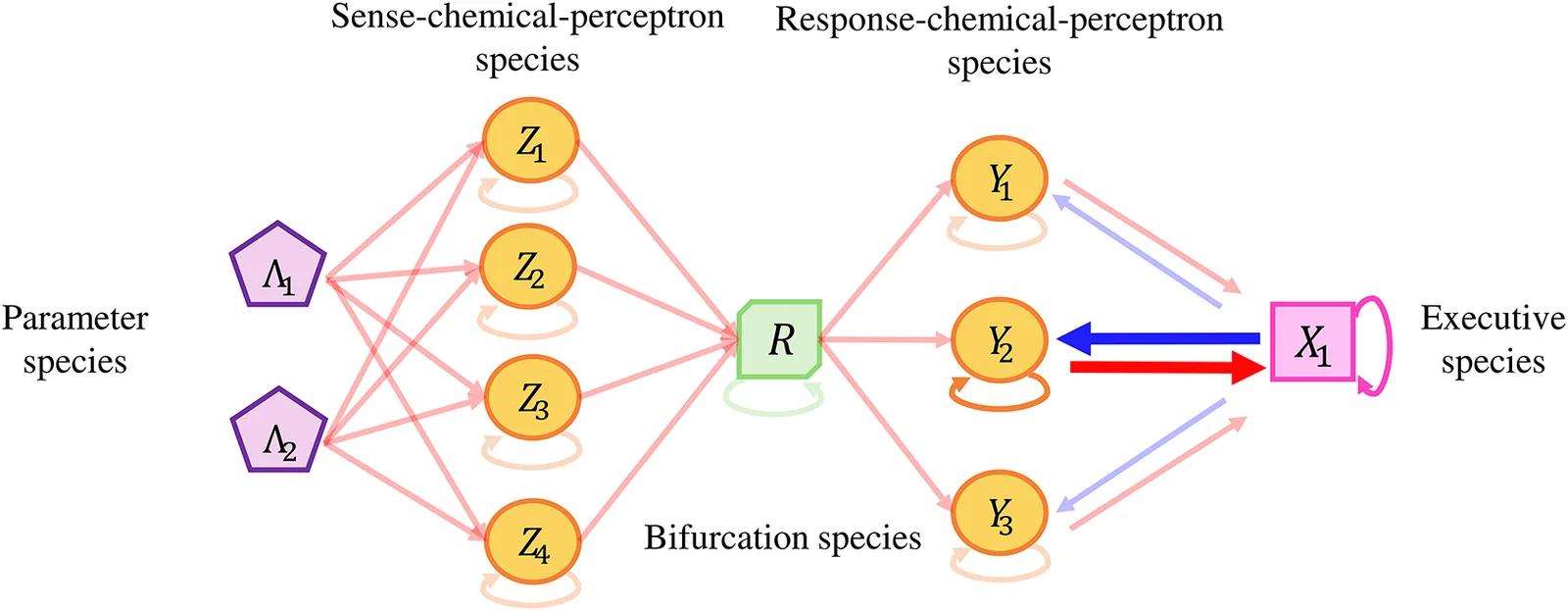
A schematic of the classification-controlled RNCRN used to approximate equation (4.6). In purple, the parameter species $\Lambda_{1}$ and $\Lambda_{2}$ are shown to interact with the sense-chemical-perceptron species that perform the nonlinear classification presenting the output as the magnitude of the bifurcation species R. The bifurcation species R then acts as a parameter in the response-chemical-perceptron species that are trained to induce the intended dynamics in the executive species.

Loosely, equation (4.3) is designed to implement the dynamics of equation (4.2) pointwise, while equation (4.4) then assembles/classifies this dynamics to the appropriate parts of the parameter space. In particular, in the parametrized RNCRN equation (3.2), the ODEs for the chemical perceptron depend linearly on the parameters $\lambda_{l}$, while the perceptrons in equation (4.3) depend on $\lambda_{l}$ only implicitly via the auxiliary variable r. Importantly, it follows from equation (4.4) that, for every sufficiently small μ>0,

(4.5) $$\begin{array}{rl} z_{k} & \approx \sigma_{\gamma }\left(\sum_{l=1}^{L}\omega_{k,l}^{\left(z\right)}\lambda_{l}(0)+\theta_{k}^{\left(z\right)}\right)\;\;\text{and}\;\;r\approx \sigma_{\gamma }\left(\sum_{k=1}^{K}\omega_{k}^{\left(r\right)}z_{k}+\theta_{0}^{\left(r\right)}\right), \end{array}$$

where $\sigma_{\gamma }(x)$ is the activation function defined in equation (2.5). Equation (4.5) therefore operates as a two-layer ANN, with a single perceptron in the second layer, that maps between parameters λ and the pointwise control parameter r.

Based on this modular design, we train the classification-controlled RNCRN in two steps. In the first step, we disregard the precise structure of the parameter space, and simply train the parametrized RNCRN equation (4.3) pointwise to display at some $r=r_{p}$ the dynamical behaviour induced by the vector field $f^{\left(p\right)}$ for every p=1,2,…,P. Then, in the second step, we factor in the precise structure of the parameter space by training equation (4.4) to map from a given $L_{p}$ set in $\left(\lambda_{1},\lambda_{2},\ldots ,\lambda_{L}\right)$-space to the associated $r_{p}$ value by using standard feed-forward ANN training methods via equation (4.5). We present an algorithm for this training in appendix D.4.

### 4.3. XOR-gated unistable–bistable piecewise system

Let us now consider a target piecewise system with more complicated parameter-space structure than equation (4.1), depending on two parameters ${\bar{\lambda }}_{1}\text{ and }{\bar{\lambda }}_{2}$, and given by

(4.6) $$\begin{array}{r} \frac{d{\bar{x}}_{1}}{dt}=\left\{ \begin{array}{clclc} & 30-6{\bar{x}}_{1}, & \; & \text{ for all }({\bar{\lambda }}_{1},{\bar{\lambda }}_{2})\in L_{1}=\left(K_{1}\times K_{2}\right)\cup \left(K_{2}\times K_{1}\right), & \\ & -\left({\bar{x}}_{1}-2\right)({\bar{x}}_{1}-5)({\bar{x}}_{1}-8), & \; & \text{ for all }({\bar{\lambda }}_{1},{\bar{\lambda }}_{2})\in L_{2}=\left(K_{1}\times K_{1}\right)\cup \left(K_{2}\times K_{2}\right), & \end{array}\right. \end{array}$$

where $K_{1}=[0,1)$ and $K_{2}=[1,2]$. Loosely, if on the domain [0,2]×[0,2], one of the parameters is in the larger region, while the other is in the smaller region, then the piecewise system equation (4.6) is unistable; otherwise, it is bistable. This dependence can be interpreted as an XOR operator, and creates in the parameter space a nonlinear boundary between different dynamical regions.

To approximate the dynamics of equation (4.6), we use algorithm 4 to train the classification-controlled RNCRN equation (4.3) and equation (4.4), with details presented in appendix B.2. In particular, in the first step of the algorithm, we simply re-use the parametrized RNCRN from appendix B.1, which was previously constructed in §4.1 to approximate system equation (4.1). In the second step, we then train the classification module equation (4.4) to implement the parameter-space structure from equation (4.6).

**Algorithm 2. Algorithm 1 from [51] is modified to include static executive species (i.e. parameter species). In this modified training procedure, $\lambda_{1},\ldots ,\lambda_{L}$ and $\psi_{j,l}$ are treated as additional inputs to the ANN component of the algorithm in the same way as $x_{1},\ldots ,x_{M}$ and $\omega_{j,i}$, and then one minimizes over the $x_{1},\ldots ,x_{M}$ the target functions as usual. This is equivalent to removing any feedback to $\lambda_{l}$ and so setting $d\lambda_{l}/dt=0$. See the accompanying code examples [76] for more details.**

**Figure:**
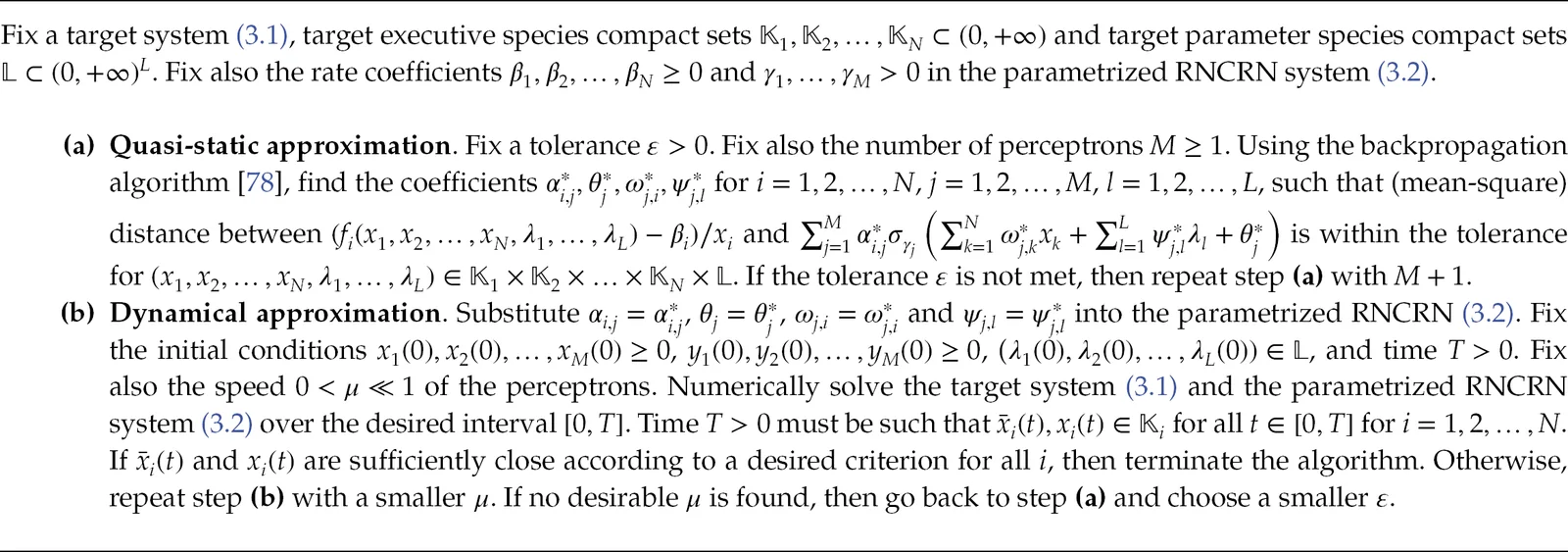

**Algorithm 3. Two-step algorithm for training the *parametrized RNCRN* to approximate piecewise systems. See the accompanying code examples [76] for more details.**

**Figure:**
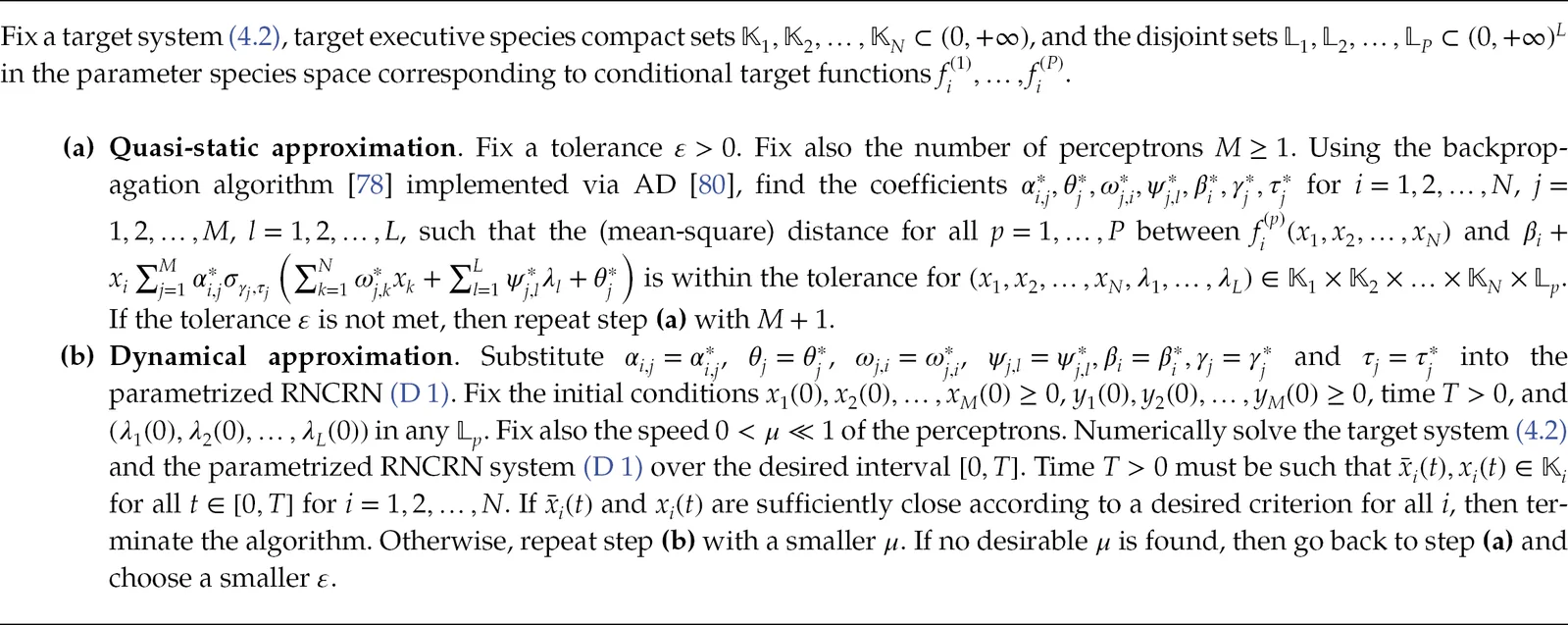

**Algorithm 4. An algorithm to train *classification-controlled RNCRN* to approximate piecewise systems of the form (4.2). See the accompanying code examples [76] for more details.**

**Figure:**
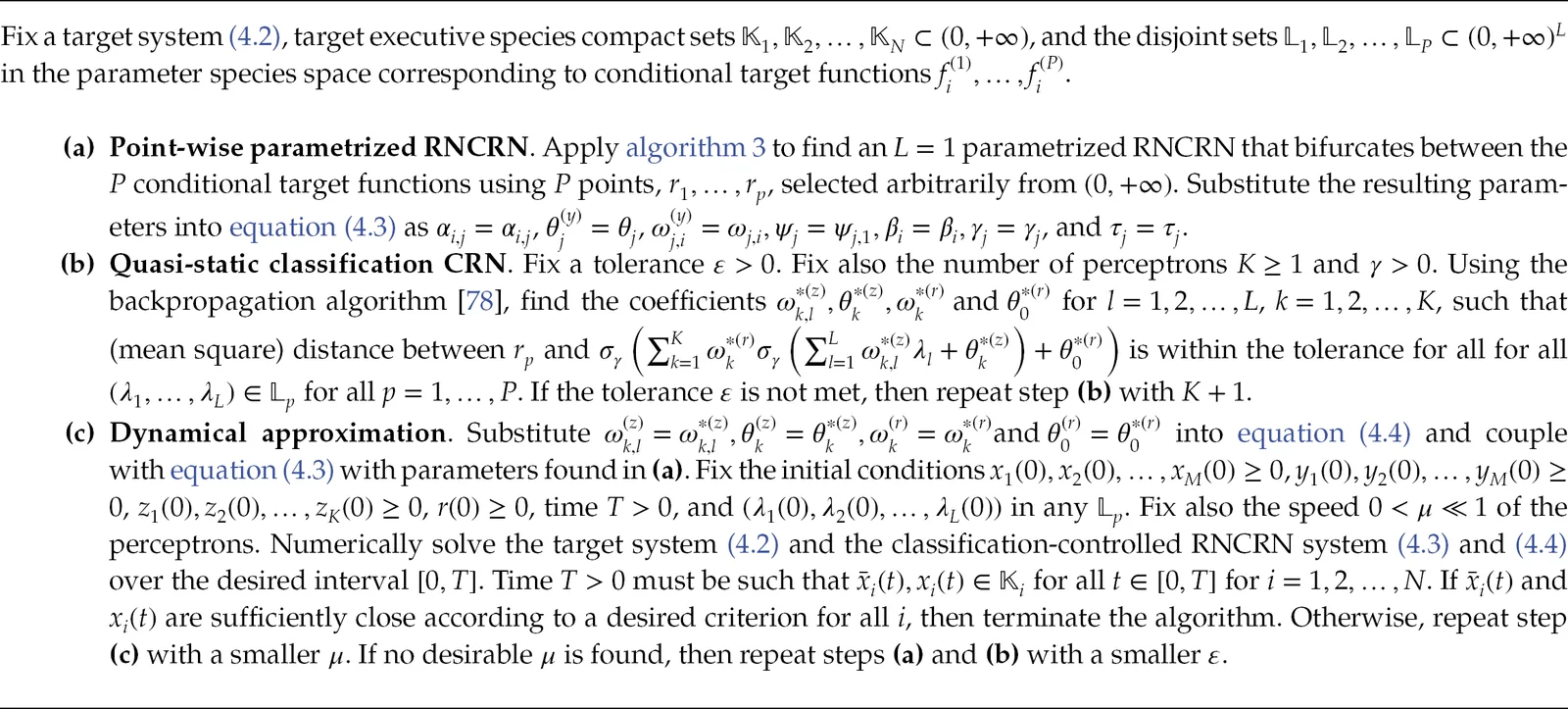

In figure 5, we plot a two-parameter bifurcation diagram for the resulting RNCRN, displaying representative trajectories $x_{1}(t)$ at different points in the $\left(\lambda_{1},\lambda_{2}\right)$ parameter space. One can notice a relatively good match with the target piecewise system equation (4.6): the RNCRN is unistable approximately when $\left(\lambda_{1},\lambda_{2})\in ([0,1.0]\times [1.1,2.0])\cup ([1.1,2.0]\times [0,1.0]\right)$ and bistable when $\left(\lambda_{1},\lambda_{2})\in ([0,1.0]\times [0,1.0])\cup ([1.1,2.0]\times [1.1,2.0]\right)$. Furthermore, the RNCRN depends smoothly on the parameters $\left(\lambda_{1},\lambda_{2}\right)$, and interpolates the dynamics across the boundaries of the parameter space via saddle-node bifurcation (as shown in figure 5d).

**Figure 5.**
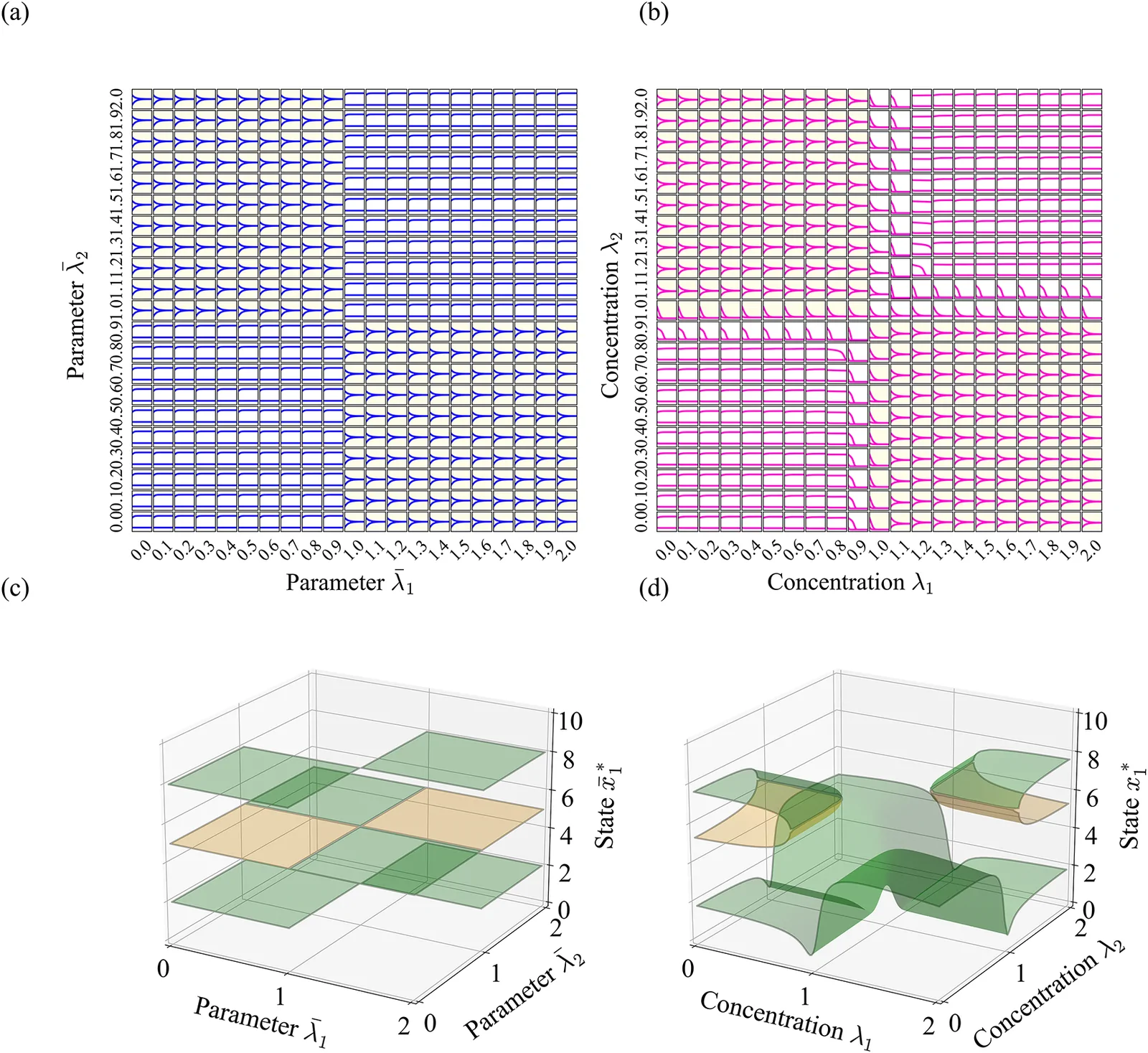
Exploration of the bifurcation boundary separating bistability and unistability in the RNCRN approximation of the nonlinear XOR classification system. Panel (a) shows $x_{1}(t)$ trajectories for different concentrations of $\Lambda_{1}$ and $\Lambda_{2}$ for the target piecewise system in equation (4.6), whereas panel (b) shows the $x_{1}(t)$ trajectories for the associated classification-controlled RNCRN, i.e. equations (4.3) and (4.4). The RNCRN uses four sensing chemical perceptrons, one bifurcation chemical perceptron and three response chemical perceptrons to induce the intended dynamics in a single executive species according to the states of two parameter species, $\Lambda_{1}$ and $\Lambda_{2}$. Each sub-panel contains two time trajectories simulated over a total time of t=1 with μ=0.001. All initial concentrations are set to zero, except for the executive species, which is initialized as $x_{1}(0)=2$ and $x_{1}(0)=7$ for the two trajectories. The hue of each sub-panel indicates the intended dynamical behaviour: yellow corresponds to unistable behaviour, while white corresponds to bistable behaviour. Panels (c,d) show a two-parameter bifurcation diagram for the target system and the RNCRN approximation, respectively. The coloured surfaces represent the equilibrium values of $X_{1}$ as the parameter species values are varied; stable equilibria are shown in green, while unstable equilibria are shown in orange. More details are given in appendix B.2.

In the XOR system, boundary points such as $\lambda_{1}=1$ and $\lambda_{2}=1$ could be assigned arbitrarily to either regime. However, we note that, in equation (4.6), we use semi-open intervals, so this point is assigned to the regime defined by $L_{2}$. If, instead, equation (4.6) were defined using open intervals, i.e. $K_{1}=(0,1)$ and $K_{2}=(1,2)$, we could still discretize these intervals and apply the training algorithm. As such, the inclusion or exclusion of boundary points in training would depend on the discretization procedure. For this example, the allocation of boundary points will have only a minor impact on the quality of the RNCRN approximation, as the location of the boundary is imprecise, with the bistable regime not being triggered for parameter values more than 0.1 away from the intended values.

## 5. Data-defined limit cycles and switching

In both §§3 and 4, the RNCRNs are trained on the data taking the form of ODEs with suitably regular vector fields. In this section, we exploit the interpolation and extrapolation abilities of the underlying neural network-based methodology to show that the RNCRN can also be trained in an ODE-free manner from relatively sparse data: a set of points in the state space. More precisely, we focus on generating the data that may allow the RNCRN to incorporate into its state space two-dimensional limit cycles defined in an ODE-free manner.

To this end, we sample a finite number of points on, and in a neighbourhood of, a given closed curve in the state space. We then assign a single vector to each of these points with appropriate direction: the vectors on the closed curve point along it (corresponding to the limit cycle), while the remaining vectors all point either towards the closed curve or away from it (corresponding to respectively stability or instability). One thus obtains a local vector field, defined only on a finite number of states, which can be used to train the RNCRN, allowing it to extrapolate the behaviour beyond the grid. The algorithm to generate these data is given as algorithm 1. Let us note that the vector field near the limit cycle varies exponentially with states in the algorithm; in appendix C.3, we show that a linear variation can also be used. Let us also note that this algorithm can be generalized to higher dimensions; see appendix C.6 for a three-dimensional version and an example.

### 5.1. Circular limit cycle

Consider a circle of radius a>0 centre at $(b_{1},b_{2})\in R_{>}^{2}$, with parametric equation given by

(5.1) $$\begin{array}{rl} x_{1}(s) & =a\cos(s)+b_{1}\\ \text{and}\;x_{2}(s) & =a\sin(s)+b_{2}, \end{array}$$

where s∈[0,2π). In what follows, we choose a=1 and $\left(b_{1},b_{2})=(2,2\right)$. We sample an ordered set of points $P_{d}=(x_{1}(s_{d}),x_{2}(s_{d}))$ by taking $s=s_{d}=0.01(d-1)$ in equation (5.1) for d=1,…,629630. Using algorithm 1 with K=20, δ=0.01, κ=1 and η=1, we generate the input data to train the RNCRN equation (2.4) with M=5 chemical perceptrons to display in the state space a stable two-dimensional limit cycle which is close to the circle. This training is performed by suitably adapting algorithm 1 from [51], as presented in algorithm 5 and appendix C.1; see also appendix C.2 for an example RNCRN with approximately circular limit cycle which is unstable.

**Algorithm 5. An algorithm for training an RNCRN to approximate dynamics from vector field data. Termination of this algorithm is based on an informal inspection of the time trajectories as there are no ground-truth time trajectories for these systems. See the accompanying code examples [76] for more details.**

**Figure:**
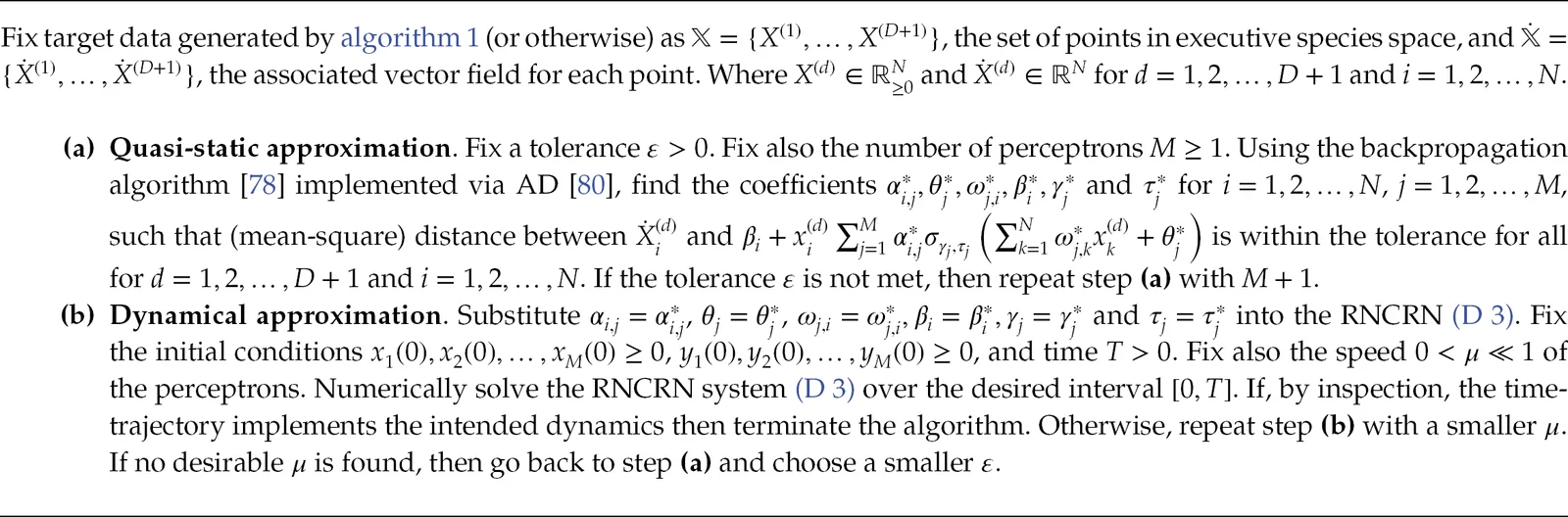

We show the training data, together with the state space of the trained RNCRN, in figure 6a. One can notice that the RNCRN with μ=0.1 numerically displays a stable limit cycle whose shape is relatively close to the desired circle. Furthermore, the RNCRN extends the vector consistently beyond this limit cycle, producing inside it an unstable equilibrium.

**Figure 6.**
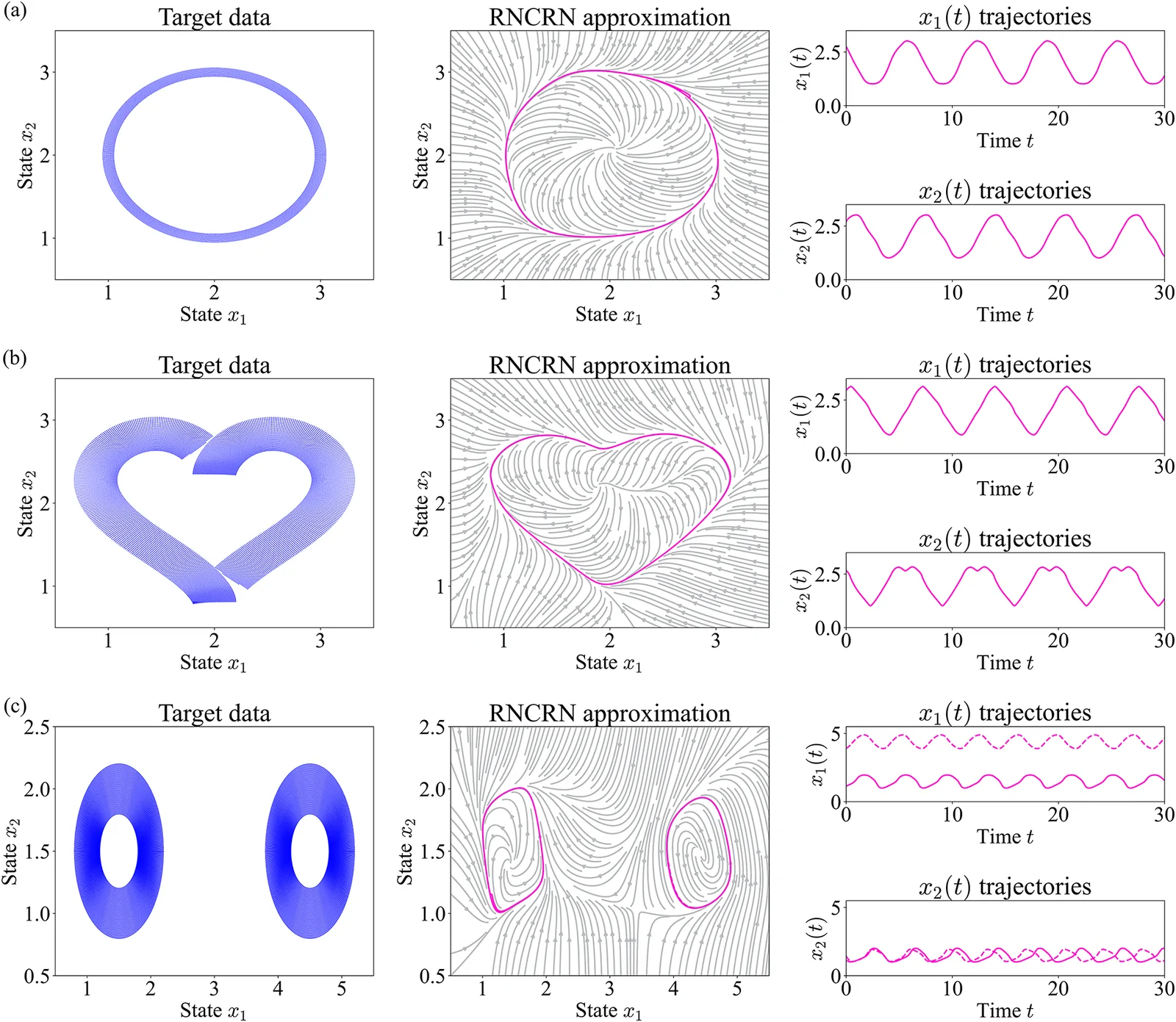
Example RNCRNs trained on data-defined limit cycles. Column 1 shows the points generated by algorithm 1 at which the training data are given for different target attractor shapes. A close-up of the training data is shown in appendix C. Column 2 shows the RNCRN approximation trained on the data in column 1. The phase plane of the reduced RNCRN approximations, i.e. the RNCRN with μ=0, is shown as grey arrows, while example ($x_{1},x_{2}$)-trajectories of the full RNCRNs with μ>0 are shown in magenta. Column 3 shows time trajectories of the full RNCRN approximation. Row (a) shows a five-chemical-perceptron RNCRN (full parameters are given in appendix C.1) used to approximate a data-defined circular limit cycle. The time trajectory was simulated with $x_{1}(0)=2.744,x_{2}(0)=2.693$, μ=0.1 and $y_{1}(0)=\ldots ,y_{5}(0)=0$. Row (b) shows a 10-chemical-perceptron RNCRN (as specified in appendix C.4) approximating the data-defined heart-shaped limit cycle. The time trajectory was simulated with $x_{1}(0)=2.957,x_{2}(0)=2.665$, μ=0.1 and $y_{1}(0)=\ldots ,y_{10}(0)=0$. Row (c) shows a 15-chemical-perceptron RNCRN (specified in appendix C.5) approximating a system with two limit cycles. The two time trajectories were both simulated with μ=0.1 and $y_{1}(0)=\ldots ,y_{15}(0)=0$, the executive species’ initial concentrations differed with $x_{1}(0)=1.118,x_{2}(0)=1.154$ or $x_{1}(0)=3.916,x_{2}(0)=1.392$.

### 5.2. Heart-shaped limit cycle

Let us now consider the parametric equation

(5.2) $$\begin{array}{rl} x_{1}(s) & =2+1.12\sin(s{)}^{3}\\ \text{and}\;x_{2}(s) & =2+0.91\cos(s)-0.35\cos(2s)-0.14\cos(3s)-0.07\cos(4s), \end{array}$$

which for s∈[0,2π) produces a heart shape in the $\left(x_{1},x_{2}\right)$-space. In this example, we generate the training data for each lobe of the heart separately, $s_{d}=0.01(d-1)$ for d=1,2,…,261262, and d=314315…,575576, and then use algorithm 1 with K=20, δ=0.01, κ=1 and η=1; see appendix C.4 for more details. We display the results in figure 6b, numerically demonstrating that the RNCRN displays a stable limit cycle that is approximately heart-shaped and, consistently with the given local vector field, creates an unstable equilibrium inside the cycle.

### 5.3. Multiple limit cycles

Algorithm 1 can also be used to train RNCRNs to display multiple coexisting limit cycles. For example, let us apply algorithm 1 to generate data for two coexisting stable limit cycles of the form of equation (5.1), one with a=0.5 and $\left(b_{1},b_{2})=(1.5,1.5\right)$, and the other with a=0.5 and $\left(b_{1},b_{2})=(4.5,1.5\right)$, as described in detail in appendix C.5. The result of training an RNCRN on the concatenated training data is shown in figure 6c, numerically demonstrating two stable limit cycles. Let us note that, in this example, the RNCRN not only generates an unstable equilibrium inside each of the limit cycles, but also creates an equilibrium of saddle type as a way to connect the two regions specified by the training data.

### 5.4. Equilibrium-oscillation piecewise system with data-defined oscillations

Let us now combine the results from this section and §4 by considering the target piecewise system

(5.3) $$\begin{array}{r} \frac{dx}{dt}=\left\{ \begin{array}{clclc} & f^{1}(x), & \; & \text{ for all }{\bar{\lambda }}_{1}\in [0,2.5], & \\ & f^{2}(x), & \; & \text{ for all }{\bar{\lambda }}_{1}>2.5, & \end{array}\right. \end{array}$$

with $x\in R^{2}$, where $f^{1}(x)\in R^{2}$ is a smooth vector field with a unique stable equilibrium at (0.5,0.5), while $f^{2}(x)$ is a vector field defined only on a finite number of states in $R_{>}^{2}$ via algorithm 1 to display a circular limit cycle. We might imagine chemical systems that implement dynamics such as equation (5.3) are implementing environment-specific oscillations. That is, when an environmental signal, $\Lambda_{1}$, is abundant, the system will produce oscillations of an executive species, $X_{1}$, but when the environmental signal is weak, trace amounts of the executive species will be produced.

We now consider using a classification-controlled RNCRN, equations (4.3) and (4.4), to implement equation (5.3). Following the steps in algorithm 4, modified as in algorithm 5 to use the data-defined vector field, we train a classification-controlled RNCRN to implement equation (5.3); see appendix C.7 for details (5 $Y_{j}$-chemical perceptrons, 3 $Z_{k}$-chemical perceptrons and a single bifurcation species was used).

Figure 7a,b shows the data-defined oscillations and the stable equilibrium as blue arrows. The grey streamlines in figure 7a,b show the vector field of the reduced parametrized RNCRN for two particular values of the bifurcation species, R=0.1 and R=1, which were used in training. The input–output relationship between the parameter species $\Lambda_{1}$ and the bifurcation species R is shown in figure 7c.

**Figure 7.**
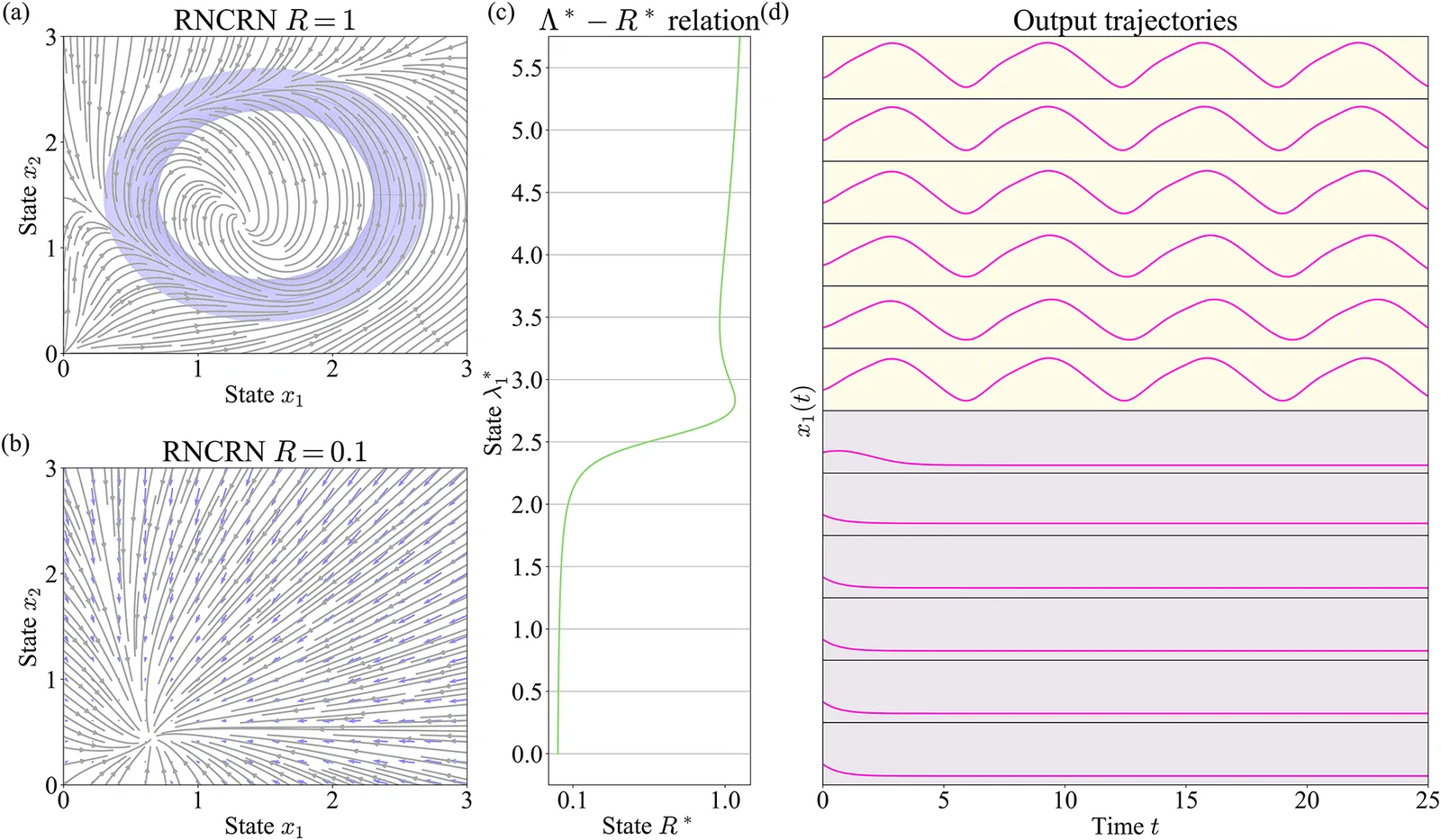
A classification-controlled RNCRN designed to approximate a piecewise system that produces either trace amounts of executive species $X_{1}$ or oscillations in $X_{1}$ according to the concentration of environmental species $\Lambda_{1}$. Panels (a,b) show the data-defined target dynamics (in blue) as well as the reduced vector field of the point-defined RNCRN approximation as grey arrows. Panel (c) shows the input–output relationship between $\Lambda_{1}$ and R (an intermediary species). Panel (d) shows numerically integrated time trajectories of output species, $x_{1}(t)$, for the full RRE of the classification-controlled RNCRN with different $\lambda_{1}(0)$ values (according to the alignment with panel (c) $\lambda_{1}^{*}$-axis) and μ=0.1. Full details of the reactions in this system are given in appendix C.7.

To show the dependence of the $X_{1}$ behaviour on the parameter species $\Lambda_{1}$, sub-panels in figure 7d have been aligned with the $\lambda_{1}^{*}$-axis in figure 7c. Each sub-panel in figure 7d corresponds to a different $\lambda_{1}(0)$ concentration and plots the resulting $x_{1}(t)$ time trajectories. The full classification-controlled RNCRN is defined in appendix C.7, and we simulate it using μ=0.01 and all chemical-perceptron species concentrations starting from zero. We observe that the classification-controlled RNCRN produces the desired behaviour. Below a critical value of $\lambda_{1}\approx 2.5$, we observe that the $x_{1}(t)$ concentrations tend to a low equilibrium (the magenta panels in figure 7d), but above that point, we observe oscillations of a similar frequency and amplitude (the yellow panels in figure 7d). This demonstrates an effective approximation of piecewise systems as different regions of $\lambda_{1}$-space are being mapped to different functions in $\left(x_{1},x_{2}\right)$-space (and these functions are largely independent of $\lambda_{1}$ in these regions).

## 6. Discussion

In this work, we use an RNCRN architecture to approximate atypical dynamical behaviours and demonstrate that different dynamical regimes can be instantiated in a single system, along with bifurcations as the system moves between those regimes. Mapping target dynamical behaviours, in the form of a non-chemical target ODE, into a CRN has an extensive literature [31,43–50]. These predominately analytical techniques can produce small CRNs in terms of reaction and species number. For example, the target ODE with a homoclinic bifurcation considered here (equation (3.5)) was realized with only two chemical species and nine chemical reactions [44] compared to the 12 chemical species and 82 chemical reactions required by the RNCRN approximation identified here. Nonetheless, showing that RNCRNs can accurately replicate these bifurcations served as a motivation for the later sections where such analytical mapping techniques do not apply.

Sections 4 and 5 demonstrate the major advantage of the RNCRN over the aforementioned analytical techniques: RNCRNs are mathematically derived from ANNs which are well known for their ability to learn functions via data-driven training techniques. Exploiting this fact, we introduced algorithm 1 for generating artificial data to train an RNCRN to reproduce isolated dynamical features defined on small regions of phase space. We were able to use algorithm 1 to define exotic attractor shapes including a heart-shaped limit cycle, multiple limit cycles and a three executive species toroidal limit cycle (see appendix C.6).

A key feature of RNCRN-based methods was the ability of the RNCRN to extrapolate and interpolate dynamical behaviour to achieve dynamics in the whole space consistent with the features of interest. For example, we observe the presence of multiple equilibria that were not provided in the training data in the reduced vector fields of figure 6. Similarly, bifurcations naturally emerge—without their location or type being specified explicitly—when RNCRNs are made to interpolate between qualitatively distinct behaviours at different values of the input parameters species. This ability for the RNCRN to fill in the gaps of the vector field makes it much easier to design complicated dynamics as there is no requirement to find an ODE with such dynamics.

In §5.4 we presented a classification-controlled RNCRN inspired by applications where it is necessary to sense the chemical environmental signals and act accordingly—in this case, turn on an oscillating output. Several major challenges with modern therapeutics could be addressed by systems with similar behaviour. Most straightforwardly, environmental-specific activation would allow for the localization of therapeutic activity within pathologically relevant environments thus reducing off-target effects. It is well known, for example, that off-target killing of benign cells limits the success of chemotherapies [68] and antibiotic therapies [69]. More interestingly for our purposes, switching to a new dynamical regime would allow fine-tuned control over the temporal concentration of a therapeutic species and would allow for the design of non-trivial dosing profiles. For example, it has been suggested that pulsatile dosing schedules are beneficial for preventing the development of resistance in a wide range of contexts including antibiotic resistance [70], cancer treatments [71] and preventing insulin resistance in diabetes treatments [72]. It should be noted that experimental chemical neural networks have already been built that are able to classify RNAs from cancer [73] and bacterial infections [23], highlighting that classification-controlled RNCRNs might one day be developed in such contexts.

There are several outstanding challenges that remain to be explored in the context of RNCRNs. While the underlying reactions can, in principle, be encoded using DNA strand displacement [51], it is possible that other contexts such enzyme-aided DNA nanotechnology [74] or transcription factor networks [75] may be more convenient in practice. In that vein, the principles behind the construction of the RNCRN are more general than the particular CRNs used to instantiate the chemical perceptron. We anticipate that many alternative CRNs that play the equivalent role, i.e. weighted summation and nonlinear activation, might be engineered to instantiate RNCRN-like systems in a range of biochemical media. We would like to explore systematic compilation methods that take the RNCRN’s CRN and convert it into a dynamically equivalent reaction network with alternative kinetic laws (such as enzyme-based synthetic biology). An open question is whether interesting RNCRNs are feasible in practice. The number of reacting components in the RNCRNs presented in this work is lower than in some other complex engineered molecular networks [21,22], but a feature of RNCRNs that is more challenging to engineer is the necessary degree of connectivity between components and control over kinetics. We would therefore like to investigate compression or pruning algorithms to systematically reduce the number of auxiliary chemical species and chemical reactions within the resulting CRNs.

## Figures and Tables

**Figure 8.**
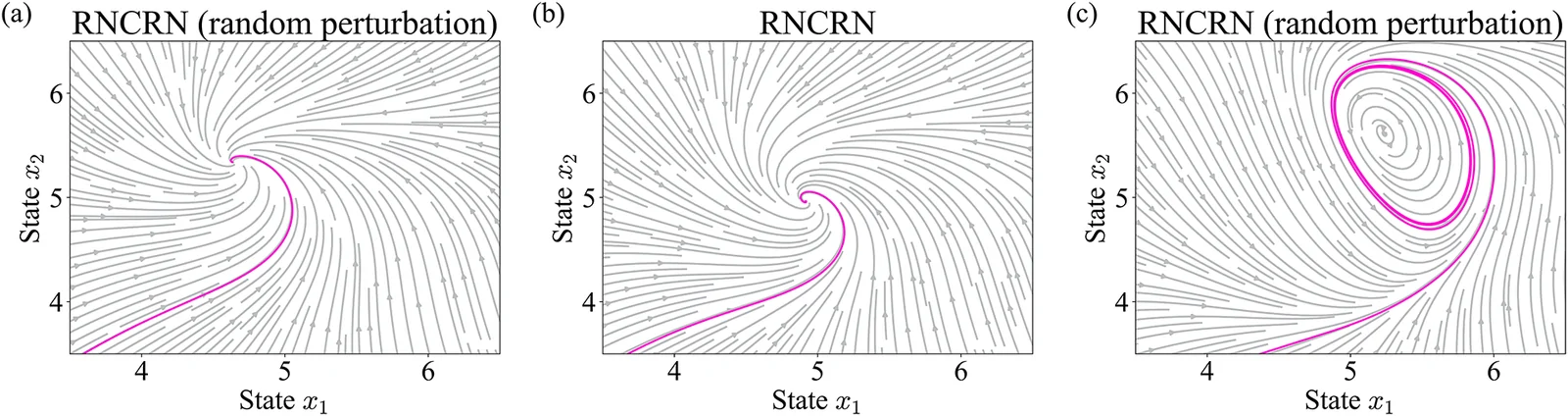
An RNCRN trained at the critical point of a Hopf bifurcation, i.e. equation (A 7), demonstrating extrapolation of dynamics to behaviours outside of the training data. Panel (b) shows the M=6-chemical-perceptron RNCRN trained to approximate equation (A 7), while panels (a,c) show the same RNCRN approximation with the rates randomly perturbed (see appendix A.3 for the rates of the RNCRNs). All panels show their respective reduced RNCRN vector field as grey arrows and $\left(x_{1}(t),x_{2}(t)\right)$-time trajectories with μ=0.01, and initial concentrations $x_{1}(0)=x_{2}(0)=2$ and $y_{1}(0)=\cdots =y_{6}(0)=0$ in magenta.

**Figure 9.**
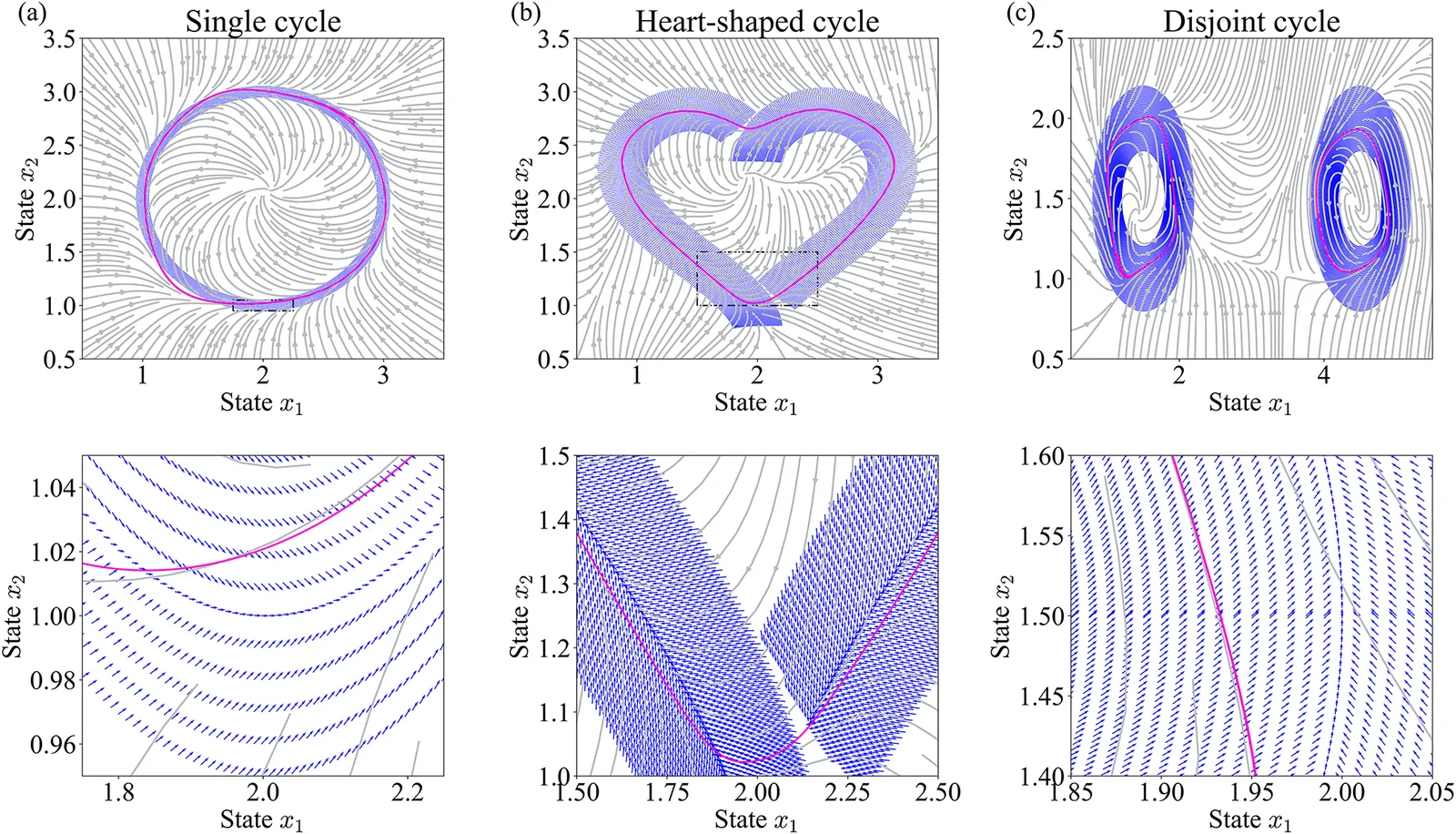
Artificial data, shown in blue, are used to train the data-defined attractors plotted in figure 6. The reduced RNCRN approximations, i.e. the RNCRN with μ=0, are shown as grey arrows, while example ($x_{1},x_{2}$)-trajectories of the full RNCRNs with μ>0 are shown in magenta. Panels (a–c) show the results for the single circular cycle (§5.1), the heart-shaped cycle (§5.2), and the two disjoint cycles (§5.3), respectively.

**Figure 10.**
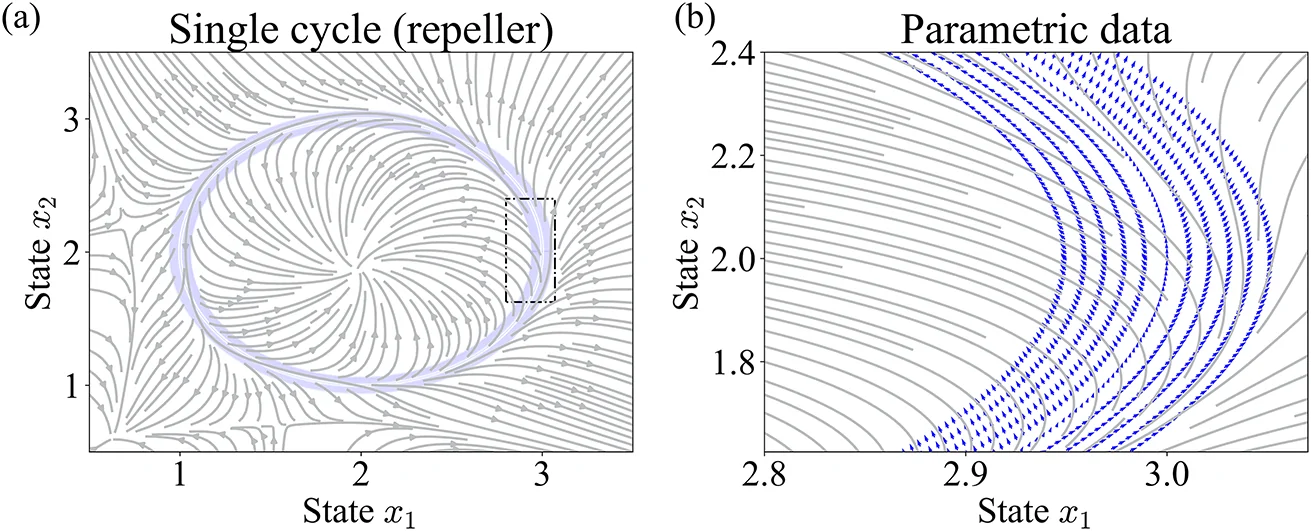
Example RNCRN trained on data to realize a circular repeller. In blue, the target repeller data that were created by sampling points from parametric equation (5.1) then applying a vector field interpolation algorithm, i.e. algorithm 1. In grey, the reduced RNCRN vector field for a five-chemical-perceptron RNCRN (see appendix C.2 for parameters). Panel (a) shows the entire state space of interest, whereas panel (b) shows a close-up of the state space to emphasize the training data.

**Figure 11.**
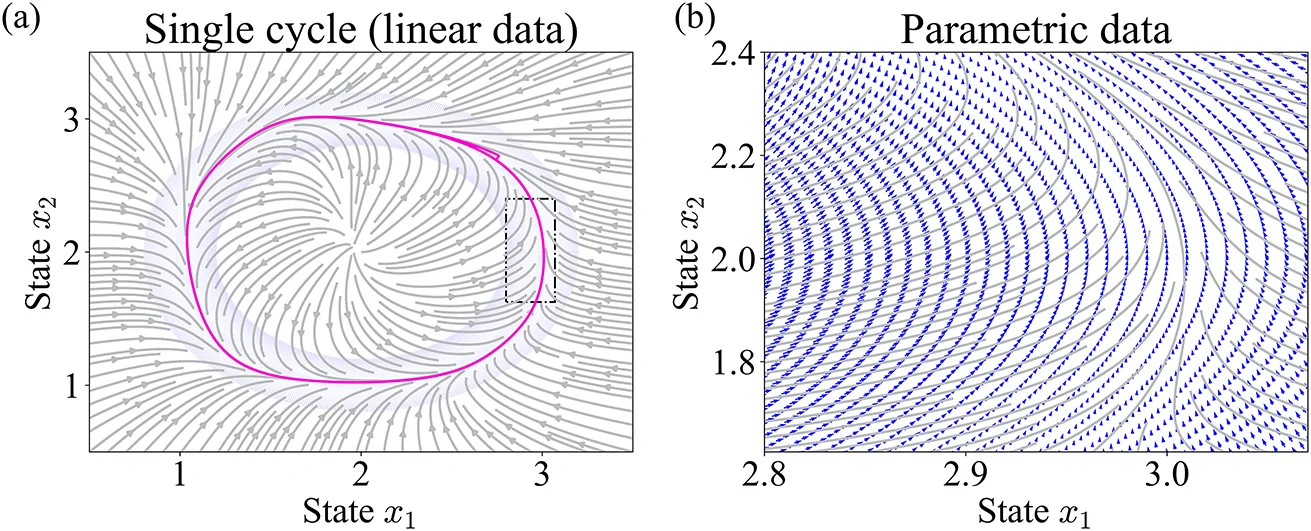
Example RNCRN trained on data to realize a circular attractor using a linear modification of algorithm 1. In blue, the target attractor data were created by sampling points from parametric equation (5.1) then applying a vector field interpolation algorithm, i.e. algorithm 1, with a linear modification. In grey, the reduced RNCRN vector field for a five-chemical-perceptron RNCRN (see appendix C.3 for parameters). Panel (a) shows the entire state space of interest, whereas panel (b) shows a close-up of the state space to emphasize the training data. The time trajectory was simulated with $x_{1}(0)=2.744,x_{2}(0)=2.693$, μ=0.1 and $y_{1}(0)=\ldots ,y_{5}(0)=0$.

**Figure 12.**
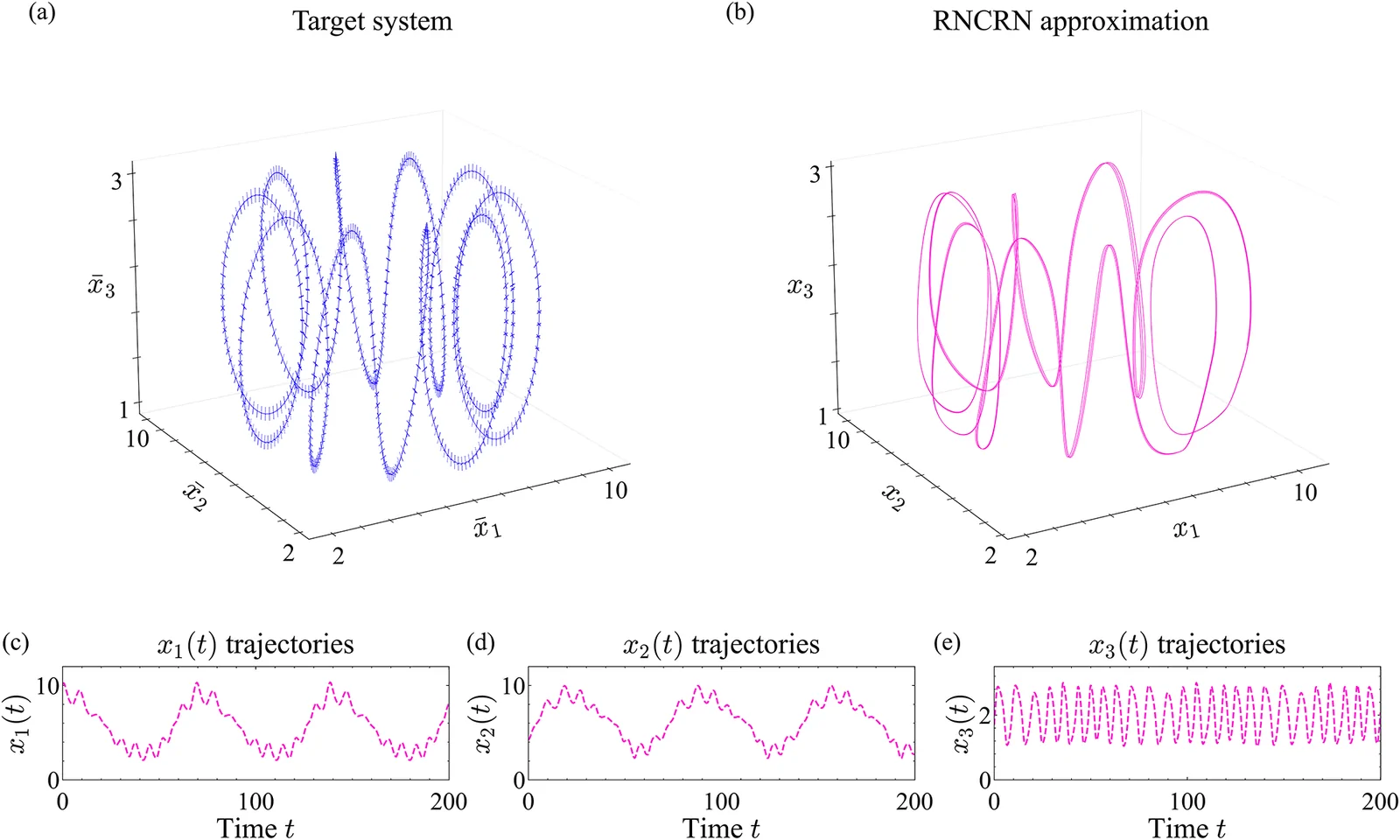
An RNCRN trained to realize a toroidal attractor without an underlying ODE. Panel (a) shows the target attractor data, in blue, that were created by sampling points from parametric equation (C 16) then applying a modified vector field interpolation algorithm, i.e. algorithm 1. Panel (b) shows, in magenta, an example state space trajectory from the M=7 RNCRN approximation with μ=0.01, $x_{1}(0)=10.126$, $x_{2}(0)=4.257$, $x_{3}(0)=1.703$ and $y_{1}(0)=\ldots ,y_{7}(0)=0$ (the system is fully specified in appendix C.6). Panels (c–e) show the same trajectory as in panel (b) but in the time domain for $x_{1}(t),x_{2}(t)$ and $x_{3}(t)$, respectively.

**Figure:**
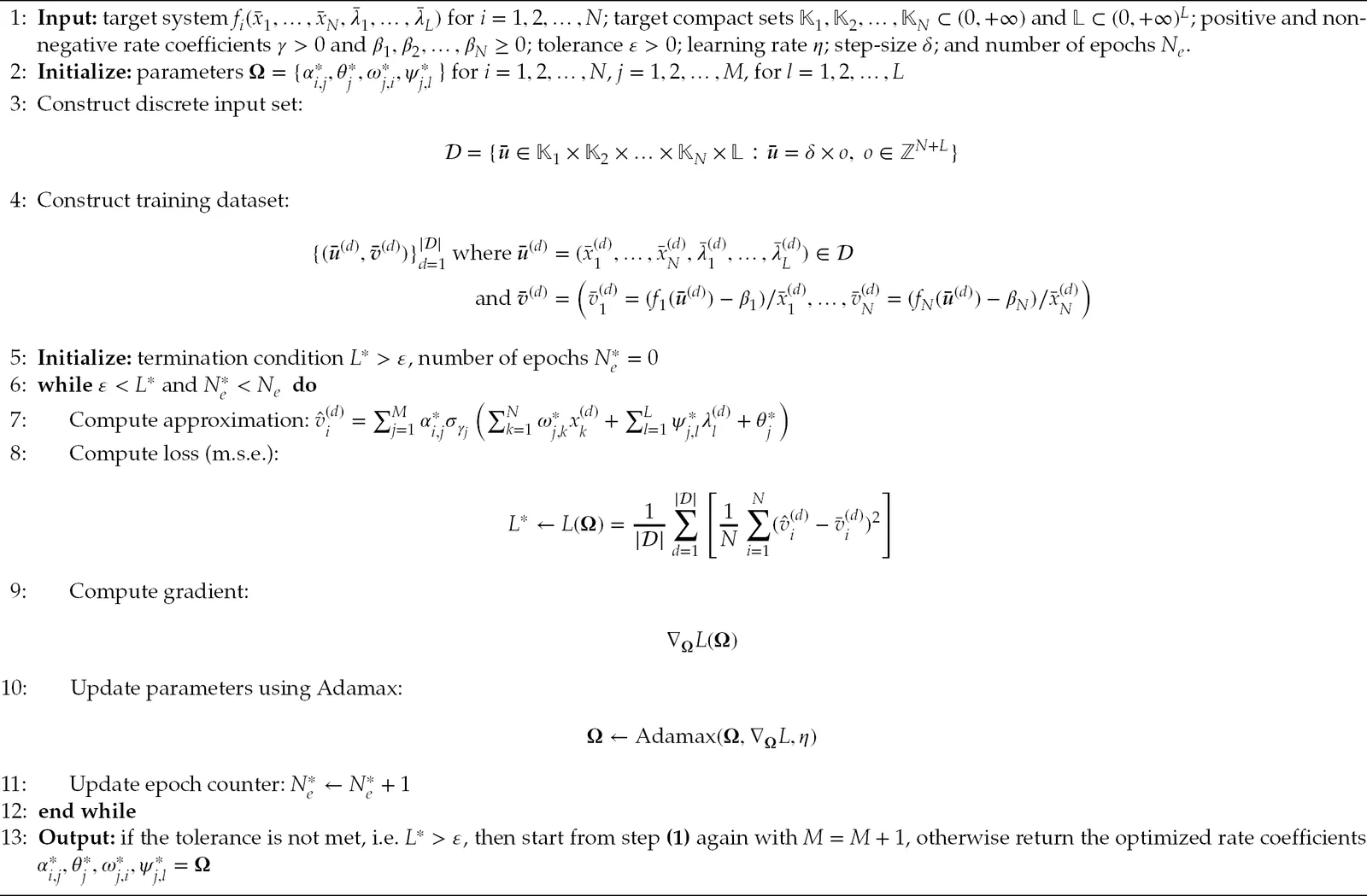


## Data Availability

The code is available as an archived GitHub repository on Zenodo [76]. All codes used to generate figures and examples are included in this repository. The data not included in GitHub are available on Zenodo [77].

## Appendix A. Example bifurcations

### A.1. Hopf bifurcation

We use algorithm 2, a modified version of algorithm 1 from [51] with static executive species, to approximate the target system equation (3.4) on $K_{1}=[3.5,6.5],K_{2}=[3.5,6.5]$ and L=[1,3] with a parametrized RNCRN. The absolute tolerance $\varepsilon \approx 10^{-1}$ is met with an RNCRN with M=5 chemical perceptrons and coefficients $\beta_{1}=\beta_{2}=260$, γ=1, and

(A 1) $$\begin{array}{rl} \alpha_{1} & =\left(\begin{array}{c} -4.315\\ -5.861\\ -9.788\\ 3.796\\ -1.209 \end{array}\right),\;\;\alpha_{2}=\left(\begin{array}{c} -1.505\\ -4.968\\ 0.149\\ -25.501\\ 0.824 \end{array}\right),\;\;\theta =\left(\begin{array}{c} 10.656\\ 8.520\\ 5.385\\ 3.369\\ 4.653 \end{array}\right),\;\;\\ \omega_{1} & =\left(\begin{array}{c} -1.298\\ -0.228\\ -1.545\\ 0.103\\ -2.239 \end{array}\right),\;\;\omega_{2}=\left(\begin{array}{c} -0.599\\ -0.131\\ 0.061\\ -0.940\\ 1.676 \end{array}\right)\text{and }\psi =\left(\begin{array}{c} 0.105\\ -0.080\\ 0.070\\ 0.039\\ 0.069 \end{array}\right), \end{array}$$

where $\alpha_{i}=(\alpha_{i,1},\alpha_{i,2},\ldots ,\alpha_{i,5}{)}^{⊤}$ for i=1,2, $\omega_{i}=(\omega_{1,i},\omega_{2,i},\ldots ,\omega_{5,i}{)}^{⊤}$ for i=1,2, $\theta =(\theta_{1},\theta_{2},\ldots ,\theta_{5}{)}^{⊤}$ and $\psi =(\psi_{1},\psi_{2},\ldots ,\psi_{5}{)}^{⊤}$. The reduced ODEs are given by

(A 2) $$\begin{array}{rl} \frac{d{\tilde{x}}_{1}}{dt} & =g_{1}({\tilde{x}}_{1},{\tilde{x}}_{2};\lambda_{1})=260+{\tilde{x}}_{1}\sum_{j=1}^{5}\alpha_{1,j}\sigma_{1}\left(\omega_{j,1}{\tilde{x}}_{1}+\omega_{j,2}{\tilde{x}}_{2}+\psi_{j}\lambda_{1}+\theta_{j}\right)\\ \text{and}\;\frac{d{\tilde{x}}_{2}}{dt} & =g_{2}({\tilde{x}}_{1},{\tilde{x}}_{2};\lambda_{1})=260+{\tilde{x}}_{2}\sum_{j=1}^{5}\alpha_{2,j}\sigma_{1}\left(\omega_{j,1}{\tilde{x}}_{1}+\omega_{j,2}{\tilde{x}}_{2}+\psi_{j}\lambda_{1}+\theta_{j}\right), \end{array}$$

whereas the full ODEs read

(A 3) $$\begin{array}{rlrl} \frac{dx_{1}}{dt} & =260+x_{1}\left(\sum_{j=1}^{5}\alpha_{1,j}y_{j}\right),\;\; & & \;\;\;\;\frac{dx_{2}}{dt}=260+x_{2}\left(\sum_{j=1}^{5}\alpha_{2,j}y_{j}\right),\\ \mu \frac{dy_{1}}{dt} & =1+\left(\sum_{i=1}^{2}\omega_{1,i}x_{i}+\psi_{1}\lambda_{1}+\theta_{1}\right)y_{1}-y_{1}^{2},\;\; & & \;\;\;\;\mu \frac{dy_{2}}{dt}=1+\left(\sum_{i=1}^{2}\omega_{2,i}x_{i}+\psi_{2}\lambda_{1}+\theta_{2}\right)y_{2}-y_{2}^{2},\\ \mu \frac{dy_{3}}{dt} & =1+\left(\sum_{i=1}^{2}\omega_{3,i}x_{i}+\psi_{3}\lambda_{1}+\theta_{3}\right)y_{3}-y_{3}^{2},\;\; & & \;\;\;\;\mu \frac{dy_{4}}{dt}=1+\left(\sum_{i=1}^{2}\omega_{4,i}x_{i}+\psi_{4}\lambda_{1}+\theta_{4}\right)y_{4}-y_{4}^{2},\\ \mu \frac{dy_{5}}{dt} & =1+\left(\sum_{i=1}^{2}\omega_{5,i}x_{i}+\psi_{5}\lambda_{1}+\theta_{5}\right)y_{5}-y_{5}^{2}\;\text{and}\;\; & & \;\;\;\;\frac{d\lambda_{1}}{dt}=0. \end{array}$$

Here, we assume general initial concentrations: $x_{1}(0)=a_{1}\in [3.5,6.5]$ and $x_{2}(0)=a_{2}\in [3.5,6.5]$, and $y_{1}(0)=b_{1}\geq 0$, $y_{2}(0)=b_{2}\geq 0$, $y_{3}(0)=b_{3}\geq 0$, $y_{4}(0)=b_{4}\geq 0$ and $y_{5}(0)=b_{5}\geq 0$. The coefficients were rounded to three decimal places to quote in the text, and the full precision coefficients are available in the code repository.

In figure 2, we used a range of initial conditions for the executive species, but the chemical-perceptron species always started at zero. For figure 2a, we used ${\bar{x}}_{1}(0)=2\text{ and }{\bar{x}}_{2}(0)=2$, and for the RNCRN approximation in figure 2b, we used $x_{1}(0)=2$ and $x_{2}(0)=2$.

### A.2. Homoclinic bifurcation

We use algorithm 2, a modified version of algorithm 1 from [51] with static executive species, to approximate the target system equation (3.5) on $K_{1}=[3.5,6.5],K_{2}=[3.5,6.5]$ and L=[1.9,2.1] with a parametrized RNCRN. Tolerance $\varepsilon \approx 10^{-3}$ is met with an RNCRN with M=10 chemical perceptrons and coefficients $\beta_{1}=\beta_{2}=1$, γ=1, and

(A 4) $$\begin{array}{rl} \alpha_{1} & =\left(\begin{array}{c} 0.307\\ -0.533\\ 0.324\\ 0.999\\ 4.348\\ 1.820\\ -2.823\\ -0.744\\ -1.270\\ 0.837 \end{array}\right),\;\;\alpha_{2}=\left(\begin{array}{c} 0.812\\ 0.923\\ -0.425\\ -0.260\\ -0.191\\ -2.821\\ -0.174\\ 0.228\\ -0.349\\ -0.636 \end{array}\right),\;\;\theta =\left(\begin{array}{c} 0.790\\ 1.455\\ -1.391\\ 3.440\\ -1.901\\ 3.461\\ 0.657\\ 0.634\\ 1.932\\ -3.335 \end{array}\right),\;\;\\ \omega_{1} & =\left(\begin{array}{c} 0.372\\ -0.183\\ -0.205\\ 0.448\\ -0.986\\ -0.056\\ -0.763\\ 1.043\\ -0.253\\ 0.271 \end{array}\right),\;\;\omega_{2}=\left(\begin{array}{c} -0.697\\ 0.084\\ 0.835\\ -1.034\\ 0.797\\ -1.153\\ 0.025\\ -0.515\\ -0.296\\ 0.335 \end{array}\right)\text{ and }\psi =\left(\begin{array}{c} 0.364\\ -0.022\\ -0.781\\ -0.085\\ 0.062\\ -0.023\\ 0.586\\ -0.556\\ 0.323\\ 0.385 \end{array}\right), \end{array}$$

where $\alpha_{i}=(\alpha_{i,1},\alpha_{i,2},\ldots ,\alpha_{i,10}{)}^{⊤}$ for i=1,2, $\omega_{i}=(\omega_{1,i},\omega_{2,i},\ldots ,\omega_{10,i}{)}^{⊤}$ for i=1,2, $\theta =(\theta_{1},\theta_{2},\ldots ,\theta_{10}{)}^{⊤}$ and $\psi =(\psi_{1},\psi_{2},\ldots ,\psi_{10}{)}^{⊤}$. The reduced ODEs are given by

(A 5) $$\begin{array}{rl} \frac{d{\tilde{x}}_{1}}{dt} & =g_{1}({\tilde{x}}_{1},{\tilde{x}}_{2};\lambda_{1})=1+{\tilde{x}}_{1}\sum_{j=1}^{10}\alpha_{1,j}\sigma_{1}\left(\omega_{j,1}{\tilde{x}}_{1}+\omega_{j,2}{\tilde{x}}_{2}+\psi_{j}\lambda_{1}+\theta_{j}\right)\\ \text{and}\;\frac{d{\tilde{x}}_{2}}{dt} & =g_{2}({\tilde{x}}_{1},{\tilde{x}}_{2};\lambda_{1})=1+{\tilde{x}}_{2}\sum_{j=1}^{10}\alpha_{2,j}\sigma_{1}\left(\omega_{j,1}{\tilde{x}}_{1}+\omega_{j,2}{\tilde{x}}_{2}+\psi_{j}\lambda_{1}+\theta_{j}\right), \end{array}$$

whereas the full ODEs read

(A 6) $$\begin{array}{rlrl} \frac{dx_{1}}{dt} & =1+x_{1}\left(\sum_{j=1}^{10}\alpha_{1,j}y_{j}\right),\;\; & & \;\;\;\frac{dx_{2}}{dt}=1+x_{2}\left(\sum_{j=1}^{10}\alpha_{2,j}y_{j}\right),\\ \mu \frac{dy_{1}}{dt} & =1+\left(\sum_{i=1}^{2}\omega_{1,i}x_{i}+\psi_{1}\lambda_{1}+\theta_{1}\right)y_{1}-y_{1}^{2},\;\; & & \;\;\;\mu \frac{dy_{2}}{dt}=1+\left(\sum_{i=1}^{2}\omega_{2,i}x_{i}+\psi_{2}\lambda_{1}+\theta_{2}\right)y_{2}-y_{2}^{2},\\ \mu \frac{dy_{3}}{dt} & =1+\left(\sum_{i=1}^{2}\omega_{3,i}x_{i}+\psi_{3}\lambda_{1}+\theta_{3}\right)y_{3}-y_{3}^{2},\;\; & & \;\;\;\mu \frac{dy_{4}}{dt}=1+\left(\sum_{i=1}^{2}\omega_{4,i}x_{i}+\psi_{4}\lambda_{1}+\theta_{4}\right)y_{4}-y_{4}^{2},\\ \mu \frac{dy_{5}}{dt} & =1+\left(\sum_{i=1}^{2}\omega_{5,i}x_{i}+\psi_{5}\lambda_{1}+\theta_{5}\right)y_{5}-y_{5}^{2},\;\; & & \;\;\;\mu \frac{dy_{6}}{dt}=1+\left(\sum_{i=1}^{2}\omega_{6,i}x_{i}+\psi_{6}\lambda_{1}+\theta_{6}\right)y_{6}-y_{6}^{2},\\ \mu \frac{dy_{7}}{dt} & =1+\left(\sum_{i=1}^{2}\omega_{7,i}x_{i}+\psi_{7}\lambda_{1}+\theta_{7}\right)y_{7}-y_{7}^{2},\;\; & & \;\;\;\mu \frac{dy_{8}}{dt}=1+\left(\sum_{i=1}^{2}\omega_{8,i}x_{i}+\psi_{8}\lambda_{1}+\theta_{8}\right)y_{8}-y_{8}^{2},\\ \mu \frac{dy_{9}}{dt} & =1+\left(\sum_{i=1}^{2}\omega_{9,i}x_{i}+\psi_{9}\lambda_{1}+\theta_{9}\right)y_{9}-y_{9}^{2},\;\; & & \;\;\;\mu \frac{dy_{10}}{dt}=1+\left(\sum_{i=1}^{2}\omega_{10,i}x_{i}+\psi_{10}\lambda_{1}+\theta_{10}\right)y_{10}-y_{10}^{2}\\ \text{and}\;\frac{d\lambda_{1}}{dt} & =0. \end{array}$$

Here, we assume general initial concentrations: $x_{1}(0)=a_{1}\in [3,7]$ and $x_{2}(0)=a_{2}\in [3,7]$, and $y_{1}(0)=b_{1}\geq 0$, $y_{2}(0)=b_{2}\geq 0$, $y_{3}(0)=b_{3}\geq 0$, $y_{4}(0)=b_{4}\geq 0$, $y_{5}(0)=b_{5}\geq 0$, $y_{6}(0)=b_{6}\geq 0$, $y_{7}(0)=b_{7}\geq 0$, $y_{8}(0)=b_{8}\geq 0$, $y_{9}(0)=b_{9}\geq 0$ and $y_{10}(0)=b_{10}\geq 0$. The coefficients were rounded to three decimal places to quote in the text, and the full precision coefficients are available in the code repository. A lower absolute tolerance was required in this example as the homoclinic orbit is a delicate dynamical behaviour to approximate effectively.

In figure 2, we used a range of initial conditions for the executive species, but the chemical-perceptron species always started at zero. For the homoclinic bifurcation, we used slightly different executive species concentrations owing to the sensitivity of the homoclinic orbit. In figure 2c, we used: ${\bar{x}}_{1}(0)=3.5$, ${\bar{x}}_{2}(0)=6.0$ for the sub-panel with ${\bar{\lambda }}_{1}=1.9$; and ${\bar{x}}_{1}(0)=3.5$, ${\bar{x}}_{2}(0)=6.0435$ for the sub-panel with ${\bar{\lambda }}_{1}=2$ and ${\bar{\lambda }}_{1}=2.1$. In figure 2d, we used: $x_{1}(0)=3.5$, $x_{2}(0)=6.0$ for the sub-panel with $\lambda_{1}=1.9$; $x_{1}(0)=3.5$, $x_{2}(0)=6.04$ for the sub-panel with $\lambda_{1}=1.997$; $x_{1}(0)=3.5$, $x_{2}(0)=6.0435$ for the sub-panel with $\lambda_{1}=2.1$.

### A.3. Hopf bifurcation by training at criticality alone

We now demonstrate that by training an RNCRN at the critical point of a Hopf bifurcation, it is possible to find random perturbation of the RNCRN’s parameters that recreate the qualitative behaviour of a Hopf bifurcation without any data about either side of the bifurcation being used during training.

Consider the target ODE for a Hopf bifurcation at its critical point with an equilibrium at $\left(x_{1},x_{2})=(5,5\right)$

(A 7) $$\begin{array}{rl} \frac{d{\bar{x}}_{1}}{dt} & =(-({\bar{x}}_{1}-5{)}^{2}-({\bar{x}}_{2}-5{)}^{2})({\bar{x}}_{1}-5)-({\bar{x}}_{2}-5)\\ \text{and}\;\frac{d{\bar{x}}_{2}}{dt} & =(-({\bar{x}}_{1}-5{)}^{2}-({\bar{x}}_{2}-5{)}^{2})({\bar{x}}_{2}-5)+({\bar{x}}_{1}-5). \end{array}$$

In figure 2a, the $\bar{\lambda }=2$ plot visualizes the dynamics of equation (A 7). We train an RNCRN to approximate equation (A 7) and show the associated vector field of the reduced RNCRN and an example $\left(x_{1}(t),x_{2}(t)\right)$ trajectory in figure 8b. In figure 8a,c, we show that by inducing a particular but randomly sampled perturbation of the rate constants, we can induce unistable or oscillatory behaviour. Unlike the parametrized RNCRN, the training of an RNCRN at criticality alone does not use data about bifurcation parameter; instead this behaviour emerges, showing that the RNCRN is reproducing key features of the mathematical structure of the underlying ODE equation (A 7).

We use algorithm 1 from [51] to approximate the target system equation (A 7) on $K_{1}=[3.5,6.5]\text{ and }K_{2}=[3.5,6.5]$ with a single-layer RNCRN (not parametrized). Tolerance $\varepsilon \approx 10^{0}$ is met with an RNCRN with M=6 chemical perceptrons and coefficients $\beta_{1}=\beta_{2}=1$, γ=1, and

α1=(0.492−0.1172.229−0.708−1.1461.363),α2=(−0.2830.6572.199−1.294−2.6323.118),θ=(2.24310.83810.718−3.1647.9593.002),(A8)ω1=(−3.6720.911−1.3910.241−0.454−0.077) and ω2=(2.091−3.981−1.4331.058−0.922−0.073),

where $\alpha_{i}=(\alpha_{i,1},\alpha_{i,2},\ldots ,\alpha_{i,6}{)}^{⊤}$ for i=1,2, $\omega_{i}=(\omega_{1,i},\omega_{2,i},\ldots ,\omega_{6,i}{)}^{⊤}$ for i=1,2 and $\theta =(\theta_{1},\theta_{2},\ldots ,\theta_{6}{)}^{⊤}$. The reduced ODEs are given by

(A 9) $$\begin{array}{rl} \frac{d{\tilde{x}}_{1}}{dt} & =g_{1}({\tilde{x}}_{1},{\tilde{x}}_{2})=1+{\tilde{x}}_{1}\sum_{j=1}^{6}\alpha_{1,j}\sigma_{1}\left(\omega_{j,1}{\tilde{x}}_{1}+\omega_{j,2}{\tilde{x}}_{2}+\theta_{j}\right)\\ \text{and}\;\frac{d{\tilde{x}}_{2}}{dt} & =g_{2}({\tilde{x}}_{1},{\tilde{x}}_{2})=1+{\tilde{x}}_{2}\sum_{j=1}^{6}\alpha_{2,j}\sigma_{1}\left(\omega_{j,1}{\tilde{x}}_{1}+\omega_{j,2}{\tilde{x}}_{2}+\theta_{j}\right), \end{array}$$

whereas the full ODEs read

(A 10) $$\begin{array}{rlrl} \frac{dx_{1}}{dt} & =1+x_{1}\left(\sum_{j=1}^{6}\alpha_{1,j}y_{j}\right),\;\; & & \;\;\;\;\frac{dx_{2}}{dt}=1+x_{2}\left(\sum_{j=1}^{6}\alpha_{2,j}y_{j}\right),\\ \mu \frac{dy_{1}}{dt} & =1+\left(\sum_{i=1}^{2}\omega_{1,i}x_{i}+\theta_{1}\right)y_{1}-y_{1}^{2},\;\; & & \;\;\;\;\mu \frac{dy_{2}}{dt}=1+\left(\sum_{i=1}^{2}\omega_{2,i}x_{i}+\theta_{2}\right)y_{2}-y_{2}^{2},\\ \mu \frac{dy_{3}}{dt} & =1+\left(\sum_{i=1}^{2}\omega_{3,i}x_{i}+\theta_{3}\right)y_{3}-y_{3}^{2},\;\; & & \;\;\;\;\mu \frac{dy_{4}}{dt}=1+\left(\sum_{i=1}^{2}\omega_{4,i}x_{i}+\theta_{4}\right)y_{4}-y_{4}^{2},\\ \mu \frac{dy_{5}}{dt} & =1+\left(\sum_{i=1}^{2}\omega_{5,i}x_{i}+\theta_{5}\right)y_{5}-y_{5}^{2}\;\text{and}\;\; & & \;\;\;\;\mu \frac{dy_{6}}{dt}=1+\left(\sum_{i=1}^{2}\omega_{6,i}x_{i}+\theta_{6}\right)y_{6}-y_{6}^{2}. \end{array}$$

Here, we assume general initial concentrations: $x_{1}(0)=a_{1}\in [3.5,6.5]$ and $x_{2}(0)=a_{2}\in [3.5,6.5]$, and $y_{1}(0)=b_{1}\geq 0$, $y_{2}(0)=b_{2}\geq 0$, $y_{3}(0)=b_{3}\geq 0$, $y_{4}(0)=b_{4}\geq 0$, $y_{5}(0)=b_{5}\geq 0$ and $y_{6}(0)=b_{6}\geq 0$. The coefficients were rounded to three decimal places to quote in text, and the full precision coefficients are available in the code repository.

**Randomly perturbed rates.** By randomly perturbing the rate constants in equation (A 8) with 10% uniform noise, we found sets of rate constants that lead to the oscillator dynamical behaviour in a Hopf bifurcation, e.g.

(A 11) $$\begin{array}{rl} \alpha_{1} & =\left(\begin{array}{c} 0.532\\ -0.106\\ 2.315\\ -0.693\\ -1.116\\ 1.467 \end{array}\right),\;\;\alpha_{2}=\left(\begin{array}{c} -0.286\\ 0.645\\ 1.989\\ -1.231\\ -2.614\\ 3.228 \end{array}\right),\;\;\theta =\left(\begin{array}{c} 2.025\\ 10.419\\ 11.140\\ -3.029\\ 8.754\\ 3.069 \end{array}\right),\;\;\\ \omega_{1} & =\left(\begin{array}{c} -3.912\\ 0.843\\ -1.345\\ 0.222\\ -0.481\\ -0.083 \end{array}\right)\text{ and}\;\;\omega_{2}=\left(\begin{array}{c} 2.059\\ -3.679\\ -1.552\\ 1.070\\ -0.929\\ -0.075 \end{array}\right), \end{array}$$

and, for completeness, we found examples of unistable behaviour including

(A 12) $$\begin{array}{rl} \alpha_{1} & =\left(\begin{array}{c} 0.493\\ -0.117\\ 2.223\\ -0.711\\ -1.147\\ 1.355 \end{array}\right),\;\;\alpha_{2}=\left(\begin{array}{c} -0.284\\ 0.657\\ 2.192\\ -1.299\\ -2.609\\ 3.110 \end{array}\right),\;\;\theta =\left(\begin{array}{c} 2.220\\ 10.930\\ 10.815\\ -3.142\\ 7.890\\ 2.991 \end{array}\right),\;\;\\ \omega_{1} & =\left(\begin{array}{c} -3.667\\ 0.920\\ -1.396\\ 0.241\\ -0.451\\ -0.077 \end{array}\right)\text{ and}\;\;\omega_{2}=\left(\begin{array}{c} 2.085\\ -4.016\\ -1.432\\ 1.065\\ -0.924\\ -0.073 \end{array}\right). \end{array}$$

## Appendix B. Piecewise systems

We now present the RNCRNs associated with §4.

### B.1. Simple unistable–bistable piecewise system

We use algorithm 3, a modified version of algorithm 1 from [51] with static executive species augmented with automatic differentiation (AD) techniques, to approximate the target system equation (4.1) on $K_{1}=[1,9]$ and $L_{1}=\{1\},L_{2}=\{0\}$ with a parametrized RNCRN. Tolerance $\varepsilon \approx 10^{1}$ is met with an RNCRN with M=3 chemical perceptrons and coefficients

(B 1) $$\begin{array}{rlrl} \frac{dx_{1}}{dt} & =35.700-54.403x_{1}y_{1}+188.696x_{1}y_{2}-112.261x_{1}y_{3}, & & \;\;x_{1}(0)=a_{1}\in K_{1},\\ \mu \frac{dy_{1}}{dt} & =52.618+137.501x_{1}y_{1}-477.661\lambda_{1}y_{1}-1513.810y_{1}-90.515y_{1}^{2}, & & \;\;y_{1}(0)=b_{1}\geq 0,\\ \mu \frac{dy_{2}}{dt} & =179.420-249.394x_{1}y_{2}-21\;494.744\lambda_{1}y_{2}-220.319y_{2}-12.931y_{2}^{2}, & & \;\;y_{2}(0)=b_{2}\geq 0,\\ \mu \frac{dy_{3}}{dt} & =130.184-225.249x_{1}y_{3}-1876.261\lambda_{1}y_{3}+854.494y_{3}-1122.973y_{3}^{2}, & & \;\;y_{3}(0)=b_{3}\geq 0\\ \text{and}\;\frac{d\lambda_{1}}{dt} & =0, & & \;\;\lambda_{1}(0)=c_{1}\in K_{r}. \end{array}$$

The reduced vector field of equation (B 1) is

(B 2) $$\begin{array}{rlrl} \frac{d{\tilde{x}}_{1}}{dt} & =35.700-54.403{\tilde{x}}_{1}{\tilde{y}}_{1}+188.696{\tilde{x}}_{1}{\tilde{y}}_{2}-112.261{\tilde{x}}_{1}{\tilde{y}}_{3}, & & \;\;\;x_{1}(0)=a_{1}\in K_{1},\\ {\tilde{y}}_{1} & =\sigma_{52.618,90.515}(137.501{\tilde{x}}_{1}-477.661{\tilde{\lambda }}_{1}-1513.810),\\ {\tilde{y}}_{2} & =\sigma_{179.420,12.931}(-249.394{\tilde{x}}_{1}-21\;494.744{\tilde{\lambda }}_{1}-220.319),\\ {\tilde{y}}_{3} & =\sigma_{130.184,1122.973}(-225.249{\tilde{x}}_{1}-1876.261{\tilde{\lambda }}_{1}+854.494)\\ \text{and}\;{\tilde{\lambda }}_{1} & =\lambda (0)=c_{1}, \end{array}$$

where the chemical activation function is defined as in equation (D 2).

### B.2. XOR-gated unistable–bistable piecewise system

We used algorithm 4 to train a classification-controlled RNCRN, i.e. equations (4.3) and (4.4), to approximate the more complicated unistable–bistable piecewise systems equation (4.6). Intuitively, algorithm 4 first trains a parametrized RNCRN to approximate a bifurcations between the desired dynamical regimes. Then, a feed-forward network of chemical perceptrons is trained to control the chemical concentration of the bifurcation parameter according to the desired classification boundaries of the environmental species.

In this appendix, we walk through the steps of algorithm 4 in the context of the example target system equation (4.6).

***Step 1.* Pointwise parametrized RNCRN.** For ease of demonstration, we use the RNCRN found in §4.1. We note from figure 3e that the bifurcation point is approximately at $10^{-2}$. Otherwise, apply the step **(a)** from algorithm 4.

***Step 2.* Quasi-static classification CRN.** We want to create a feed-forward classification that embeds the XOR logic from the target equation (4.6) into a CRN and triggers the appropriate dynamical regime. That is, we train a neural CRN to approximate the function

(B 3) $$\begin{array}{r} \bar{r}(\bar{\lambda_{1}},\bar{\lambda_{2}})=\left\{ \begin{array}{clc} & 1,\text{ for all }(\bar{\lambda_{1}},\bar{\lambda_{2}})\in L_{1}=\left(K_{1}\times K_{2}\right)\cup \left(K_{2}\times K_{1}\right), & \;\\ & 0,\text{ for all }(\bar{\lambda_{1}},\bar{\lambda_{2}})\in L_{2}=\left(K_{1}\times K_{1}\right)\cup \left(K_{2}\times K_{2}\right), & \; \end{array}\right. \end{array}$$

where $K_{1}=[0,1)$ and $K_{2}=[1,2]$. We define a structure of K=4 chemical perceptrons [56] that catalytically senses the environmental species $\Lambda_{1}$ and $\Lambda_{2}$. These sense chemical perceptrons then feed into a bifurcation species R, which also has a chemical-perceptron structure, that will later be connected to the parametrized RNCRN in place of the bifurcation parameter. The corresponding RRE of the aforementioned feed-forward chemical-perceptron structure is defined as

(B 4) $$\begin{array}{rlrl} \mu \frac{dz_{1}}{dt} & =0.01+2.726\lambda_{1}z_{1}-3.025\lambda_{2}z_{1}+0.296z_{1}-z_{1}^{2}, & & \;\;\;z_{1}(0)=c_{1}\geq 0,\\ \mu \frac{dz_{2}}{dt} & =0.01+2.259\lambda_{1}z_{2}+0.057\lambda_{2}z_{2}-2.328z_{2}-z_{2}^{2}, & & \;\;\;z_{2}(0)=c_{2}\geq 0,\\ \mu \frac{dz_{3}}{dt} & =0.01-0.051\lambda_{1}z_{3}-2.511\lambda_{2}z_{3}+2.582z_{3}-z_{3}^{2}, & & \;\;\;z_{3}(0)=c_{3}\geq 0,\\ \mu \frac{dz_{4}}{dt} & =0.01+0.158\lambda_{1}z_{4}-0.156\lambda_{2}z_{4}+0.666z_{4}-z_{4}^{2}, & & \;\;\;z_{4}(0)=c_{4}\geq 0,\\ \mu \frac{dr}{dt} & =0.01+4.528z_{1}r-5.719z_{2}r-5.648z_{3}r+0.777z_{4}r+0.682r-r^{2}, & & \;\;\;r(0)=d\geq 0,\\ \frac{d\lambda_{1}}{dt} & =0, & & \;\;\;\lambda_{1}(0)=e_{1}\geq 0\\ \text{and}\;\frac{d\lambda_{2}}{dt} & =0, & & \;\;\;\lambda_{2}(0)=e_{2}\geq 0, \end{array}$$

where $z_{1},\ldots ,z_{4}$ are the time-dependent molecular concentrations of the sense-chemical-perceptron species, $\lambda_{1}$ and $\lambda_{2}$ are the molecular concentrations of the environmental species (which are assumed to be static) and r is the molecular concentration of the bifurcation species. We found these rate constants by training an ANN with the chemical activation function, $\sigma_{\gamma }$, using standard machine learning methods then mapping the parameters back to the full chemical-perceptron RRE with an additional time-scale parameter μ.

***Step 3.* Dynamical approximation.** The complete classification-controlled RNCRN is constructed from the combination of the CRNs that produce reaction-rate equation (B 1) and reaction-rate equation (B 4). As shown in figure 5, the μ=0.001 parameter was found to be sufficient.

(B 5) $$\begin{array}{rlrl} \frac{dx_{1}}{dt} & =35.700-54.403x_{1}y_{1}+188.696x_{1}y_{2}-112.261x_{1}y_{3}, & & \;\;\;x_{1}(0)=a_{1}\in K_{1},\\ \mu \frac{dy_{1}}{dt} & =52.618+137.501x_{1}y_{1}-477.661ry_{1}-1513.810y_{1}-90.515y_{1}^{2}, & & \;\;\;y_{1}(0)=b_{1}\geq 0,\\ \mu \frac{dy_{2}}{dt} & =179.420-249.394x_{1}y_{2}-21\;494.744ry_{2}-220.319y_{2}-12.931y_{2}^{2}, & & \;\;\;y_{2}(0)=b_{2}\geq 0,\\ \mu \frac{dy_{3}}{dt} & =130.184-225.249x_{1}y_{3}-1876.261ry_{3}+854.494y_{3}-1122.973y_{3}^{2}, & & \;\;\;y_{3}(0)=b_{3}\geq 0,\\ \mu \frac{dz_{1}}{dt} & =0.01+2.726\lambda_{1}z_{1}-3.025\lambda_{2}z_{1}+0.296z_{1}-z_{1}^{2}, & & \;\;\;z_{1}(0)=c_{1}\geq 0,\\ \mu \frac{dz_{2}}{dt} & =0.01+2.259\lambda_{1}z_{2}+0.057\lambda_{2}z_{2}-2.328z_{2}-z_{2}^{2}, & & \;\;\;z_{2}(0)=c_{2}\geq 0,\\ \mu \frac{dz_{3}}{dt} & =0.01-0.051\lambda_{1}z_{3}-2.511\lambda_{2}z_{3}+2.582z_{3}-z_{3}^{2}, & & \;\;\;z_{3}(0)=c_{3}\geq 0,\\ \mu \frac{dz_{4}}{dt} & =0.01+0.158\lambda_{1}z_{4}-0.156\lambda_{2}z_{4}+0.666z_{4}-z_{4}^{2}, & & \;\;\;z_{4}(0)=c_{4}\geq 0,\\ \mu \frac{dr}{dt} & =0.01+4.528z_{1}r-5.719z_{2}r-5.648z_{3}r+0.777z_{4}r+0.682r-r^{2}, & & \;\;\;r(0)=d\geq 0,\\ \frac{d\lambda_{1}}{dt} & =0, & & \;\;\;\lambda_{1}(0)=e_{1}\geq 0\\ \text{and}\;\frac{d\lambda_{2}}{dt} & =0, & & \;\;\;\lambda_{2}(0)=e_{2}\geq 0. \end{array}$$

## Appendix C. Example data-defined limit cycles and switching

We now train RNCRNs on training data generated by algorithm 1 to learn data-defined dynamics. In figure 9, we emphasize the artificial data generated by algorithm 1 by showing close-ups of the data used in the examples in §5.

### C.1. Single circular limit cycle (attractor)

Using the parametric equation defined in §5.1 and algorithms 1 and 5, we found an RNCRN with M=5 chemical perceptrons and coefficients $\beta_{1}=16.861$, $\beta_{2}=13.955$, and

(C 1) $$\begin{array}{rl} \alpha_{1} & =\left(\begin{array}{c} -22.642\\ -3.214\\ -13.344\\ -5.983\\ 11.413 \end{array}\right),\;\;\alpha_{2}=\left(\begin{array}{c} 20.600\\ -31.984\\ 4.736\\ -9.337\\ -11.829 \end{array}\right),\;\;\theta =\left(\begin{array}{c} 70.437\\ -86.509\\ -62.502\\ 139.089\\ 77.599 \end{array}\right),\;\;\\ \omega_{1} & =\left(\begin{array}{c} -46.744\\ -21.405\\ 63.399\\ -31.199\\ 20.670 \end{array}\right),\;\;\omega_{2}=\left(\begin{array}{c} -1.875\\ 30.682\\ -47.177\\ -27.589\\ -48.644 \end{array}\right),\\ \gamma & =\left(\begin{array}{c} 22.316\\ 23.786\\ 4.064\\ 0.484\\ 2.106 \end{array}\right)\text{ and}\;\;\tau =\left(\begin{array}{c} 111.885\\ 106.208\\ 113.115\\ 68.879\\ 94.832 \end{array}\right), \end{array}$$

where $\alpha_{i}=(\alpha_{i,1},\alpha_{i,2},\ldots ,\alpha_{i,5}{)}^{⊤}$ for i=1,2, $\omega_{i}=(\omega_{1,i},\omega_{2,i},\ldots ,\omega_{5,i}{)}^{⊤}$ for i=1,2, $\theta =(\theta_{1},\theta_{2},\ldots ,\theta_{5}{)}^{⊤}$, $\gamma =(\gamma_{1},\gamma_{2},\ldots ,\gamma_{5}{)}^{⊤}$ and $\tau =(\tau_{1},\tau_{2},\ldots ,\tau_{5}{)}^{⊤}$. The reduced ODEs are given by

(C 2) $$\begin{array}{rl} \frac{d{\tilde{x}}_{1}}{dt} & =g_{1}({\tilde{x}}_{1},{\tilde{x}}_{2})=16.861+{\tilde{x}}_{1}\sum_{j=1}^{5}\alpha_{1,j}\sigma_{\gamma_{j},\tau_{j}}\left(\omega_{j,1}{\tilde{x}}_{1}+\omega_{j,2}{\tilde{x}}_{2}+\theta_{j}\right)\\ \text{and}\;\frac{d{\tilde{x}}_{2}}{dt} & =g_{2}({\tilde{x}}_{1},{\tilde{x}}_{2})=13.955+{\tilde{x}}_{2}\sum_{j=1}^{5}\alpha_{2,j}\sigma_{\gamma_{j},\tau_{j}}\left(\omega_{j,1}{\tilde{x}}_{1}+\omega_{j,2}{\tilde{x}}_{2}+\theta_{j}\right), \end{array}$$

whereas the full ODEs read

(C 3) $$\begin{array}{rlrl} \frac{dx_{1}}{dt} & =16.861+x_{1}\left(\sum_{j=1}^{5}\alpha_{1,j}y_{j}\right),\;\; & & \;\;\;\;\frac{dx_{2}}{dt}=13.955+x_{2}\left(\sum_{j=1}^{5}\alpha_{2,j}y_{j}\right),\\ \mu \frac{dy_{1}}{dt} & =22.316+\left(\sum_{i=1}^{2}\omega_{1,i}x_{i}+\theta_{1}\right)y_{1}-111.885y_{1}^{2},\;\; & & \;\;\;\;\mu \frac{dy_{2}}{dt}=23.786+\left(\sum_{i=1}^{2}\omega_{2,i}x_{i}+\theta_{2}\right)y_{2}-106.208y_{2}^{2},\\ \mu \frac{dy_{3}}{dt} & =4.064+\left(\sum_{i=1}^{2}\omega_{3,i}x_{i}+\theta_{3}\right)y_{3}-113.115y_{3}^{2},\;\; & & \;\;\;\;\mu \frac{dy_{4}}{dt}=0.484+\left(\sum_{i=1}^{2}\omega_{4,i}x_{i}+\theta_{4}\right)y_{4}-68.879y_{4}^{2}\\ \text{and}\;\mu \frac{dy_{5}}{dt} & =2.106+\left(\sum_{i=1}^{2}\omega_{5,i}x_{i}+\theta_{5}\right)y_{5}-94.832y_{5}^{2}. \end{array}$$

Here, we assume general initial concentrations: $x_{1}(0)=a_{1}>0$ and $x_{2}(0)=a_{2}>0$, and $y_{1}(0)=b_{1}\geq 0$, $y_{2}(0)=b_{2}\geq 0$, $y_{3}(0)=b_{3}\geq 0$, $y_{4}(0)=b_{4}\geq 0$ and $y_{5}(0)=b_{5}\geq 0$. The coefficients were rounded to three decimal places to quote in text, and the full precision coefficients are available in the code repository.

### C.2. Single circular limit cycle (repeller)

As described in §5.1, training data generated by algorithm 1 (with an inverted normal direction) and equation (5.1) were used to train an RNCRN using the approach laid out in and algorithm 5. Figure 10 shows the vector field of the reduced RNCRN approximation of the circular repeller orbit.

This training produced an RNCRN with M=5 chemical perceptrons and coefficients $\beta_{1}=81.948$, $\beta_{2}=77.952$, and

(C 4) $$\begin{array}{rl} \alpha_{1} & =\left(\begin{array}{c} 43.091\\ -81.455\\ -178.720\\ -6.523\\ -41.211 \end{array}\right),\;\;\alpha_{2}=\left(\begin{array}{c} -174.745\\ -5.587\\ 51.884\\ -69.925\\ -45.358 \end{array}\right),\;\;\theta =\left(\begin{array}{c} -306.069\\ 422.215\\ -377.697\\ 445.634\\ 411.775 \end{array}\right),\;\;\\ \omega_{1} & =\left(\begin{array}{c} 125.349\\ -355.600\\ -354.684\\ -48.422\\ -48.630 \end{array}\right),\;\;\omega_{2}=\left(\begin{array}{c} -404.272\\ -34.736\\ 130.538\\ -344.728\\ -43.372 \end{array}\right),\\ \gamma & =\left(\begin{array}{c} 87.125\\ 31.103\\ 86.664\\ 26.322\\ 1.338 \end{array}\right)\text{ and}\;\;\tau =\left(\begin{array}{c} 1.719\\ 236.683\\ 2.733\\ 239.852\\ 488.075 \end{array}\right), \end{array}$$

where $\alpha_{i}=(\alpha_{i,1},\alpha_{i,2},\ldots ,\alpha_{i,5}{)}^{⊤}$ for i=1,2, $\omega_{i}=(\omega_{1,i},\omega_{2,i},\ldots ,\omega_{5,i}{)}^{⊤}$ for i=1,2, $\theta =(\theta_{1},\theta_{2},\ldots ,\theta_{5}{)}^{⊤}$, $\gamma =(\gamma_{1},\gamma_{2},\ldots ,\gamma_{5}{)}^{⊤}$ and $\tau =(\tau_{1},\tau_{2},\ldots ,\tau_{5}{)}^{⊤}$. The reduced ODEs are given by

(C 5) $$\begin{array}{rl} \frac{d{\tilde{x}}_{1}}{dt} & =g_{1}({\tilde{x}}_{1},{\tilde{x}}_{2})=81.948+{\tilde{x}}_{1}\sum_{j=1}^{5}\alpha_{1,j}\sigma_{\gamma_{j},\tau_{j}}\left(\omega_{j,1}{\tilde{x}}_{1}+\omega_{j,2}{\tilde{x}}_{2}+\theta_{j}\right)\\ \text{and}\;\frac{d{\tilde{x}}_{2}}{dt} & =g_{2}({\tilde{x}}_{1},{\tilde{x}}_{2})=77.952+{\tilde{x}}_{2}\sum_{j=1}^{5}\alpha_{2,j}\sigma_{\gamma_{j},\tau_{j}}\left(\omega_{j,1}{\tilde{x}}_{1}+\omega_{j,2}{\tilde{x}}_{2}+\theta_{j}\right), \end{array}$$

while the full ODEs read

(C 6) $$\begin{array}{rlrl} \frac{dx_{1}}{dt} & =81.948+x_{1}\left(\sum_{j=1}^{5}\alpha_{1,j}y_{j}\right),\;\; & & \;\;\;\frac{dx_{2}}{dt}=77.952+x_{2}\left(\sum_{j=1}^{5}\alpha_{2,j}y_{j}\right),\\ \mu \frac{dy_{1}}{dt} & =87.125+\left(\sum_{i=1}^{2}\omega_{1,i}x_{i}+\theta_{1}\right)y_{1}-1.719y_{1}^{2},\;\; & & \;\;\;\mu \frac{dy_{2}}{dt}=31.103+\left(\sum_{i=1}^{2}\omega_{2,i}x_{i}+\theta_{2}\right)y_{2}-236.683y_{2}^{2},\\ \mu \frac{dy_{3}}{dt} & =86.664+\left(\sum_{i=1}^{2}\omega_{3,i}x_{i}+\theta_{3}\right)y_{3}-2.733y_{3}^{2},\;\; & & \;\;\;\mu \frac{dy_{4}}{dt}=26.322+\left(\sum_{i=1}^{2}\omega_{4,i}x_{i}+\theta_{4}\right)y_{4}-239.852y_{4}^{2}\\ \text{and}\;\mu \frac{dy_{5}}{dt} & =1.338+\left(\sum_{i=1}^{2}\omega_{5,i}x_{i}+\theta_{5}\right)y_{5}-488.075y_{5}^{2}. \end{array}$$

Here, we assume general initial concentrations: $x_{1}(0)=a_{1}>0$ and $x_{2}(0)=a_{2}>0$, and $y_{1}(0)=b_{1}\geq 0$, $y_{2}(0)=b_{2}\geq 0$, $y_{3}(0)=b_{3}\geq 0$, $y_{4}(0)=b_{4}\geq 0$ and $y_{5}(0)=b_{5}\geq 0$. The coefficients were rounded to three decimal places to quote in text, and the full precision coefficients are available in the code repository.

### C.3. Single circular limit cycle (linear)

In §5, algorithm 1’s padding points $Q_{d,k}^{\pm }$ have the associated vector field $\text{d}x/\text{d}t=\eta {\hat{v}}_{d,d+1}\mp \eta {\hat{n}}_{d,d+1}\exp\left(\kappa \delta k\right)$ with the exponential dependence on the distance δk. In this example, we show that replacing the exponential dependence with a linear dependence while training the RNCRN can also produce the desired data-defined circular orbit, i.e. we use the vector field $\text{d}x/\text{d}t=\eta {\hat{v}}_{d,d+1}\mp \eta {\hat{n}}_{d,d+1}(\kappa \delta k)$ for padding points.

After generating data with the aforementioned linear modification to algorithm 1 on the circular equation (5.1) with K=20, δ=0.01, κ=10 and η=1, we trained the RNCRN using algorithm 5. Figure 11 shows the vector field of the reduced RNCRN approximation of the linear circular attractor orbit.

This training produced an RNCRN with M=5 chemical perceptrons and coefficients $\beta_{1}=16.552$, $\beta_{2}=23.165$, and

(C 7) $$\begin{array}{rl} \alpha_{1} & =\left(\begin{array}{c} -8.177\\ -16.814\\ -21.754\\ -7.906\\ 15.501 \end{array}\right),\;\;\alpha_{2}=\left(\begin{array}{c} 26.539\\ -22.747\\ 4.566\\ -6.631\\ -30.088 \end{array}\right),\;\;\theta =\left(\begin{array}{c} 59.477\\ -35.099\\ -60.028\\ 90.654\\ 54.644 \end{array}\right),\;\;\\ \omega_{1} & =\left(\begin{array}{c} -33.517\\ -28.584\\ 39.503\\ -22.314\\ 0.131 \end{array}\right),\;\;\omega_{2}=\left(\begin{array}{c} -9.963\\ 19.259\\ -40.894\\ -17.509\\ -40.181 \end{array}\right),\\ \gamma & =\left(\begin{array}{c} 23.272\\ 27.928\\ 8.608\\ 0.397\\ 20.500 \end{array}\right)\text{ and}\;\;\tau =\left(\begin{array}{c} 56.930\\ 62.523\\ 61.866\\ 36.392\\ 46.726 \end{array}\right), \end{array}$$

where $\alpha_{i}=(\alpha_{i,1},\alpha_{i,2},\ldots ,\alpha_{i,5}{)}^{⊤}$ for i=1,2, $\omega_{i}=(\omega_{1,i},\omega_{2,i},\ldots ,\omega_{5,i}{)}^{⊤}$ for i=1,2, $\theta =(\theta_{1},\theta_{2},\ldots ,\theta_{5}{)}^{⊤}$, $\gamma =(\gamma_{1},\gamma_{2},\ldots ,\gamma_{5}{)}^{⊤}$ and $\tau =(\tau_{1},\tau_{2},\ldots ,\tau_{5}{)}^{⊤}$. The reduced ODEs are given by

(C 8) $$\begin{array}{rl} \frac{d{\tilde{x}}_{1}}{dt} & =g_{1}({\tilde{x}}_{1},{\tilde{x}}_{2})=16.552+{\tilde{x}}_{1}\sum_{j=1}^{5}\alpha_{1,j}\sigma_{\gamma_{j},\tau_{j}}\left(\omega_{j,1}{\tilde{x}}_{1}+\omega_{j,2}{\tilde{x}}_{2}+\theta_{j}\right)\\ \text{and}\;\frac{d{\tilde{x}}_{2}}{dt} & =g_{2}({\tilde{x}}_{1},{\tilde{x}}_{2})=23.165+{\tilde{x}}_{2}\sum_{j=1}^{5}\alpha_{2,j}\sigma_{\gamma_{j},\tau_{j}}\left(\omega_{j,1}{\tilde{x}}_{1}+\omega_{j,2}{\tilde{x}}_{2}+\theta_{j}\right), \end{array}$$

whereas the full ODEs read

(C 9) $$\begin{array}{rlrl} \frac{dx_{1}}{dt} & =16.552+x_{1}\left(\sum_{j=1}^{5}\alpha_{1,j}y_{j}\right),\;\; & & \;\;\;\frac{dx_{2}}{dt}=23.165+x_{2}\left(\sum_{j=1}^{5}\alpha_{2,j}y_{j}\right),\\ \mu \frac{dy_{1}}{dt} & =23.272+\left(\sum_{i=1}^{2}\omega_{1,i}x_{i}+\theta_{1}\right)y_{1}-56.930y_{1}^{2},\;\; & & \;\;\;\mu \frac{dy_{2}}{dt}=27.928+\left(\sum_{i=1}^{2}\omega_{2,i}x_{i}+\theta_{2}\right)y_{2}-62.523y_{2}^{2},\\ \mu \frac{dy_{3}}{dt} & =8.608+\left(\sum_{i=1}^{2}\omega_{3,i}x_{i}+\theta_{3}\right)y_{3}-61.866y_{3}^{2},\;\; & & \;\;\;\mu \frac{dy_{4}}{dt}=0.397+\left(\sum_{i=1}^{2}\omega_{4,i}x_{i}+\theta_{4}\right)y_{4}-36.392y_{4}^{2}\\ \text{and}\;\mu \frac{dy_{5}}{dt} & =20.500+\left(\sum_{i=1}^{2}\omega_{5,i}x_{i}+\theta_{5}\right)y_{5}-46.726y_{5}^{2}. \end{array}$$

Here, we assume general initial concentrations: $x_{1}(0)=a_{1}>0$ and $x_{2}(0)=a_{2}>0$, and $y_{1}(0)=b_{1}\geq 0$, $y_{2}(0)=b_{2}\geq 0$, $y_{3}(0)=b_{3}\geq 0$, $y_{4}(0)=b_{4}\geq 0$ and $y_{5}(0)=b_{5}\geq 0$. The coefficients were rounded to three decimal places to quote in text, and the full precision coefficients are available in the code repository.

### C.4. Heart-shaped limit cycle

As described in §5.2, training data generated by algorithm 1 and equation (5.2) were used to train an RNCRN using algorithm 5. This training produced an RNCRN with M=10 chemical perceptrons and coefficients $\beta_{1}=32.263$, $\beta_{2}=0.220$, and

(C 10) $$\begin{array}{rl} \alpha_{1} & =\left(\begin{array}{c} -206.685\\ 68.185\\ 63.034\\ 31.454\\ -1.046\\ -69.791\\ 3.335\\ 6.724\\ -4.768\\ -0.753 \end{array}\right),\;\;\alpha_{2}=\left(\begin{array}{c} 14.421\\ 361.350\\ 42.053\\ -1596.404\\ 11.659\\ 445.898\\ -26.598\\ 4.145\\ 9.770\\ -24.237 \end{array}\right),\;\;\theta =\left(\begin{array}{c} 399.866\\ -2885.359\\ -3238.521\\ -4674.896\\ 1634.028\\ -2261.417\\ 1268.967\\ 1348.322\\ -2966.115\\ -965.133 \end{array}\right),\;\;\\ \omega_{1} & =\left(\begin{array}{c} -752.176\\ -1404.888\\ -2126.800\\ -6923.930\\ -694.304\\ -1179.076\\ 44.345\\ -472.745\\ 2317.337\\ 388.654 \end{array}\right),\;\;\omega_{2}=\left(\begin{array}{c} -85.831\\ -2904.666\\ -3063.465\\ -3358.036\\ -126.843\\ -2667.009\\ -551.100\\ -404.818\\ -1782.721\\ 29.470 \end{array}\right),\\ \gamma & =\left(\begin{array}{c} 102.958\\ 317.511\\ 12.669\\ 422.712\\ 0.056\\ 435.578\\ 0.572\\ 0.087\\ 0.189\\ 10.109 \end{array}\right)\text{ and}\;\;\tau =\left(\begin{array}{c} 735.035\\ 6155.131\\ 5217.168\\ 2052.469\\ 1814.245\\ 6080.718\\ 2388.553\\ 519.255\\ 5648.693\\ 2210.594 \end{array}\right), \end{array}$$

where $\alpha_{i}=(\alpha_{i,1},\alpha_{i,2},\ldots ,\alpha_{i,10}{)}^{⊤}$ for i=1,2, $\omega_{i}=(\omega_{1,i},\omega_{2,i},\ldots ,\omega_{10,i}{)}^{⊤}$ for i=1,2, $\theta =(\theta_{1},\theta_{2},\ldots ,\theta_{10}{)}^{⊤}$, $\gamma =(\gamma_{1},\gamma_{2},\ldots ,\gamma_{10}{)}^{⊤}$ and $\tau =(\tau_{1},\tau_{2},\ldots ,\tau_{10}{)}^{⊤}$. The reduced ODEs are given by

(C 11) $$\begin{array}{rl} \frac{d{\tilde{x}}_{1}}{dt} & =g_{1}({\tilde{x}}_{1},{\tilde{x}}_{2})=32.263+{\tilde{x}}_{1}\sum_{j=1}^{10}\alpha_{1,j}\sigma_{\gamma_{j},\tau_{j}}\left(\omega_{j,1}{\tilde{x}}_{1}+\omega_{j,2}{\tilde{x}}_{2}+\theta_{j}\right)\\ \text{and}\;\frac{d{\tilde{x}}_{2}}{dt} & =g_{2}({\tilde{x}}_{1},{\tilde{x}}_{2})=0.220+{\tilde{x}}_{2}\sum_{j=1}^{10}\alpha_{2,j}\sigma_{\gamma_{j},\tau_{j}}\left(\omega_{j,1}{\tilde{x}}_{1}+\omega_{j,2}{\tilde{x}}_{2}+\theta_{j}\right), \end{array}$$

whereas the full ODEs read

(C 12) $$\begin{array}{rlrl} \frac{dx_{1}}{dt} & =32.263+x_{1}\left(\sum_{j=1}^{10}\alpha_{1,j}y_{j}\right),\;\; & & \;\;\;\frac{dx_{2}}{dt}=0.220+x_{2}\left(\sum_{j=1}^{10}\alpha_{2,j}y_{j}\right),\\ \mu \frac{dy_{1}}{dt} & =102.958+\left(\sum_{i=1}^{2}\omega_{1,i}x_{i}+\theta_{1}\right)y_{1}-735.035y_{1}^{2},\;\; & & \;\;\;\mu \frac{dy_{2}}{dt}=317.511+\left(\sum_{i=1}^{2}\omega_{2,i}x_{i}+\theta_{2}\right)y_{2}-6155.131y_{2}^{2},\\ \mu \frac{dy_{3}}{dt} & =12.669+\left(\sum_{i=1}^{2}\omega_{3,i}x_{i}+\theta_{3}\right)y_{3}-5217.168y_{3}^{2},\;\; & & \;\;\;\mu \frac{dy_{4}}{dt}=422.712+\left(\sum_{i=1}^{2}\omega_{4,i}x_{i}+\theta_{4}\right)y_{4}-2052.469y_{4}^{2},\\ \mu \frac{dy_{5}}{dt} & =0.056+\left(\sum_{i=1}^{2}\omega_{5,i}x_{i}+\theta_{5}\right)y_{5}-1814.245y_{5}^{2},\;\; & & \;\;\;\mu \frac{dy_{6}}{dt}=435.578+\left(\sum_{i=1}^{2}\omega_{6,i}x_{i}+\theta_{6}\right)y_{6}-6080.718y_{6}^{2},\\ \mu \frac{dy_{7}}{dt} & =0.572+\left(\sum_{i=1}^{2}\omega_{7,i}x_{i}+\theta_{7}\right)y_{7}-2388.553y_{7}^{2},\;\; & & \;\;\;\mu \frac{dy_{8}}{dt}=0.087+\left(\sum_{i=1}^{2}\omega_{8,i}x_{i}+\theta_{8}\right)y_{8}-519.255y_{8}^{2},\\ \mu \frac{dy_{9}}{dt} & =0.189+\left(\sum_{i=1}^{2}\omega_{9,i}x_{i}+\theta_{9}\right)y_{9}-5648.693y_{9}^{2}\;\text{and}\;\; & & \;\;\;\mu \frac{dy_{10}}{dt}=10.109+\left(\sum_{i=1}^{2}\omega_{10,i}x_{i}+\theta_{10}\right)y_{10}-2210.594y_{10}^{2}. \end{array}$$

Here, we assume general initial concentrations: $x_{1}(0)=a_{1}>0$ and $x_{2}(0)=a_{2}>0$, and $y_{1}(0)=b_{1}\geq 0$, $y_{2}(0)=b_{2}\geq 0$, $y_{3}(0)=b_{3}\geq 0$, $y_{4}(0)=b_{4}\geq 0$, $y_{5}(0)=b_{5}\geq 0$, $y_{6}(0)=b_{6}\geq 0$, $y_{7}(0)=b_{7}\geq 0$, $y_{8}(0)=b_{8}\geq 0$, $y_{9}(0)=b_{9}\geq 0$ and $y_{10}(0)=b_{10}\geq 0$. The coefficients were rounded to three decimal places to quote in text, and the full precision coefficients are available in the code repository.

### C.5. Multiple limit cycles

As described in §5.3, training data generated by algorithm 1 and equation (5.1) were used to train an RNCRN using algorithm 5. This training produced an RNCRN with M=15 chemical perceptrons and coefficients $\beta_{1}=18.815$, $\beta_{2}=18.135$, and

(C 13) $$\begin{array}{rl} \alpha_{1} & =\left(\begin{array}{c} 0.037\\ -44.974\\ -9.160\\ 0.511\\ -0.021\\ 0.567\\ -4.428\\ 0.549\\ -0.238\\ 0.241\\ -3.833\\ -14.058\\ -0.073\\ -0.177\\ 0.025 \end{array}\right),\;\;\alpha_{2}=\left(\begin{array}{c} 0.053\\ -35.678\\ -108.873\\ 0.039\\ 0.307\\ -21.397\\ 13.701\\ 0.520\\ -0.150\\ -0.041\\ -11.530\\ -2.218\\ 0.108\\ -0.217\\ 0.298 \end{array}\right),\;\;\theta =\left(\begin{array}{c} -2169.556\\ -63.584\\ -63.308\\ -2110.566\\ -2062.301\\ 330.702\\ 371.345\\ -2167.958\\ -2232.450\\ -2186.657\\ -2189.514\\ 366.100\\ -2077.129\\ -2345.668\\ -2176.678 \end{array}\right),\;\;\\ \omega_{1} & =\left(\begin{array}{c} -2613.137\\ -133.234\\ -85.431\\ -2471.896\\ -2582.940\\ -3.890\\ -85.207\\ -2593.340\\ -2869.315\\ -2738.075\\ -2545.244\\ -229.477\\ -2510.477\\ -2841.934\\ -2434.409 \end{array}\right),\;\;\omega_{2}=\left(\begin{array}{c} -2439.155\\ 79.236\\ -283.547\\ -2319.514\\ -2296.273\\ -185.955\\ -36.065\\ -2420.028\\ -2499.550\\ -2462.773\\ -2429.244\\ 4.170\\ -2312.757\\ -2641.583\\ -2347.650 \end{array}\right),\\ \gamma & =\left(\begin{array}{c} 0.046\\ 48.748\\ 55.087\\ 0.047\\ 0.607\\ 1.108\\ 0.359\\ 0.317\\ 0.203\\ 0.087\\ 0.574\\ 0.549\\ 0.069\\ 0.058\\ 0.043 \end{array}\right)\text{ and}\;\;\tau =\left(\begin{array}{c} 105.841\\ 632.562\\ 465.905\\ 5.862\\ 114.611\\ 388.573\\ 365.923\\ 28.740\\ 85.871\\ 30.612\\ 2.894\\ 411.963\\ 84.905\\ 75.267\\ 8.467 \end{array}\right), \end{array}$$

where $\alpha_{i}=(\alpha_{i,1},\alpha_{i,2},\ldots ,\alpha_{i,15}{)}^{⊤}$ for i=1,2, $\omega_{i}=(\omega_{1,i},\omega_{2,i},\ldots ,\omega_{15,i}{)}^{⊤}$ for i=1,2, $\theta =(\theta_{1},\theta_{2},\ldots ,\theta_{15}{)}^{⊤}$, $\gamma =(\gamma_{1},\gamma_{2},\ldots ,\gamma_{15}{)}^{⊤}$ and $\tau =(\tau_{1},\tau_{2},\ldots ,\tau_{15}{)}^{⊤}$. The reduced ODEs are given by

dx~1dt=g1(x~1,x~2)=18.815+x~1∑j=115α1,jσγj,τj(ωj,1x~1+ωj,2x~2+θj)(C14)anddx~2dt=g2(x~1,x~2)=18.135+x~2∑j=115α2,jσγj,τj(ωj,1x~1+ωj,2x~2+θj),

whereas the full ODEs read

(C 15) $$\begin{array}{rlrl} \frac{dx_{1}}{dt} & =18.815+x_{1}\left(\sum_{j=1}^{10}\alpha_{1,j}y_{j}\right),\;\; & & \;\;\;\frac{dx_{2}}{dt}=18.135+x_{2}\left(\sum_{j=1}^{10}\alpha_{2,j}y_{j}\right),\\ \mu \frac{dy_{1}}{dt} & =0.046+\left(\sum_{i=1}^{2}\omega_{1,i}x_{i}+\theta_{1}\right)y_{1}-105.841y_{1}^{2},\;\; & & \;\;\;\mu \frac{dy_{2}}{dt}=48.748+\left(\sum_{i=1}^{2}\omega_{2,i}x_{i}+\theta_{2}\right)y_{2}-632.562y_{2}^{2},\\ \mu \frac{dy_{3}}{dt} & =55.087+\left(\sum_{i=1}^{2}\omega_{3,i}x_{i}+\theta_{3}\right)y_{3}-465.905y_{3}^{2},\;\; & & \;\;\;\mu \frac{dy_{4}}{dt}=0.047+\left(\sum_{i=1}^{2}\omega_{4,i}x_{i}+\theta_{4}\right)y_{4}-5.862y_{4}^{2},\\ \mu \frac{dy_{5}}{dt} & =0.607+\left(\sum_{i=1}^{2}\omega_{5,i}x_{i}+\theta_{5}\right)y_{5}-114.611y_{5}^{2},\;\; & & \;\;\;\mu \frac{dy_{6}}{dt}=1.108+\left(\sum_{i=1}^{2}\omega_{6,i}x_{i}+\theta_{6}\right)y_{6}-388.573y_{6}^{2},\\ \mu \frac{dy_{7}}{dt} & =0.359+\left(\sum_{i=1}^{2}\omega_{7,i}x_{i}+\theta_{7}\right)y_{7}-365.923y_{7}^{2},\;\; & & \;\;\;\mu \frac{dy_{8}}{dt}=0.317+\left(\sum_{i=1}^{2}\omega_{8,i}x_{i}+\theta_{8}\right)y_{8}-28.740y_{8}^{2},\\ \mu \frac{dy_{9}}{dt} & =0.203+\left(\sum_{i=1}^{2}\omega_{9,i}x_{i}+\theta_{9}\right)y_{9}-85.871y_{9}^{2},\;\; & & \;\;\;\mu \frac{dy_{10}}{dt}=0.087+\left(\sum_{i=1}^{2}\omega_{10,i}x_{i}+\theta_{10}\right)y_{10}-30.612y_{10}^{2},\\ \mu \frac{dy_{11}}{dt} & =0.574+\left(\sum_{i=1}^{2}\omega_{11,i}x_{i}+\theta_{11}\right)y_{11}-2.894y_{11}^{2},\;\; & & \;\;\;\mu \frac{dy_{12}}{dt}=0.549+\left(\sum_{i=1}^{2}\omega_{12,i}x_{i}+\theta_{12}\right)y_{12}-411.963y_{12}^{2},\\ \mu \frac{dy_{13}}{dt} & =0.069+\left(\sum_{i=1}^{2}\omega_{13,i}x_{i}+\theta_{13}\right)y_{13}-84.905y_{13}^{2},\;\; & & \;\;\;\mu \frac{dy_{14}}{dt}=0.058+\left(\sum_{i=1}^{2}\omega_{14,i}x_{i}+\theta_{14}\right)y_{14}-75.267y_{14}^{2}\\ \text{and}\;\mu \frac{dy_{15}}{dt} & =0.043+\left(\sum_{i=1}^{2}\omega_{15,i}x_{i}+\theta_{15}\right)y_{15}-8.467y_{15}^{2}. \end{array}$$

Here, we assume general initial concentrations: $x_{1}(0)=a_{1}>0$ and $x_{2}(0)=a_{2}>0$, and $y_{1}(0)=b_{1}\geq 0$, $y_{2}(0)=b_{2}\geq 0$, $y_{3}(0)=b_{3}\geq 0$, $y_{4}(0)=b_{4}\geq 0$, $y_{5}(0)=b_{5}\geq 0$, $y_{6}(0)=b_{6}\geq 0$, $y_{7}(0)=b_{7}\geq 0$, $y_{8}(0)=b_{8}\geq 0$, $y_{9}(0)=b_{9}\geq 0$, $y_{10}(0)=b_{10}\geq 0$, $y_{11}(0)=b_{11}\geq 0$, $y_{12}(0)=b_{12}\geq 0$, $y_{13}(0)=b_{13}\geq 0$, $y_{14}(0)=b_{14}\geq 0$ and $y_{15}(0)=b_{15}\geq 0$. The coefficients were rounded to three decimal places to quote in text, and the full precision coefficients are available in the code repository.

### C.6. Toroidal limit cycle in three dimensions

Let us now show that algorithm 1, which is formulated in two dimensions, can be generalized into higher dimensions. In particular, consider the parametric equation for a curve on a torus

(C 16) $$\begin{array}{rl} x_{1}(s) & =3\cos(s)+\cos(10s)\cos(s)+6,\\ x_{2}(s) & =3\sin(s)+\cos(10s)\sin(s)+6\\ \text{and}\;x_{3}(s) & =\sin(10s)+2, \end{array}$$

where s∈[0,2π). Figure 12a shows three-dimensional training data produced from a generalization of algorithm 1 to three dimensions. Using this data and algorithm 5, we train an RNCRN with M=7 chemical perceptrons which, as demonstrated in figure 12b, numerically displays a three-dimensional stable limit cycle which is approximately of the desired toroidal shape.

The principal modification of the data generation algorithm was the generalization of the placement of padding points into more than one perpendicular direction.

**Higher dimensional data generation.** Given a list of points in higher dimensional $P_{d}\in R_{>}^{N}$ where N>2, we modify algorithm 1 such that the unit normal from part (a) is replaced with N−1 orthonormal unit vectors that form a basis with the ${\hat{v}}_{d,d+1}$ direction. Each of these orthonormal unit vectors produces additional padding points and is used to compute the local vector field approximations. Similar to the two-dimensional case, we apply the local vector field decay assignment in each of these N−1 orthonormal directions independently. As shown by figure 12a, considering each orthonormal direction generates artificial data points with vectors pointing towards the intended limit cycle starting from that independent direction.

To find the orthonormal basis, we recognize that a column vector ${\hat{v}}_{d,d+1}\in R{>}^{N\times 1}$ can be decomposed into an orthonormal matrix $Q\in R{>}^{N\times N}$ and a scalar R∈R via QR decomposition where the first column of Q will be linearly dependent on ${\hat{v}}_{d,d+1}$. It follows that the orthonormal unit vectors are the N−1 remaining column vectors of Q.

**RNCRN parameters.** Using the higher dimensional data-generation modification of algorithms 1 and 5 for training, we found an M=7 RNCRN with coefficients $\beta_{1}=1.570$, $\beta_{2}=0.408$, $\beta_{3}=6.885$, and

(C 17) $$\begin{array}{rl} \alpha_{1} & =\left(\begin{array}{c} -5.227\\ -6.586\\ 0.332\\ -1.297\\ 1.037\\ -0.752\\ 7.607 \end{array}\right),\;\;\alpha_{2}=\left(\begin{array}{c} -0.755\\ -5.934\\ -3.663\\ 14.209\\ 1.077\\ 1.567\\ 2.768 \end{array}\right),\;\;\alpha_{3}=\left(\begin{array}{c} 30.463\\ -19.509\\ 23.421\\ -20.966\\ -45.739\\ 4.597\\ 3.169 \end{array}\right),\;\;\theta =\left(\begin{array}{c} 292.047\\ -280.508\\ 261.422\\ -255.093\\ 262.072\\ -198.768\\ -139.727 \end{array}\right),\;\;\\ \omega_{1} & =\left(\begin{array}{c} -39.116\\ -17.292\\ -3.625\\ 7.586\\ -9.729\\ 207.473\\ -109.965 \end{array}\right),\;\;\omega_{2}=\left(\begin{array}{c} -2.949\\ -1.592\\ -36.064\\ -105.612\\ -9.325\\ -327.333\\ -28.268 \end{array}\right),\omega_{3}=\left(\begin{array}{c} -53.870\\ 111.653\\ -32.435\\ 145.520\\ -67.332\\ -308.171\\ 344.645 \end{array}\right),\\ \gamma & =\left(\begin{array}{c} 5.488\\ 7.535\\ 3.963\\ 9.258\\ 6.007\\ 0.004\\ 5.764 \end{array}\right)\text{ and}\;\;\tau =\left(\begin{array}{c} 666.189\\ 812.839\\ 496.655\\ 912.664\\ 428.803\\ 713.388\\ 4637.415 \end{array}\right), \end{array}$$

where $\alpha_{i}=(\alpha_{i,1},\alpha_{i,2},\ldots ,\alpha_{i,7}{)}^{⊤}$ for i=1,2,3, $\omega_{i}=(\omega_{1,i},\omega_{2,i},\ldots ,\omega_{7,i}{)}^{⊤}$ for i=1,2,3, $\theta =(\theta_{1},\theta_{2},\ldots ,\theta_{7}{)}^{⊤}$, $\gamma =(\gamma_{1},\gamma_{2},\ldots ,\gamma_{7}{)}^{⊤}$ and $\tau =(\tau_{1},\tau_{2},\ldots ,\tau_{7}{)}^{⊤}$. The reduced ODEs are given by

(C 18) $$\begin{array}{rl} \frac{d{\tilde{x}}_{1}}{dt} & =g_{1}({\tilde{x}}_{1},{\tilde{x}}_{2},{\tilde{x}}_{3})=1.570+{\tilde{x}}_{1}\sum_{j=1}^{7}\alpha_{1,j}\sigma_{\gamma_{j},\tau_{j}}\left(\omega_{j,1}{\tilde{x}}_{1}+\omega_{j,2}{\tilde{x}}_{2}+\omega_{j,3}{\tilde{x}}_{3}+\theta_{j}\right),\\ \frac{d{\tilde{x}}_{2}}{dt} & =g_{2}({\tilde{x}}_{1},{\tilde{x}}_{2},{\tilde{x}}_{3})=0.408+{\tilde{x}}_{2}\sum_{j=1}^{7}\alpha_{2,j}\sigma_{\gamma_{j},\tau_{j}}\left(\omega_{j,1}{\tilde{x}}_{1}+\omega_{j,2}{\tilde{x}}_{2}+\omega_{j,3}{\tilde{x}}_{3}+\theta_{j}\right)\\ \text{and}\;\frac{d{\tilde{x}}_{3}}{dt} & =g_{3}({\tilde{x}}_{1},{\tilde{x}}_{2},{\tilde{x}}_{3})=6.885+{\tilde{x}}_{3}\sum_{j=1}^{7}\alpha_{3,j}\sigma_{\gamma_{j},\tau_{j}}\left(\omega_{j,1}{\tilde{x}}_{1}+\omega_{j,2}{\tilde{x}}_{2}+\omega_{j,3}{\tilde{x}}_{3}+\theta_{j}\right), \end{array}$$

whereas the full ODEs read

(C 19) $$\begin{array}{rlrl} \frac{dx_{1}}{dt} & =1.570+x_{1}\left(\sum_{j=1}^{7}\alpha_{1,j}y_{j}\right),\;\; & & \;\;\;\frac{dx_{2}}{dt}=0.408+x_{2}\left(\sum_{j=1}^{7}\alpha_{2,j}y_{j}\right),\\ \frac{dx_{3}}{dt} & =6.885+x_{3}\left(\sum_{j=1}^{7}\alpha_{3,j}y_{j}\right),\;\; & & \;\;\;\mu \frac{dy_{1}}{dt}=5.488+\left(\sum_{i=1}^{3}\omega_{1,i}x_{i}+\theta_{1}\right)y_{1}-666.189y_{1}^{2},\\ \mu \frac{dy_{2}}{dt} & =7.535+\left(\sum_{i=1}^{3}\omega_{2,i}x_{i}+\theta_{2}\right)y_{2}-812.839y_{2}^{2},\;\; & & \;\;\;\mu \frac{dy_{3}}{dt}=3.963+\left(\sum_{i=1}^{3}\omega_{3,i}x_{i}+\theta_{3}\right)y_{3}-496.655y_{3}^{2},\\ \mu \frac{dy_{4}}{dt} & =9.258+\left(\sum_{i=1}^{3}\omega_{4,i}x_{i}+\theta_{4}\right)y_{4}-912.664y_{4}^{2},\;\; & & \;\;\;\mu \frac{dy_{5}}{dt}=6.007+\left(\sum_{i=1}^{3}\omega_{5,i}x_{i}+\theta_{5}\right)y_{5}-428.803y_{5}^{2},\\ \mu \frac{dy_{6}}{dt} & =0.004+\left(\sum_{i=1}^{3}\omega_{6,i}x_{i}+\theta_{6}\right)y_{6}-713.338y_{6}^{2}\;\text{and}\;\; & & \;\;\;\mu \frac{dy_{7}}{dt}=5.764+\left(\sum_{i=1}^{3}\omega_{7,i}x_{i}+\theta_{7}\right)y_{7}-4637.415y_{7}^{2}. \end{array}$$

Here, we assume general initial concentrations: $x_{1}(0)=a_{1}>0$, $x_{2}(0)=a_{2}>0$ and $x_{3}(0)=a_{3}>0$, and $y_{1}(0)=b_{1}\geq 0$, $y_{2}(0)=b_{2}\geq 0$, $y_{3}(0)=b_{3}\geq 0$, $y_{4}(0)=b_{4}\geq 0$, $y_{5}(0)=b_{5}\geq 0$, $y_{6}(0)=b_{6}\geq 0$ and $y_{7}(0)=b_{7}\geq 0$. The coefficients were rounded to three decimal places to quote in text, and the full precision coefficients are available in the code repository.

### C.7. Equilibrium-oscillation piecewise system with data-defined oscillations

We now detail the construction of the classification-controlled RNCRNs applied to approximate the equilibrium-oscillation piecewise system with data-defined oscillations (as introduced in §5.4). We use the general method to train a classification-controlled RNCRN, as defined in algorithm 4, with appropriate modification to use data-defined vector fields.

***Step 1.* Pointwise parametrized RNCRN.** Two dynamical states were defined: a cyclic attractor and a stable equilibrium. First, using algorithm 1, an attractor was generated for a parametric orbit, equation (5.1), centred on $b_{1}=b_{2}=1.5$ with radius a=1. Second, using the ODEs $dx_{1}/dt=0.5-x_{1}$ and $dx_{2}/dt=0.5-x_{2}$, the vector field of the stable equilibrium was used over compact subset $K_{1}=[0.01,3],K_{2}=[0.01,3]$. Using the associated control points $r_{1}=1$ and $r_{2}=0.1$, a modified algorithm 3 (i.e. allowing for data-defined vector fields as in algorithm 5) was used to find a pointwise parametrized RNCRN that bifurcates between the two target dynamics.

The resulting parametrized RNCRN was found with tolerance $\varepsilon \approx 10^{-1}$ met with an RNCRN with M=5 chemical perceptrons and coefficients $\beta_{1}=0.790,\beta_{2}=0.858$,

(C 20) $$\begin{array}{rl} \alpha_{1} & =\left(\begin{array}{c} 89.517\\ -13.080\\ 0.321\\ -98.540\\ -69.707 \end{array}\right),\;\;\alpha_{2}=\left(\begin{array}{c} 96.384\\ -50.709\\ -24.353\\ -11.676\\ -124.643 \end{array}\right),\;\;\theta =\left(\begin{array}{c} -1654.188\\ 578.636\\ -2579.316\\ -3291.187\\ -1568.960 \end{array}\right),\;\;\tau =\left(\begin{array}{c} 38.776\\ 8373.519\\ 3629.027\\ 13\;631.220\\ 3385.638 \end{array}\right),\;\;\\ \omega_{1} & =\left(\begin{array}{c} -1357.492\\ -727.745\\ -276.335\\ -2029.546\\ -239.827 \end{array}\right),\;\;\omega_{2}=\left(\begin{array}{c} -1128.933\\ -802.213\\ 694.332\\ 295.107\\ -775.896 \end{array}\right),\psi =\left(\begin{array}{c} 2380.343\\ 1605.154\\ 1198.530\\ 2737.624\\ -8382.229 \end{array}\right)\text{ and }\gamma =\left(\begin{array}{c} 75.071\\ 8.557\\ 5.288\\ 73.001\\ 70.181 \end{array}\right), \end{array}$$

where $\alpha_{i}=(\alpha_{i,1},\alpha_{i,2},\ldots ,\alpha_{i,5}{)}^{⊤}$ for i=1,2, $\omega_{i}=(\omega_{1,i},\omega_{2,i},\ldots ,\omega_{5,i}{)}^{⊤}$ for i=1,2, $\theta =(\theta_{1},\theta_{2},\ldots ,\theta_{5}{)}^{⊤}$, $\tau =(\tau_{1},\tau_{2},\ldots ,\tau_{5}{)}^{⊤}$, $\psi =(\psi_{1},\psi_{2},\ldots ,\psi_{5}{)}^{⊤}$ and $\gamma =(\gamma_{1},\gamma_{2},\ldots ,\gamma_{5}{)}^{⊤}$. The reduced ODEs are given by

(C 21) $$\begin{array}{rl} \frac{d{\tilde{x}}_{1}}{dt} & =g_{1}({\tilde{x}}_{1},{\tilde{x}}_{2};\tilde{r})=\beta_{1}+{\tilde{x}}_{1}\sum_{j=1}^{5}\alpha_{1,j}\sigma_{\gamma_{j},\tau_{j}}\left(\omega_{j,1}{\tilde{x}}_{1}+\omega_{j,2}{\tilde{x}}_{2}+\psi_{j}\tilde{r}+\theta_{j}\right)\\ \text{and}\;\frac{d{\tilde{x}}_{2}}{dt} & =g_{2}({\tilde{x}}_{1},{\tilde{x}}_{2};\tilde{r})=\beta_{2}+{\tilde{x}}_{2}\sum_{j=1}^{5}\alpha_{2,j}\sigma_{\gamma_{j},\tau_{j}}\left(\omega_{j,1}{\tilde{x}}_{1}+\omega_{j,2}{\tilde{x}}_{2}+\psi_{j}\tilde{r}+\theta_{j}\right), \end{array}$$

whereas the full ODEs read

(C 22) $$\begin{array}{rlrl} \frac{dx_{1}}{dt} & =\beta_{1}+x_{1}\left(\sum_{j=1}^{5}\alpha_{1,j}y_{j}\right),\;\; & & \;\;\;\frac{dx_{2}}{dt}=\beta_{2}+x_{2}\left(\sum_{j=1}^{5}\alpha_{2,j}y_{j}\right),\\ \mu \frac{dy_{1}}{dt} & =\gamma_{1}+\left(\sum_{i=1}^{2}\omega_{1,i}x_{i}+\psi_{1}r+\theta_{1}\right)y_{1}-\tau_{1}y_{1}^{2},\;\; & & \;\;\;\mu \frac{dy_{2}}{dt}=\gamma_{2}+\left(\sum_{i=1}^{2}\omega_{2,i}x_{i}+\psi_{2}r+\theta_{2}\right)y_{2}-\tau_{2}y_{2}^{2},\\ \mu \frac{dy_{3}}{dt} & =\gamma_{3}+\left(\sum_{i=1}^{2}\omega_{3,i}x_{i}+\psi_{3}r+\theta_{3}\right)y_{3}-\tau_{3}y_{3}^{2},\;\; & & \;\;\;\mu \frac{dy_{4}}{dt}=\gamma_{4}+\left(\sum_{i=1}^{2}\omega_{4,i}x_{i}+\psi_{4}r+\theta_{4}\right)y_{4}-\tau_{4}y_{4}^{2},\\ \mu \frac{dy_{5}}{dt} & =\gamma_{5}+\left(\sum_{i=1}^{2}\omega_{5,i}x_{i}+\psi_{5}r+\theta_{5}\right)y_{5}-\tau_{5}y_{5}^{2}\;\text{and} & & \;\;\;\frac{dr}{dt}=0. \end{array}$$

Here, we assume general initial concentrations: $x_{1}(0)=a_{1}\in [0.01,3]$ and $x_{2}(0)=a_{2}\in [0.01,3]$, and $y_{1}(0)=b_{1}\geq 0$, $y_{2}(0)=b_{2}\geq 0$, $y_{3}(0)=b_{3}\geq 0$, $y_{4}(0)=b_{4}\geq 0$, $y_{5}(0)=b_{5}\geq 0$ and r(0)≥0. The coefficients were rounded to three decimal places to quote in text, and the full precision coefficients are available in the code repository.

***Step 2.* Quasi-static classification CRN.** We now demonstrate the training of the classification component of the classification-controlled RNCRN described in figure 7c. We define a target map from the input species $\Lambda_{1}$ to the bifurcation species as

(C 23) $$\begin{array}{r} \bar{r}(\bar{\lambda_{1}})=\left\{ \begin{array}{clclc} & 0.1, & \; & \text{ for all }{\bar{\lambda }}_{1}\in [0,2.5], & \\ & 1, & \; & \text{ for all }{\bar{\lambda }}_{1}>2.5. & \end{array}\right. \end{array}$$

We approximate the target map, equation (C 23), by training a small feed-forward neural network with the chemical activation function, $\sigma_{\gamma }$, i.e.

(C 24) $$\begin{array}{rl} {\tilde{z}}_{1} & =\sigma_{\gamma }\left(\omega_{1}^{\left(z\right)}\tilde{\lambda_{1}}+\theta_{1}^{\left(z\right)}\right),\\ {\tilde{z}}_{2} & =\sigma_{\gamma }\left(\omega_{2}^{\left(z\right)}\tilde{\lambda_{1}}+\theta_{2}^{\left(z\right)}\right),\\ {\tilde{z}}_{3} & =\sigma_{\gamma }\left(\omega_{3}^{\left(z\right)}\tilde{\lambda_{1}}+\theta_{3}^{\left(z\right)}\right)\\ \text{and}\;\tilde{r} & =\sigma_{\gamma }\left(\omega_{1}^{\left(r\right)}{\tilde{z}}_{1}+\omega_{2}^{\left(r\right)}{\tilde{z}}_{2}+\omega_{3}^{\left(r\right)}{\tilde{z}}_{3}+\theta_{0}^{\left(r\right)}\right). \end{array}$$

An RRE with a steady state equivalent to equation (C 24) can be constructed at equilibrium from a series of chemical perceptrons [56]. We introduce the same time-scale separation parameter, μ, as in the RNCRN so that we have the RRE

(C 25) $$\begin{array}{rlrl} \frac{d\lambda_{1}}{dt} & =0, & & \;\;\;\;\lambda_{1}(0)\geq 0,\\ \mu \frac{dz_{1}}{dt} & =\gamma +\left(\omega_{1}^{\left(z\right)}\lambda_{1}+\theta_{1}^{\left(z\right)}\right)z_{1}-z_{1}^{2}, & & \;\;\;\;z_{1}(0)=c_{1}\geq 0,\\ \mu \frac{dz_{2}}{dt} & =\gamma +\left(\omega_{2}^{\left(z\right)}\lambda_{1}+\theta_{2}^{\left(z\right)}\right)z_{2}-z_{2}^{2}, & & \;\;\;\;z_{2}(0)=c_{2}\geq 0,\\ \mu \frac{dz_{3}}{dt} & =\gamma +\left(\omega_{3}^{\left(z\right)}\lambda_{1}+\theta_{3}^{\left(z\right)}\right)z_{3}-z_{3}^{2}, & & \;\;\;\;z_{3}(0)=c_{3}\geq 0\\ \text{and}\;\mu \frac{dr}{dt} & =\gamma +\left(\omega_{1}^{\left(r\right)}z_{1}+\omega_{2}^{\left(r\right)}z_{2}+\omega_{3}^{\left(r\right)}z_{3}+\theta_{0}^{\left(r\right)}\right)r-r^{2}, & & \;\;\;\;r(0)=d\geq 0. \end{array}$$

Using standard backpropogation techniques [78] to train equation (C 24) using data sampled from equation (C 23), we were able to find parameter values γ=1, $\omega_{1}^{\left(z\right)}=-3.899$, $\omega_{2}^{\left(z\right)}=-4.832$, $\omega_{3}^{\left(z\right)}=-5.089$, $\theta_{1}^{\left(z\right)}=10.786$, $\theta_{2}^{\left(z\right)}=11.864$, $\theta_{3}^{\left(z\right)}=12.527$, $\omega_{1}^{\left(r\right)}=5.960$, $\omega_{2}^{\left(r\right)}=-5.255$, $\omega_{3}^{\left(r\right)}=-7.505$ and $\theta_{0}^{\left(r\right)}=0.436$.

***Step 3.* Dynamical approximation.** We now combined the RREs to form the complete classification-controlled RNCRN. Using the rate constants defined in equations (C 20) and equation (C 25), the RREs are

(C 26) $$\begin{array}{rlrl} \frac{d\lambda_{1}}{dt} & =0, & & \;\;\frac{dx_{1}}{dt}=\beta_{1}+x_{1}\left(\sum_{j=1}^{5}\alpha_{1,j}y_{j}\right),\;\;\\ \frac{dx_{2}}{dt} & =\beta_{2}+x_{2}\left(\sum_{j=1}^{5}\alpha_{2,j}y_{j}\right), & & \;\;\mu \frac{dz_{1}}{dt}=\gamma +\left(\omega_{1}^{\left(z\right)}\lambda_{1}+\theta_{1}^{\left(z\right)}\right)z_{1}-z_{1}^{2},\\ \mu \frac{dz_{2}}{dt} & =\gamma +\left(\omega_{2}^{\left(z\right)}\lambda_{1}+\theta_{2}^{\left(z\right)}\right)z_{2}-z_{2}^{2}, & & \;\;\mu \frac{dz_{3}}{dt}=\gamma +\left(\omega_{3}^{\left(z\right)}\lambda_{1}+\theta_{3}^{\left(z\right)}\right)z_{3}-z_{3}^{2},\\ \mu \frac{dr}{dt} & =\gamma +\left(\omega_{1}^{\left(r\right)}z_{1}+\omega_{2}^{\left(r\right)}z_{2}+\omega_{3}^{\left(r\right)}z_{3}+\theta_{0}^{\left(r\right)}\right)r-r^{2}, & & \;\;\mu \frac{dy_{1}}{dt}=\gamma_{1}+\left(\sum_{i=1}^{2}\omega_{1,i}x_{i}+\psi_{1}r+\theta_{1}\right)y_{1}-\tau_{1}y_{1}^{2},\\ \mu \frac{dy_{2}}{dt} & =\gamma_{2}+\left(\sum_{i=1}^{2}\omega_{2,i}x_{i}+\psi_{2}r+\theta_{2}\right)y_{2}-\tau_{2}y_{2}^{2}, & & \;\;\mu \frac{dy_{3}}{dt}=\gamma_{3}+\left(\sum_{i=1}^{2}\omega_{3,i}x_{i}+\psi_{3}r+\theta_{3}\right)y_{3}-\tau_{3}y_{3}^{2},\\ \mu \frac{dy_{4}}{dt} & =\gamma_{4}+\left(\sum_{i=1}^{2}\omega_{4,i}x_{i}+\psi_{4}r+\theta_{4}\right)y_{4}-\tau_{4}y_{4}^{2}\;\text{and} & & \;\;\mu \frac{dy_{5}}{dt}=\gamma_{5}+\left(\sum_{i=1}^{2}\omega_{5,i}x_{i}+\psi_{5}r+\theta_{5}\right)y_{5}-\tau_{5}y_{5}^{2}. \end{array}$$

The reduced vector field, i.e. equation (C 26), as μ→0 is

(C 27) $$\begin{array}{rl} \tilde{\lambda_{1}}(t) & =\tilde{\lambda_{1}}(0),\\ {\tilde{z}}_{1} & =\sigma_{\gamma }\left(\omega_{1}^{\left(z\right)}\tilde{\lambda_{1}}+\theta_{1}^{\left(z\right)}\right),\\ {\tilde{z}}_{2} & =\sigma_{\gamma }\left(\omega_{2}^{\left(z\right)}\tilde{\lambda_{1}}+\theta_{2}^{\left(z\right)}\right),\\ {\tilde{z}}_{3} & =\sigma_{\gamma }\left(\omega_{3}^{\left(z\right)}\tilde{\lambda_{1}}+\theta_{3}^{\left(z\right)}\right),\\ \tilde{r} & =\sigma_{\gamma }\left(\omega_{1}^{\left(r\right)}{\tilde{z}}_{1}+\omega_{2}^{\left(r\right)}{\tilde{z}}_{2}+\omega_{3}^{\left(r\right)}{\tilde{z}}_{3}+\theta_{0}^{\left(r\right)}\right),\\ \frac{d{\tilde{x}}_{1}}{dt} & =g_{1}({\tilde{x}}_{1},{\tilde{x}}_{2};r)=\beta_{1}+{\tilde{x}}_{1}\sum_{j=1}^{5}\alpha_{1,j}\sigma_{\gamma_{j},\tau_{j}}\left(\omega_{j,1}{\tilde{x}}_{1}+\omega_{j,2}{\tilde{x}}_{2}+\psi_{j}r+\theta_{j}\right)\\ \text{and}\;\frac{d{\tilde{x}}_{2}}{dt} & =g_{2}({\tilde{x}}_{1},{\tilde{x}}_{2};r)=\beta_{2}+{\tilde{x}}_{2}\sum_{j=1}^{5}\alpha_{2,j}\sigma_{\gamma_{j},\tau_{j}}\left(\omega_{j,1}{\tilde{x}}_{1}+\omega_{j,2}{\tilde{x}}_{2}+\psi_{j}r+\theta_{j}\right). \end{array}$$

## Appendix D. Training algorithms

We now present various versions of the training algorithms used throughout this paper to train single-layer RNCRNs, parametrized RNCRNs and classification-controlled RNCRNs.

**Remark.** The training algorithms discussed in this appendix all leverage a tolerance parameter, ε, to control the allowable error in the intermediate quasi-static approximation step. The required value of this hyperparameter will vary according to the sensitivity and the concentration scale of the target system. For example, for the homoclinic bifurcation example, given as equation (3.5), a smaller tolerance of $\varepsilon =10^{-3}$ was sufficient. Some other systems are more robust, such as the Hopf bifurcation example, given as equation (3.4), for which $\varepsilon =10^{-1}$ was sufficient. In this work, we do not try to select the hyperparameter *a priori*. In this paper, we start with a relatively large tolerance, and then run a hyperparameter sweep, whereby the tolerance is reduced in each run of the algorithm until satisfactory convergence, i.e. until the quasi-static vector field qualitatively replicates the target behaviour. Heuristically, the starting tolerance was fixed to a value around 100 times less than the magnitude of the target vector field averaged across the region of interest.

### D.1. Algorithm 2. Two-step algorithm for training *parametrized RNCRNs* to approximate bifurcations

In §3 and appendix A, we present examples of parametrized RNCRN trained by algorithm 2.

### D.2. Pseudocode for algorithm 2. Two-step algorithm for training *parametrized RNCRNs* to approximate bifurcations

We now provide a pseudocode description to clarify the precise input, outputs and loss evaluation used in algorithm 2.

Pseudocode 1: Pseudocode detailing the quasi-static approximation step of algorithm 2 using the Adamax optimizer [79] with mean squared error (m.s.e.) loss.

### D.3. Algorithm 3. Two-step algorithm for training *parametrized RNCRNs* to approximate piecewise systems

In §4 and appendix B, we trained example systems using a modified version of algorithm 2 to approximate piecewise systems. Note, in this section we also trained the *parametrized RNCRN* with AD  [80,81] presented here, for clarity, as algorithm 3. This expanded algorithm uses the most general form of the parametrized RNCRN

(D 1) $$\begin{array}{rlrl} \frac{dx_{i}}{dt} & =\beta_{i}+x_{i}\sum_{j=1}^{M}\alpha_{i,j}y_{j}, & & \;\;\;\text{ for }i=1,\ldots ,N,\\ \frac{d\lambda_{l}}{dt} & =0, & & \;\;\;\text{ for }l=1,\ldots ,L\\ \text{and}\;\frac{dy_{j}}{dt} & =\frac{\gamma_{j}}{\mu }+\left(\sum_{i=1}^{N}\frac{\omega_{j,i}}{\mu }x_{i}+\sum_{l=1}^{L}\frac{\psi_{j,l}}{\mu }\lambda_{l}+\frac{\theta_{j}}{\mu }\right)y_{j}-\frac{\tau_{j}}{\mu }y_{j}^{2}, & & \;\;\;\text{ for }j=1,\ldots ,M, \end{array}$$

where $x_{i}=x_{i}(t)\geq 0$, $y_{j}=y_{j}(t)\geq 0$, $\lambda_{l}=\lambda_{l}(t)=\lambda_{l}(0)\geq 0$, $\beta_{i}\geq 0$, $\alpha_{i,j}\in R$, $\gamma_{j}>0$, $\tau_{j}>0$, $\omega_{ji}\in R$, $\theta_{j}\in R$ and $\psi_{j,l}\in R$.

These AD techniques are just an alternative method for optimizing the reduced vector field so they do not change the conceptual purpose of the quasi-static approximation step. However, they allow for optimization over more reaction coefficients in the RNCRN. The use of AD techniques allowed the formulation of the optimization function over the reduced vector field directly, i.e. $g_{i}({\tilde{x}}_{1},{\tilde{x}}_{2},\ldots ,{\tilde{x}}_{N})\approx f_{i}({\tilde{x}}_{1},{\tilde{x}}_{2},\ldots ,{\tilde{x}}_{N})$, rather than having to optimize over a transformed target vector field $(f_{i}({\tilde{x}}_{1},{\tilde{x}}_{2},\ldots ,{\tilde{x}}_{N})-\beta_{i})/{\tilde{x}}_{i}\approx \sum_{j=1}^{M}\alpha_{i,j}^{*}\sigma_{\gamma }\left(\sum_{k=1}^{N}\omega_{j,k}^{*}{\tilde{x}}_{k}+\theta_{j}^{*}\right)$. Therefore, several rate constants that we used to fix arbitrarily prior to the quasi-static approximation are now free to be optimized (i.e. $\beta_{i}$ and γ). Additionally, we allow the constant production reaction, $\gamma_{j}>0$, and the self-sequestration reaction, $\tau_{j}>0$, to take independent values for each reaction j=1,2,…,M.

Including these new parameters induces a slightly different chemical activation function in the reduced vector field that is defined as

(D 2) $$\begin{array}{r} \sigma_{\gamma_{j},\tau_{j}}(A)=\frac{1}{2\tau_{j}}\left(A^{2}+\sqrt{A^{2}+4\tau_{j}\gamma_{j}}\right). \end{array}$$

### D.5. Algorithm 5. Two-step algorithm for training the *RNCRN* to approximate data-defined limit cycles

In §5 and appendix C, we trained example systems using a modified versions of algorithm 1 from [51] on data from algorithm 1 in this paper. For clarity, we present this as algorithm 5 and note that it involved both AD techniques and, owing to not having an ODE system to validate trajectories, an abridged dynamical approximation step with an informal inspection-based termination.

We allow the constant production reaction, $\gamma_{j}>0$, and the self-sequestration reaction, $\tau_{j}>0$, to take independent values for each reaction j=1,2,…,M. The full RNCRN in this situation is

(D 3) $$\begin{array}{rlrlrlr} \frac{dx_{i}}{dt} & =\beta_{i}+x_{1}\sum_{j=1}^{M}\alpha_{i,j}y_{j}, & & \;x_{i}(0)=a_{i}, & & & \;\;\;\text{ for }i=1,\ldots ,N\\ \text{and}\;\frac{dy_{j}}{dt} & =\frac{\gamma_{j}}{\mu }+\left(\sum_{i=1}^{N}\frac{\omega_{j,i}}{\mu }x_{i}+\frac{\theta_{j}}{\mu }\right)y_{j}-\frac{\tau_{j}}{\mu }y_{j}^{2}, & & \;y_{j}(0)=b_{j}, & & & \;\;\;\text{ for }j=1,\ldots ,M. \end{array}$$

Including these new parameters induces a slightly different chemical activation function in the reduced vector field that is defined as in equation (D 2).
